# Calcium(ii)-catalyzed enantioselective conjugate additions of amines†
†We thank Dow Chemical Company for generous support of this work.
‡
‡Electronic supplementary information (ESI) available: Experimental procedures, characterization of new compounds, and spectroscopic data. CCDC 1531303, 1531265. For ESI and crystallographic data in CIF or other electronic format see DOI: 10.1039/c7sc05205g


**DOI:** 10.1039/c7sc05205g

**Published:** 2018-01-10

**Authors:** Brice E. Uno, Rachel D. Dicken, Louis R. Redfern, Charlotte M. Stern, Greg G. Krzywicki, Karl A. Scheidt

**Affiliations:** a Department of Chemistry , Center for Molecular Innovation and Drug Discovery , Northwestern University , 2145 Sheridan Rd , Evanston , IL 60208 , USA . Email: scheidt@northwestern.edu

## Abstract

The direct enantioselective chiral calcium(ii)·phosphate complex (Ca[CPA]_2_)-catalyzed conjugate addition of unprotected alkyl amines to maleimides was developed. This mild catalytic system represents a significant advance towards the general convergent asymmetric amination of α,β-unsaturated electrophiles, providing medicinally relevant chiral aminosuccinimide products in high yields and enantioselectivities. Furthermore, the catalyst can be reused directly from a previously chromatographed reaction and still maintain both high yield and selectivity.

## Abstract 2

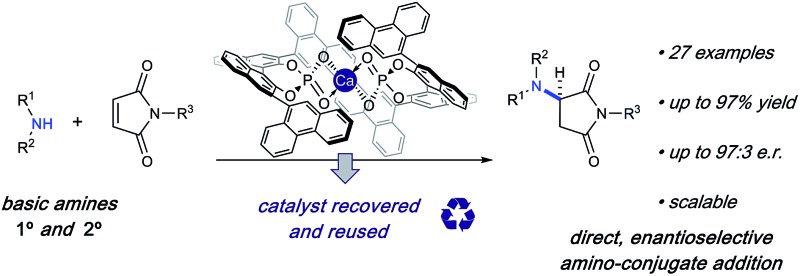
The direct enantioselective chiral calcium(ii)·phosphate complex (Ca[CPA]_2_)-catalyzed conjugate addition of unprotected alkyl amines to maleimides was developed.

## 

Chiral amines are a ubiquitous motif in pharmaceuticals and natural products (Fig. 1).^1^ The conjugate addition of amine nucleophiles to various α,β-unsaturated systems is a well-established transformation to access the corresponding β-amino carbonyl products.^2^ However, catalytic enantioselective methods for the construction of C–N bonds directly from amines remain a challenge in synthetic organic chemistry. Direct conjugate additions of amines with α,β-unsaturated electrophiles have been shown to proceed at high temperatures and pressures;^3^ however the reversibility of the initial attack by the amine eventually leads to racemic products (Fig. 1A).^4^ Stoichiometric homochiral lithium amides can be successfully deployed under kinetic control, achieving high yield and selectivity. However, these sensitive, strongly basic reagents are further limited by the need to remove the chiral α-methylbenzyl moiety to carry the products forward to useful targets.^5^ To circumvent these issues, current catalytic methods have relied upon the use of non-basic nitrogen nucleophiles as amine surrogates to avoid catalyst poisoning (Fig. 1B),^6^ which is common when basic amines are used as reagents in the presence of chiral Lewis or Brønsted acidic catalysts.^7^ Therefore, numerous examples of non-basic nitrogen nucleophiles including azides,^8^ hydroxylamines,^9^ O-functionalized carbamates,^10^ 1,2,4-triazoles,^11^ indoles,^12^ and anilines^13^ have been strategically deployed to avoid Lewis acid complexation,^14^ Brønsted acid neutralization, or unselective iminium activation.10*a* However, in all of these cases, a protected nitrogen atom is installed which requires multiple steps to elaborate further. Thus, a more convergent approach would be enabled by the direct asymmetric amination of basic primary and secondary amines without the use of protecting groups.

**Fig. 1:**
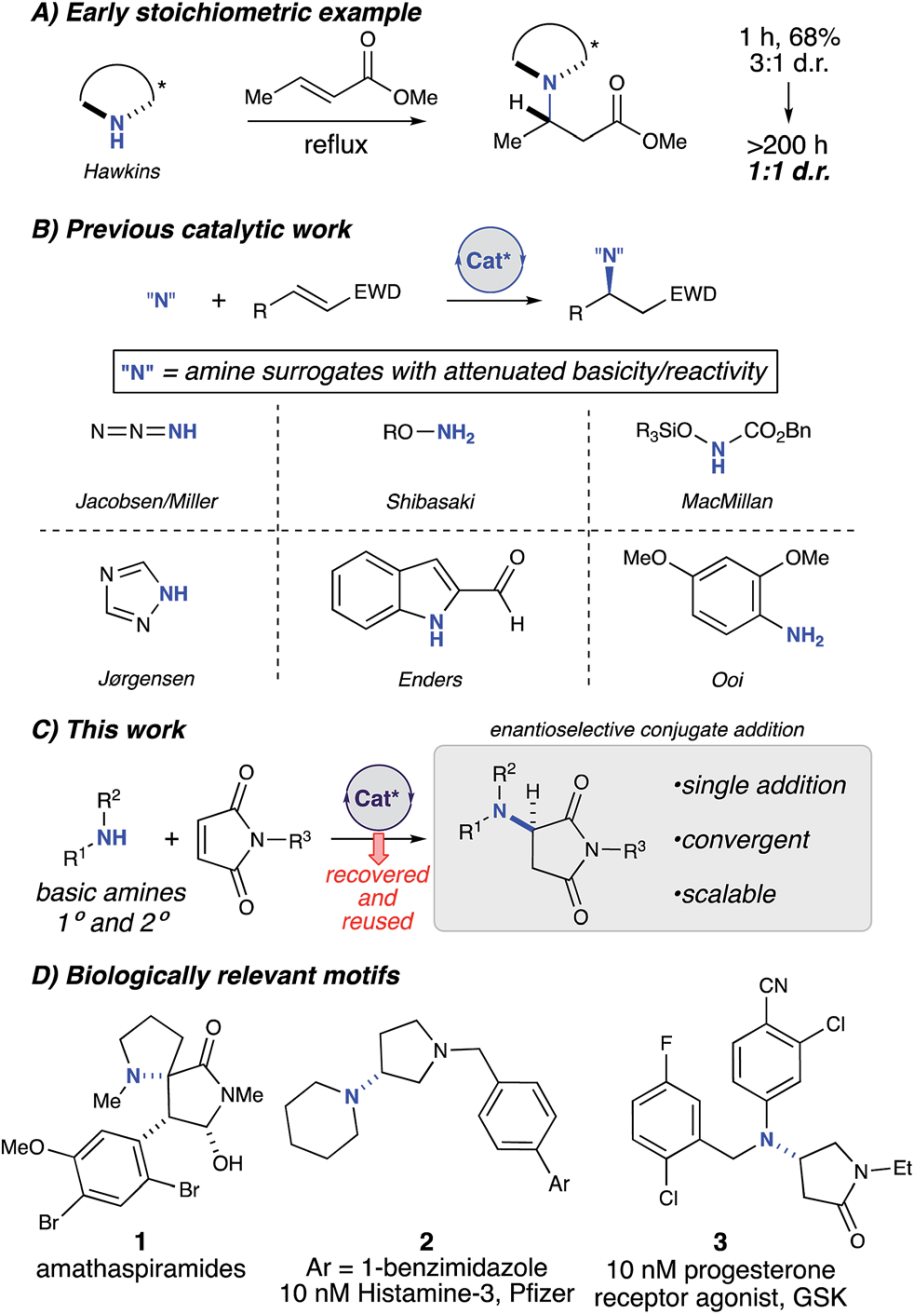
Enantioselective conjugate additions of amines.

Apparently, there are only three examples of catalytic asymmetric amino-conjugate additions that have successfully employed alkyl amines.^15^ In 2003, Togni briefly explored asymmetric amino-conjugate additions to activated olefins as the initial step in a catalytic asymmetric hydroamination reaction catalysed by a novel chiral Ni(ii) phosphine complex.15*b* Morpholine and piperidine produced modestly enantioenriched products when reacted with methacrylonitrile (69% and 20% ee, respectively), which represents the first significant example of an effective, enantioselective intermolecular hydroamination reaction employing alkyl amines. Despite this promising proof-of-concept study, general asymmetric amino-conjugate additions with unfunctionalized/masked amines remain unrealized, which underscores the fundamental challenge associated with the use of highly basic and sterically unencumbered reagents in conjunction with Lewis acidic metal catalysts. In 2015, Huang and co-workers reported an efficient, highly enantioselective conjugate addition of primary alkyl amines to activated β-aryl β-trifluoromethyl nitroolefins.15*c* Unlike most catalytic examples, their strategy uses chiral Brønsted base catalysis.^16^ However, a major limitation of this method is the lack of secondary amines as nucleophiles. Additionally, strongly basic and cryogenic conditions are required, which potentially limit the generality of this transformation.15*c* Therefore, we sought mild catalytic conditions capable of providing enantioenriched amino-conjugate addition products from a general set of readily available alkyl amines with maleimides, which were chosen as an ideal substrate for catalyst identification and optimization due to their ready availability and excellent conjugate acceptor properties (Fig. 1C). Additionally, enantioenriched aminosuccinimide products serve as an easily functionalized scaffold to generate aminolactams and aminopyrrolidines.^17^ Aminosuccinimides and their derivatives are also a common motif in bioactive small molecules, pharmaceuticals, and natural products (Fig. 1D).^18^ Access to these products from achiral starting materials can facilitate the rapid generation of diverse small molecule libraries aimed at probing new chemical spaces.

We began our studies with a reaction between equimolar quantities of *N*-benzylmaleimide and *p*-tolylamine. Our primary focus was to enhance the enantioselectivity of the title reaction (Table 1). An initial exhaustive screen of various asymmetric catalyst families including hydrogen bond donors (HBD), metal-TADDOL complexes, metal BINOL-complexes, and chiral phosphoric acids (CPA), identified that CPA **A**-H possessing 1-napthyl substitution at the 3,3′-positions has the capability to produce the title compound in modest yield and selectivity (entry 1). Subsequently, we investigated the role of water in the reaction and observed that the addition of 4 Å MS had a moderate but reproducible impact on selectivity (entry 2). We then investigated a wide range of desiccants9*b* and found that calcium oxide had a greater than anticipated positive effect on the selectivity of the reaction (entry 3).^19^ Additionally, we observed a moderate increase in e.r. over time to 80 : 20 e.r. (entry 4). We therefore hypothesized that the reaction of calcium oxide with **A**-H led to the formation of a more enantioselective calcium phosphate catalyst. Our hypothesis was enlightened by combining the prior elegant work of Ishihara, Antilla, and Rueping who demonstrated the role of catalytic chiral alkali metal and alkaline earth metal-phosphate salts in various reactions.^20^ Thus, we investigated two pre-formed calcium phosphate complexes (entries 5 & 6) and observed that the calcium CPA complex possessing 9-phenanthracenyl substitution on the phosphate 3,3′-positions, Ca[**B**]_2_ (Table 1), facilitated the title reaction in 76% yield and 95 : 5 e.r. (entry 6). Strikingly, removal of the 4 Å MS diminished both yield and selectivity (entry 7). After investigating selectivity as a function of temperature (entries 8 & 9), we looked at other CPA salts (entries 10–12) and determined that Ca[**B**]_2_ was indeed optimal. We then compared calcium and magnesium phosphate complexes, and demonstrated again that Ca[**B**]_2_ was optimal (entry 13). Furthermore, increasing its concentration to 0.05 M and lowering the catalyst loading to 5 mol% increased the yield to 95% with 94 : 6 e.r. (entries 14 & 15).

**Table 1:** Optimization of the amino-conjugate addition reaction

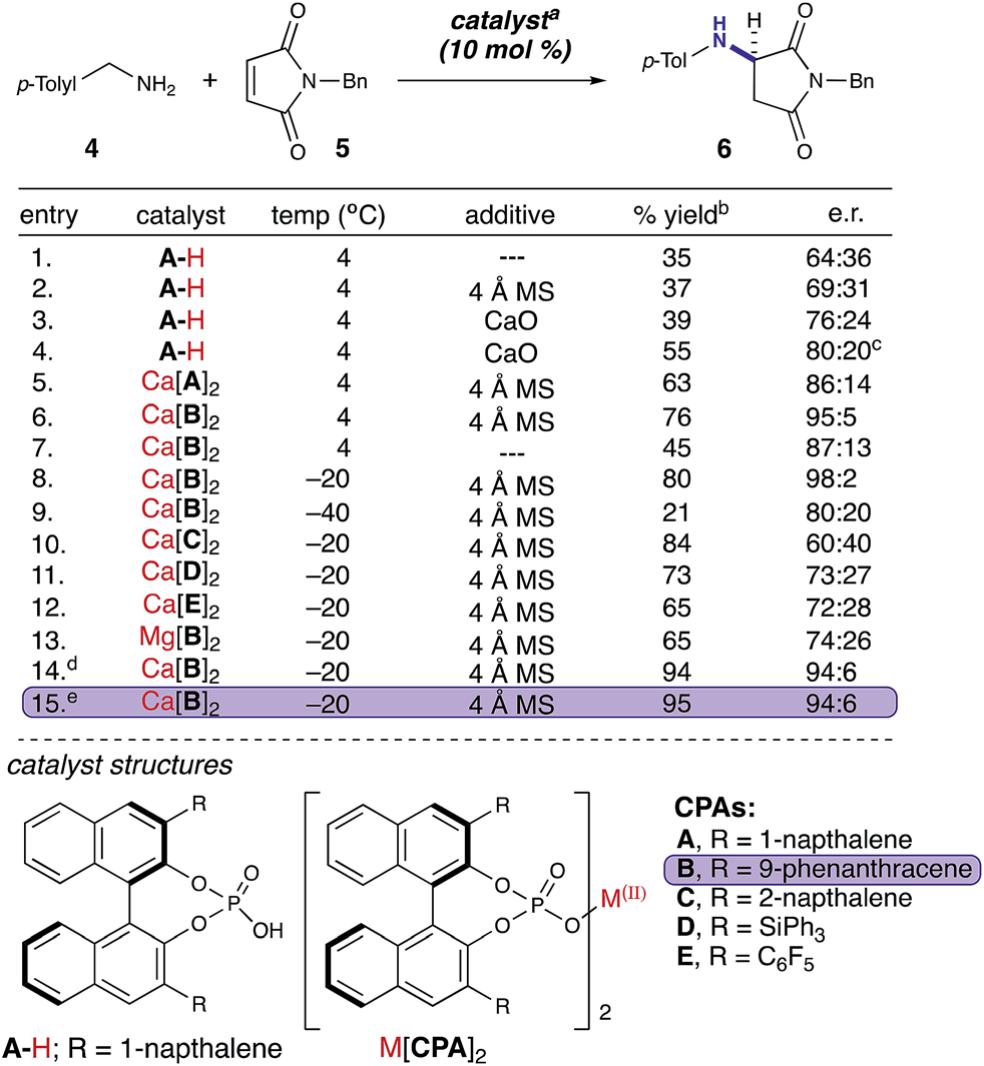

^*a*^0.025 mmol scale, toluene 0.02 M, 18 h.

^*b*^NMR yields with 1,3,5-trimethoxybenzene as an internal standard.

^*c*^Time point at 48 h.

^*d*^Toluene 0.05 M.

^*e*^Toluene 0.05 M, 5 mol% catalyst loading of Ca[**B**]_2_.

With the optimized conditions in hand, we next investigated the scope of the reaction with a range of aliphatic amines and maleimides (Table 2). Para-substituted primary benzylamines with a range of electron donating and withdrawing groups afforded the conjugate addition products (**6–11**) in 93 : 7–94 : 6 e.r. and 77–91% yield. *Meta*- and *ortho*-substituted benzyl amines afforded **12** and **13** in similar yields and selectivities. The products derived from less sterically bulky amines and linear amines were obtained with lower enantioselectivity (**14–17**) and moderate yields. In contrast, bulkier amines gave products **18** and **19** in high yield and selectivity. Notably, secondary cyclic amines provided conjugated products **20–24** in 93 : 7–97 : 3 e.r. These substrates would be difficult to access *via* other methodologies or from an enantiopure amino acid derived starting material.^21^ The enantioselectivity for the arylpiperidine-derived **24** uniquely improved at –40 °C which was not general for the other substrates.

**Table 2:** Substrate scope^*a*^

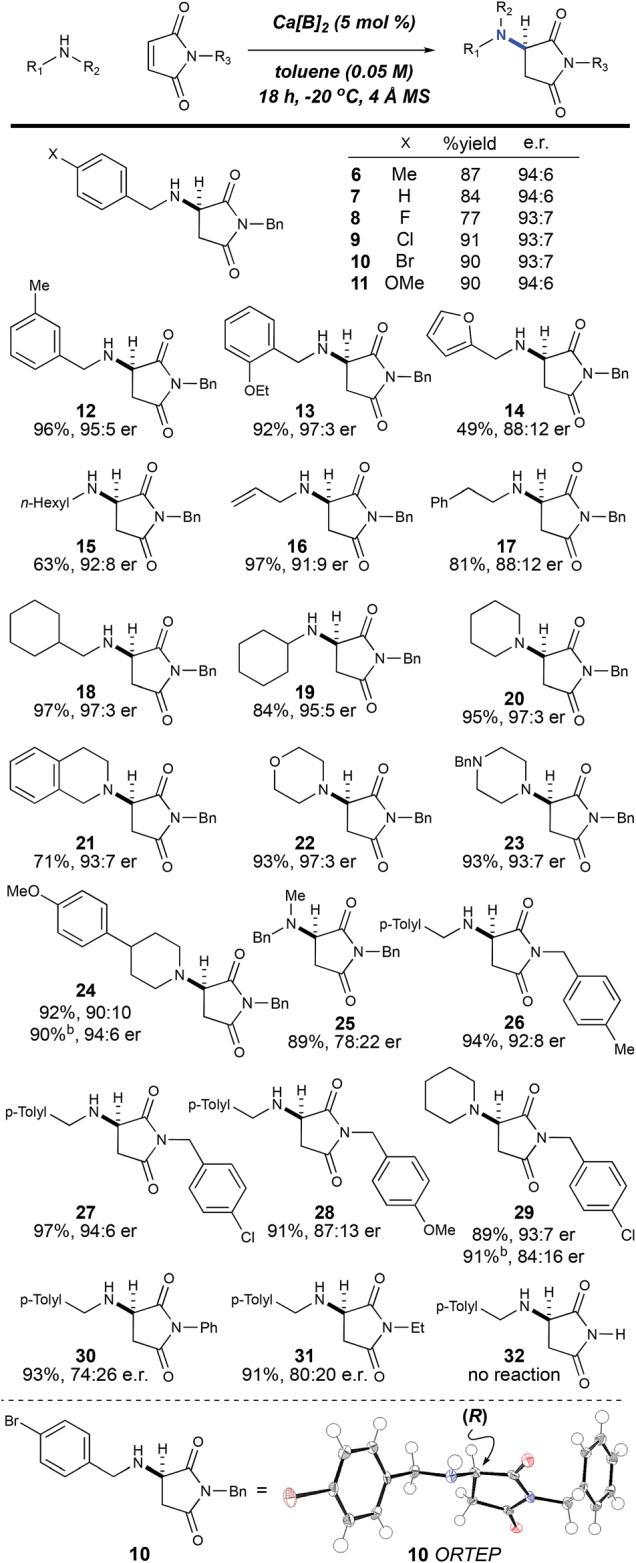

^*a*^Isolated yields on 0.2 mmol scale.

^*b*^Reaction run at –40 °C for 24 h.

Acyclic secondary amines showed the lowest selectivity among the nucleophiles (**25**). Also, substitutions on the benzyl maleimide were tolerated (**26–28**). The cross-reaction between piperidine and a substituted benzyl maleimide generated product **29** in good yield and selectivity. *N*-Phenyl maleimide was a poor substrate with regard to selectivity (74 : 26 e.r.); however, the desired 1,4-addition product **30** was synthesized in 93% yield with no observed 1,2-addition product (a common side-reaction with *N*-aryl maleimides).^22^ Maleimide substrates with smaller appendages were observed to react with lower selectivities (**31**). The unsubstituted maleimide product **32** was not observed, presumably due to a lack of solubility.

The title reaction was successfully scaled up by 1000-fold from the initial screening conditions (Scheme 1). Taking into account the observed dependence of enantioselectivity on concentration, the amine nucleophile was added slowly to the other reaction components *via* a cannula. These conditions afforded 7.15 g of the product (93% yield) in 94 : 6 e.r. The product was successfully recrystallized to >99 : 1 e.r. Additionally, >95% of the catalyst Ca[**B**]_2_ was recovered *via* column purification. The recovered Ca[**B**]_2_ was subsequently able to reproduce the title reaction without loss of yield or selectivity. The ability to directly recover and reuse Ca[**B**]_2_ from each reaction at >95% efficiency gives this methodology more utility, especially given the high molecular weight of the catalyst.

**Scheme 1:**
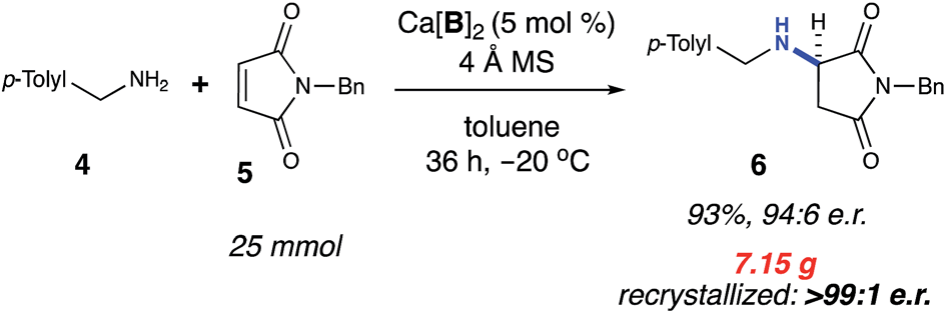
Reaction scale-up. ^a^Isolated yield on 25 mmol scale using 1.1 equivalents of **4**. Active catalyst was recovered after chromatography (96%, 1.6 g).

In an effort to rationalize the observed enantioselectivity, we obtained X-ray crystal structure and ^31^P NMR spectroscopy data for the pre-formed calcium phosphate complex used in our optimization and scope studies (Fig. 2B).^23^ Surprisingly, the observed structure shows a 4 : 2 ratio of **B** to Ca^2+^, not a Ca[**B**]_2_ complex. Additionally, both calcium atoms are coordinatively saturated, with each cation bound to five molecules of water, which creates a hydrogen-bonding network. Although it is possible that the observed ORTEP structure is the actual catalytic species, we hypothesize that it is more likely a precatalyst is activated in the presence of molecular sieves. This observation is supported by the significant change in the ^31^P NMR spectrum in the presence of 4 Å MS (Fig. 2B). The yield and selectivity of the reaction also diminished in the absence of the 4 Å MS (Table 1, entry 7), which supports that dehydration of the Ca_2_[**B**]_4_·(H_2_O)_10_ complex is necessary. Interestingly, when all the reaction components are present, the ^31^P NMR data is reminiscent of the precatalyst (Fig. 2B). This data indicates that the presence of the amine re-establishes the hydrogen-bonding network that is lost upon dehydration of the Ca_2_[**B**]_4_·(H_2_O)_10_ complex. Understanding this Lewis base/Lewis acid interaction between the active catalyst and a coordinated maleimide substrate will require further investigation.

**Fig. 2:**
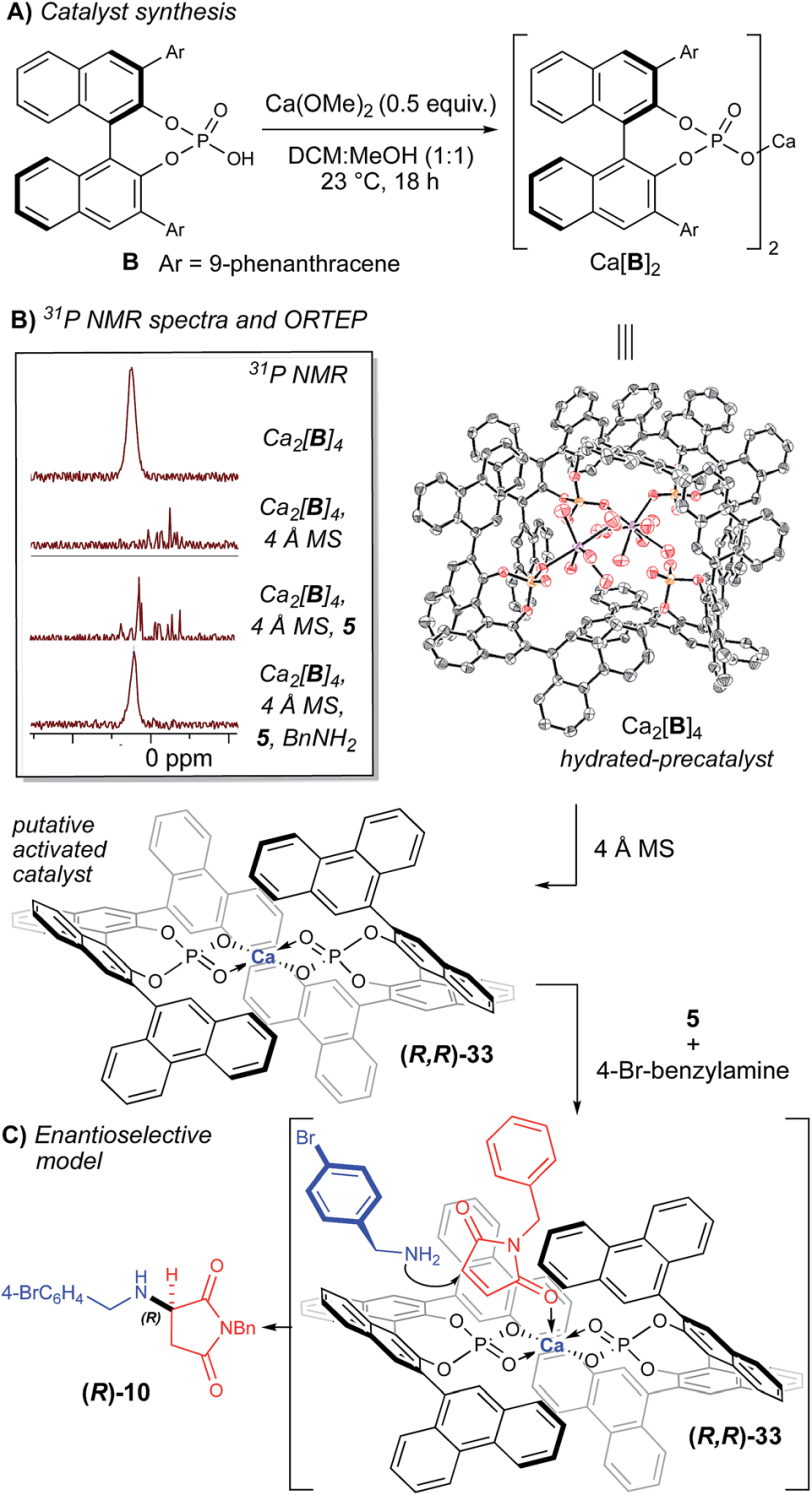
Synthesis and characterization of Ca[**B**]_2_. (A) Synthesis of Ca[**B**]_2_. (B) ORTEP of putative precatalyst at 80% probability and ^31^P NMR shifts with and without 4 Å MS. (C) Proposed stereoinduction model supported by the observed absolute stereochemistry from the ORTEP structure of **10**.

Based on the obtained spectroscopic data, we hypothesize that the Ca_2_[**B**]_4_·(H_2_O)_10_ complex is activated *via* dehydration in the presence of 4 Å MS, inducing it to reorganize to form Ca[**B**]_2_ complex **33** (observed by HRMS, see ESI‡). The extent of dehydration of Ca_2_[**B**]_4_·(H_2_O)_10_ required to form the active catalyst cannot be quantified by these experiments; however, it is reasonable to postulate that the loss of some coordinating water ligands from the Ca_2_[**B**]_4_·(H_2_O)_10_ complex should open up Lewis acidic sites on the calcium atom, which are then able to coordinate the amine nucleophile. Based on structure **33**, we propose a model for enantioselectivity, where the *si*-face of maleimide **5** is blocked, which allows the *re*-face attack of the amine nucleophile (Fig. 2C).

After exploring the scope of our conjugate addition with a variety of amines and maleimides, we also applied our methodology to the synthesis of **35**, a potent novel 5-HT_2A_ agonist developed by Acadia Pharmaceuticals (Scheme 2).18*c* Since the binding affinity of **35** was measured as a racemic mixture, we envisioned that our methodology could readily determine the more active enantiomer. Starting from recrystallized **6**, lithium aluminium hydride reduction cleanly produced **34** in 95% yield and >99 : 1 e.r. (Scheme 2). Selective acylation of **34** with 4-methoxyphenylacetic acid produced **35** in 43% yield and >99 : 1 e.r. To further demonstrate the utility of this methodology, we selectively removed the benzylic group on the amine (**36**) as well as selectively deoxygenate the position adjacent to the amine (**37**).

**Scheme 2:**
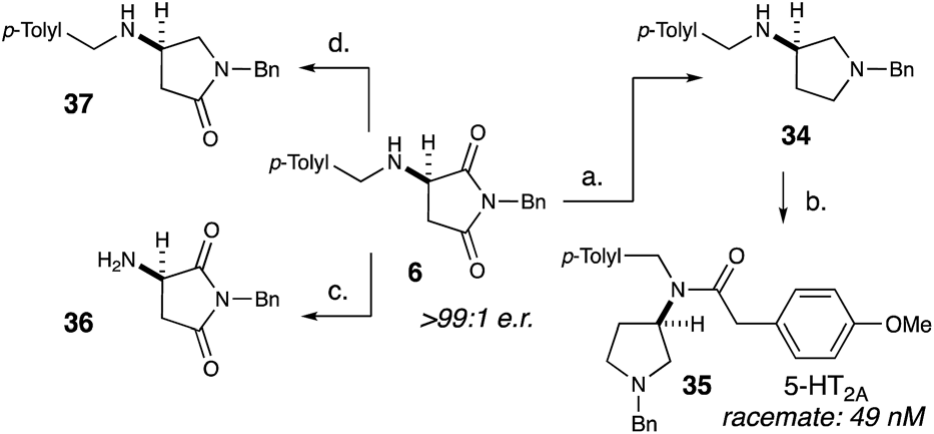
Substrate diversification and target synthesis. Reagents and conditions: (a) LiAlH_4_, THF, reflux, 95%, >99 : 1 e.r. (b) (4-Methoxy)phenylacetyl chloride, DIPEA, 43%, >99 : 1 e.r. (c) Pd/C, H_2_ (1 atm), MeOH, 96%, >99 : 1 e.r. (d) (i) NaBH_4_, DCM : MeOH, 4 °C, 42%; (ii) Et_3_SiH, TFA, DCM, 0 °C, 84%, 97 : 3 e.r.

## Conclusions

In summary, we have discovered an efficient and scalable catalytic asymmetric conjugate addition of unmasked and unfunctionalized amines to maleimides. This process accommodates both primary and secondary amines, which underscores the unusual compatibility of these Lewis basic nucleophiles with the Lewis acidic Ca^2+^ complex. Crystallographic studies indicate an initial Ca_2_[**B**]_4_ species is formed through the reaction of a chiral phosphoric acid and calcium(ii) methoxide. Further spectroscopic studies indicate that a dynamic process is involved, where molecular sieves are required for the observed reactivity and selectivity, which are thought to play a role in the activation of the catalyst. The addition of amine nucleophiles can re-establish a hydrogen-bonding network similar to that found in the hydrated Ca_2_[**B**]_4_·(H_2_O)_10_ complex. Furthermore, although the calcium phosphate catalyst Ca[**B**]_2_ has a relatively high molecular weight, it can be effectively recovered in >95% yield. Future investigations involve continued analysis of the calcium-phosphate dynamics and applications of this reaction in the synthesis of bioactive compounds.

## Conflicts of interest

There are no conflicts to declare.

## Supplementary Material

Supplementary information

Crystal structure data
