# Supplementary material for: Calcium(ii)-catalyzed enantioselective conjugate additions of amines
Source: Chem Sci. 2018 Jan 10;9(6):1634–9. doi: 10.1039/c7sc05205g (PMC5887857; doi:10.1039/c7sc05205g)
Supplement: Supplementary file 1 [file SC-009-C7SC05205G-s001.pdf]

Electronic Supplementary Material (ESI) for Chemical Science.
This journal is © The Royal Society of Chemistry 2017

Calcium-Catalyzed Enantioselective Conjugate Additions of Amines

Brice E. Uno, Rachel D. Dicken, Louis R. Redfern, Charlotte M. Stern, Greg K. Krzywicki, and Karl A. Scheidt*

Department of Chemistry, Center for Molecular Innovation and Drug Discovery
Northwestern University,
2145 Sheridan Road, Evanston, IL 60208 USA

Supplementary Materials

Supporting Information

Table of Contents

General Information **S3**
Synthesis and characterization of Ca[**B**]$_2$........ **S4**
General procedure for the synthesis of aminosuccinimides........ **S5**
Procedure and characterization data for all products........ **S5**
X-ray characterization data........ **S22**
NMR spectroscopic data........ **S24**
HPLC traces of racemic and enantioenriched products........ **S57**

General Information

All reactions were carried out under an ambient atmosphere in non-oven-dried glassware with magnetic stirring. THF, toluene, and DMF were purified by passage through a bed of activated alumina.[1] Reagents were purified prior to use unless otherwise stated following the guidelines of Perrin and Armarego.[2] *N*-tolylamine was distilled from CaH_2. Purification of reaction products was carried out by flash chromatography using EM Reagent silica gel 60 (230-400 mesh). Analytical thin layer chromatography was performed on EM Reagent 0.25 mm silica gel 60-F plates. Visualization was accomplished with UV light and ceric ammonium nitrate stain or potassium permanganate stain followed by heating. Infrared spectra were recorded on a Bruker Tensor 37 FT-IR spectrometer. ^1H NMR spectra were recorded on AVANCE III 500 MHz w/ direct cryoprobe (500 MHz) spectrometer and are reported in ppm using solvent as an internal standard ($CDCl_3$ at 7.26 ppm). Data are reported as (ap = apparent, s = singlet, d = doublet, t = apparent triplet, q = quartet, m = multiplet, b = broad; coupling constant(s) in Hz; integration.) Proton-decoupled ^{13}C NMR spectra were recorded on an AVANCE III 500 MHz w/ direct cryoprobe (125 MHz) spectrometer and are reported in ppm using solvent as an internal standard ($CDCl_3$ at 77.00 ppm). ^{31}P NMR spectra were acquired at 26 °C on a 400 MHz Agilent 400MR-DD2 spectrometer equipped with a OneNMR probe and a 7600AS autosampler; this system was funded by NSF CRIF grant CHE-104873. Mass spectra were obtained on a WATERS Acquity-H UPLC-MS with a single quad detector (ESI) or on a Varian 1200 Quadrupole Mass Spectrometer and Micromass Quadro II Spectrometer (ESI).

Synthesis and Characterization of Ca[B]$_2$

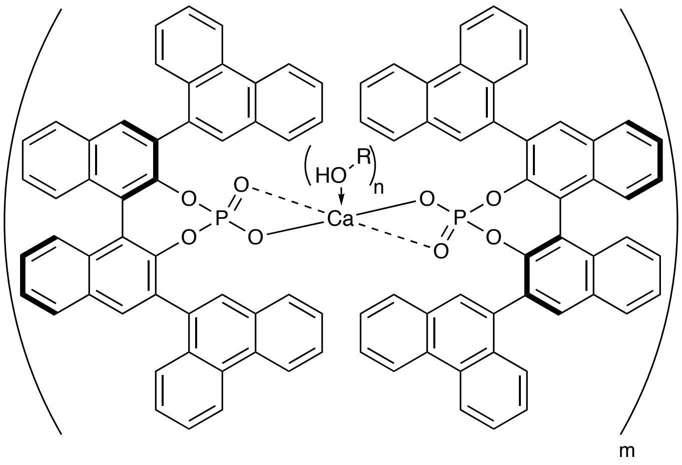

calcium 2,6-di(phenanthren-9-yl)dinaphtho[2,1-d:1',2'-f][1,3,2]dioxaphosphepin-4-olate 4-oxide (Ca[B]$_2$): To a flame-dried flask at 23 °C, charged with dichloromethane:MeOH (1:1, 12 mL), was added **B** [(R) 9,9'-bisphenanthryl-BINOL phosophoric acid (2.57 mmol, 1.80 g, 2.0 equiv)] and freshly powdered calcium methoxide (1.28 mmol, 0.131 g, 1.0 equiv). The reaction was stirred for 24 h at 23 °C at which point the slightly turbid solution was concentrated to dryness, azeotroped with toluene (3 x 15 mL), and placed under high vacuum (~ 0.1 Torr) to yield Ca[**B**]$_2$ as an off white powder (1.28 mmol, 1.84 g, quantitative).

Analytical data for Ca[**B**]$_2$: ^{1}H NMR (500 MHz, DMSO-d$_6$) δ 8.90 (dd, J = 14.0, 8.2 Hz, 8H), 8.06 (m, 16H), 7.57 (m, 36H), 4.11 (q, J = 5.2 Hz, 1H, MeOH), 3.34 (s, 14H, H_2O), 3.17 (d, J = 5.0 Hz, 3H, MeOH). ^{13}C NMR (126 MHz, DMSO-d$_6$) δ 148.4, 148.3, 134.3, 133.3, 132.3, 131.4, 131.2, 130.1, 129.6, 129.6, 129.3, 129.0, 128.3, 126.8, 126.5, 126.3, 124.9, 123.0, 122.7, 122.4, 48.6 (MeOH). HRMS (ESI): Mass calculated for $C_{96}H_{57}CaO_8P_2$ [M+H]: 1439.3155; found: 1439.3149;

General Procedure and Characterization Data for the Synthesis of Aminosuccinimides

In a nitrogen-filled dry box, a screw-cap reaction tube equipped with a magnetic stirbar was charged with the corresponding maleimide **2** (0.2 mmol, 1.0 equiv), calcium phosphate complex catalyst Ca[**B**]$_2$ (14.0 mg, 0.01 mmol, 0.05 equiv), and 4 Å MS (100 mg). The tube was capped with a septum cap, removed from the drybox and put under positive N_2 pressure. Dry toluene (3.0 mL) was then added and the heterogeneous mixture was cooled to −20 °C. A solution of the corresponding amine (0.20 mmol, 1.0 equiv) in toluene (0.9 mL) was added dropwise, and the reaction was stirred for 14 h at spectrum °C. At this point an additional bolus of amine (0.02 mmol, 0.1 equiv) in toluene (0.1 mL) was added. After 18 h, the entire crude reaction mixture at −20 °C was directly transferred onto a SiO_2 column pre-equilibrated with 3:1 Hex:EtOAc. Flash chromatography (gradient 3:1 Hex:EtOAc → 1:1 Hex:EtOAc) afforded the aminosuccinimide product, followed by elution with 10:1 EtOAc:MeOH to recover Ca[**B**]$_2$.

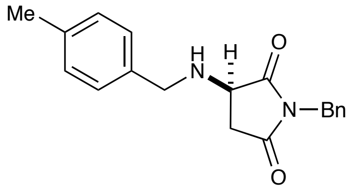

(R)-1-benzyl-3-((4-methylbenzyl)amino)pyrrolidine-2,5-dione (6): Prepared according to the general procedure using *p*-tolylmethylamine (0.027 g, 0.22 mmol, 1.1 equiv) to afford 0.062 g (87% yield) of product as a clear crystalline solid.

Analytical data for **6**: ^{1}H NMR (500 MHz, Chloroform-d) δ 7.24 (m, 10H), 4.65 (s, 2H), 3.79 (m, 3H), 2.85 (dd, *J* = 18.0, 8.3 Hz, 1H), 2.51 (dd, *J* = 18.1, 4.8 Hz, 1H), 2.33 (s, 3H), 2.16 (s, 1H). ^{13}C NMR (126 MHz, Chloroform-d) δ 177.6, 174.9, 137.2, 135.5, 135.5, 129.3, 128.8, 128.7, 128.2, 128.0, 55.4, 51.6, 42.4, 36.4, 21.1. HRMS (ESI): Mass calculated for $C_{19}H_{21}N_2O_2$ [M+H]: 309.1603; found: 309.1603; IR (thin film) 3302, 3291, 2913, 2846, 1763, 1690, 1514, 1495, 1453; Enantiomeric ratio was measured by chiral phase HPLC (AD-H, 10% i-PrOH/Hexanes, 1.0 mL/min, 210 nm), Rt (major) = 18.07 Min, Rt (minor) = 15.16 Min; e.r. = 94:6.

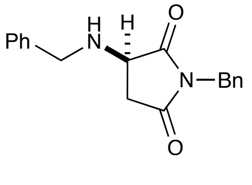

(R)-1-benzyl-3-(benzylamino)pyrrolidine-2,5-dione (7): Prepared according to the general procedure using benzylamine (0.024 g, 0.22 mmol, 1.1 equiv) to afford 0.049 g (84% yield) of product as a clear crystalline solid.

Analytical data for **7**: ^{1}H NMR (500 MHz, Chloroform-d) δ 7.32 (m, 10H), 4.65 (d, *J* = 2.4 Hz, 2H), 3.86 (q, *J* = 13.1 Hz, 2H), 3.75 (dd, *J* = 8.2, 5.0 Hz, 1H), 2.87 (dd, *J* = 17.9, 8.3 Hz, 1H), 2.52 (dd, *J* = 17.9, 5.0 Hz, 1H), 2.21 (s, 1H).^{13}C NMR (126 MHz, Chloroform-d) δ 177.6, 174.8, 138.6, 135.5, 128.8, 128.7 (x 2), 128.3, 128.0, 127.6, 55.5, 51.8, 42.4, 36.4. HRMS (ESI): Mass calculated for $C_{18}H_{19}N_2O_2$ [M+H]: 295.1447; found: 295.1445; IR (thin film) 3330, 3028, 2926, 2848, 1694, 1605, 1401, 1426, 1458, 1495, Enantiomeric ratio was measured by chiral phase HPLC (AD-H, 10% i-PrOH/Hexanes, 1.0 mL/min, 210 nm), Rt (major) = 18.14 Min, Rt (minor) = 14.78 Min; e.r. = 94:6.

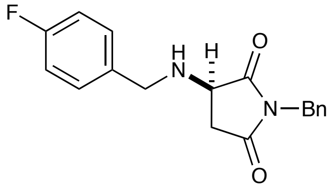

(R)-1-benzyl-3-((4-fluorobenzyl)amino)pyrrolidine-2,5-dione (8): Prepared according to the general procedure using (4-fluorophenyl)methanamine (0.028 g, 0.22 mmol, 1.1 equiv) to afford 0.048 g (77% yield) of product as a clear crystalline solid.

Analytical data for **8**: ^{1}H NMR (500 MHz, Chloroform-d) δ 7.32 (m, 7H), 7.01 (m, 2H), 4.65 (d, *J* = 2.0 Hz, 2H), 3.83 (s, 2H), 3.75 (dd, *J* = 8.3, 5.0 Hz, 1H), 2.88 (dd, *J* = 17.9, 8.3 Hz, 1H), 2.50 (dd, *J* = 17.9, 5.0 Hz, 1H), 2.14 (s, 1H). ^{13}C NMR (126 MHz, Chloroform-d) δ 177.5, 174.7, 162.2 (d, $J(^{13}C–^{19}F)$ = 245.7 Hz), 135.5, 134.5 (d, $J(^{13}C–^{19}F)$ = 3.2 Hz), 129.9 (d, $J(^{13}C–^{19}F)$ = 8.1 Hz), 128.8, 128.7, 128.1, 115.5 (d, $J(^{13}C–^{19}F)$ = 21.4 Hz), 55.6, 51.1, 42.5, 36.4; HRMS (ESI): Mass calculated for $C_{18}H_{18}FN_2O_2$ [M+H]: 313.1352; found: 313.1347; IR (thin film) 3300, 3035, 2845, 2912, 1598, 1507, 1482, 1446, 1429, 1401, 1357. Enantiomeric ratio was measured by chiral phase HPLC (AD-H, 10% i-PrOH/Hexanes, 1.0 mL/min, 210 nm), Rt (major) = 20.17 Min, Rt (minor) = 15.66 Min; e.r. = 93:7.

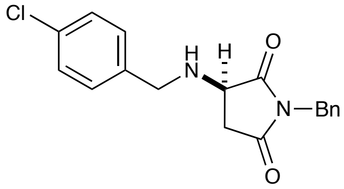

(R)-1-benzyl-3-((4-fluorobenzyl)amino)pyrrolidine-2,5-dione (9): Prepared according to the general procedure using (4-chlorophenyl)methanamine (0.031 g, 0.22 mmol, 1.1 equiv) to afford 0.060 g (91% yield) of product as a clear crystalline solid.

Analytical data for **9**: ^{1}H NMR (500 MHz, Chloroform-d) δ 7.28 (m, 9H), 4.62 (d, *J* = 1.9 Hz, 2H), 3.80 (s, 2H), 3.71 (dd, *J* = 8.3, 5.0 Hz, 1H), 2.84 (dd, *J* = 17.9, 8.3 Hz, 1H), 2.46 (dd, *J* = 17.9, 5.0 Hz, 1H), 2.11 (s, 1H). ^{13}C NMR (126 MHz, Chloroform-d) δ 177.5, 174.7, 137.2, 135.4, 133.3, 129.6, 128.8, 128.8, 128.7, 128.1, 55.5, 51.1, 42.5, 36.4 HRMS (ESI): Mass calculated for $C_{18}H_{18}ClN_2O_2$ [M+H]: 329.1057; found: 329.1051; IR (thin film): 3302, 3036, 2911, 2833, 1690, 1445, 1429, 1400, 1335. Enantiomeric ratio

was measured by chiral phase HPLC (AD-H, 10% i-PrOH/Hexanes, 1.0 mL/min, 210 nm), Rt (major) = 22.05 Min, Rt (minor) = 18.0 Min; e.r. = 93:7.

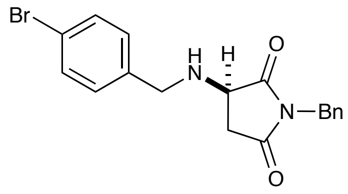

(R)-1-benzyl-3-((4-bromobenzyl)amino)pyrrolidine-2,5-dione (10): Prepared according to the general procedure using (4-bromophenyl)methanamine (0.041 g, 0.22 mmol, 1.1 equiv) to afford 0.067 g (90% yield) of product as a clear crystalline solid.

Analytical data for **10**: ^{1}H NMR (500 MHz, Chloroform-d) δ 7.45 (m, 2H), 7.37 (dd, *J* = 7.9, 1.7 Hz, 2H), 7.30 (m, 3H), 7.18 (d, *J* = 8.1 Hz, 2H), 4.65 (d, *J* = 2.1 Hz, 2H), 3.82 (s, 2H), 3.74 (dd, *J* = 8.3, 5.0 Hz, 1H), 2.87 (dd, *J* = 18.0, 8.3 Hz, 1H), 2.49 (dd, *J* = 18.0, 5.0 Hz, 1H), 2.13 (s, 1H).^{13}C NMR (126 MHz, Chloroform-d) δ 177.5, 174.6, 137.7, 135.4, 131.7, 129.9, 128.8, 128.7, 128.1, 121.4, 55.5, 51.2, 42.5, 36.4. HRMS (ESI): Mass calculated for $C_{18}H_{18}BrN_2O_2$ [M+H]: 373.0552; found: 373.0546; IR (thin film) 3300, 3010, 2833, 1690, 1487, 1466, 1454, 14229, 1402, 1361. Enantiomeric ratio was measured by chiral phase HPLC (AD-H, 10% i-PrOH/Hexanes, 1.0 mL/min, 210 nm), Rt (major) = 22.34 Min, Rt (minor) = 18.30 Min; e.r. = 93:7.

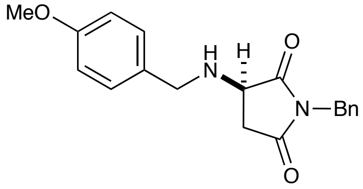

(R)-1-benzyl-3-((4-methoxybenzyl)amino)pyrrolidine-2,5-dione (11): Prepared according to the general procedure using (4-methoxyphenyl)methanamine (0.030 g, 0.22 mmol, 1.1 equiv) to afford 0.058 g (90% yield) of product as a clear crystalline solid.

Analytical data for **11**: ^{1}H NMR (500 MHz, Chloroform-d) δ 7.36 (m, 2H), 7.30 (m, 4H), 7.21 (d, *J* = 8.5 Hz, 2H), 6.86 (d, *J* = 8.6 Hz, 2H), 4.65 (d, *J* = 2.4 Hz, 2H), 3.79 (m, 6H), 2.85 (dd, *J* = 17.9, 8.3 Hz, 1H), 2.50 (dd, *J* = 18.0, 5.0 Hz, 1H);^{13}C NMR (126 MHz, Chloroform-d) δ 177.7, 174.9, 159.0, 135.5, 130.7, 129.5, 128.8, 128.7, 128.0, 114.0, 55.4, 55.3, 51.3, 42.4, 36.4; HRMS (ESI): Mass calculated for $C_{19}H_{20}N_2O_3Na$ [M+Na]: 347.1372; found: 347.1366; IR (thin film) 3301, 3050, 2836, 2957, 1689, 1607, 1401, 1428, 1445, 1482, 1513, 1581. Enantiomeric ratio was measured by chiral phase HPLC (AD-H, 10% i-PrOH/Hexanes, 1.0 mL/min, 210 nm), Rt (major) = 26.30 Min, Rt (minor) = 20.72 Min; e.r. = 94:6.

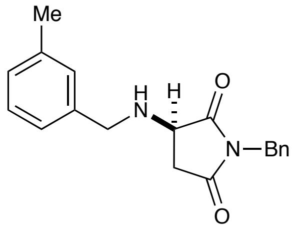

(R)-1-benzyl-3-((3-methylbenzyl)amino)pyrrolidine-2,5-dione (12): Prepared according to the general procedure using (3-methylphenyl)methanamine (0.027 g, 0.22 mmol, 1.1 equiv) to afford 0.059 g (96% yield) of product as a clear crystalline solid.

Analytical data for **12**: ^{1}H NMR (500 MHz, Chloroform-d) δ 7.37 (m, 2H), 7.30 (m, 3H), 7.22 (t, *J* = 7.5 Hz, 1H), 7.09 (m, 3H), 4.65 (d, *J* = 2.4 Hz, 2H), 3.77 (m, 3H), 2.87 (dd, *J* = 18.0, 8.2 Hz, 1H), 2.52 (dd, *J* = 17.9, 5.0 Hz, 1H), 2.34 (s, 3H), 2.21 (s, 1H). ^{13}C NMR (126 MHz, Chloroform-d) δ 177.6, 174.9, 138.5, 138.4, 135.5, 129.0, 128.8, 128.7, 128.5, 128.3, 128.0, 125.3, 55.5, 51.8, 42.4, 36.4, 21.4. HRMS (ESI): Mass calculated for $C_{19}H_{21}N_2O_2$ [M+H]: 309.1603; found: 309.1598; IR (thin film) 3289, 3009, 2810, 1689, 1607, 1432, 1453, 1496, 1513; Enantiomeric ratio was measured by chiral phase HPLC (AD-H, 10% i-PrOH/Hexanes, 1.0 mL/min, 210 nm), Rt (major) = 14.20 Min, Rt (minor) = 11.72 Min; e.r. = 95:5.

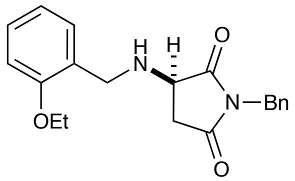

(R)-1-benzyl-3-((2-ethoxybenzyl)amino)pyrrolidine-2,5-dione (13): Prepared according to the general procedure using (2-ethoxyphenyl)methanamine (0.033 g, 0.22 mmol, 1.1 equiv) to afford 0.062 g (92% yield) of product as a clear crystalline solid.

Analytical data for **13**: ^{1}H NMR (500 MHz, Chloroform-d) δ 7.35 (m, 2H), 7.26 (m, 4H), 7.17 (dd, *J* = 7.4, 1.7 Hz, 1H), 6.88 (m, 2H), 4.64 (m, 2H), 4.08 (q, *J* = 7.0 Hz, 2H), 3.95 (d, *J* = 13.5 Hz, 1H), 3.73 (d, *J* = 13.5 Hz, 1H), 3.66 (dd, *J* = 8.1, 4.8 Hz, 1H), 2.88 (dd, *J* = 17.8, 8.1 Hz, 1H), 2.58 (m, 2H), 1.45 (t, *J* = 6.9 Hz, 3H).^{13}C NMR (126 MHz, Chloroform-d) δ 177.7, 175.2, 157.2, 135.5, 130.2, 128.9, 128.7, 128.6, 127.9, 126.5, 120.4, 111.3, 63.5, 54.8, 47.4, 42.4, 36.3, 14.9. HRMS (ESI): Mass calculated for $C_{20}H_{23}N_2O_3$ [M+H]: 339.1709; found: 339.1906; IR (thin film) 2933, 1699, 1430, 1354, 1476, 1493, 1587, 1599. Enantiomeric ratio was measured by chiral phase HPLC (AD-H, 10% i-PrOH/Hexanes, 0.5 mL/min, 210 nm), Rt (major) = 31.75 Min, Rt (minor) = 33.25 Min; e.r. = 97:3.

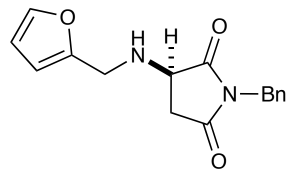

(R)-1-benzyl-3-((furan-2-ylmethyl)amino)pyrrolidine-2,5-dione (14): Prepared according to the general procedure using furan-2-ylmethanamine (0.021 g, 0.22 mmol, 1.1 equiv) to afford 0.028 g (49% yield) of product as a clear oil.

Analytical data for **14**: ^{1}H NMR (500 MHz, Chloroform-d) δ 7.22 (m, 6H), 6.16 (m, 2H), 4.53 (d, J = 1.9 Hz, 2H), 3.77 (m, 2H), 3.62 (dd, J = 8.2, 5.0 Hz, 1H), 2.76 (dd, J = 17.9, 8.2 Hz, 1H), 2.36 (dd, J = 18.0, 5.1 Hz, 1H), 2.23 (m, 1H). ^{13}C NMR (126 MHz, Chloroform-d) δ 177.3, 174.7, 152.1, 142.4, 135.4, 128.8, 128.6, 128.0, 110.3, 108.0, 55.0, 44.1, 42.4, 36.2; HRMS (ESI): Mass calculated for $C_{16}H_{17}N_2O_3$ [M+H]: 285.1239; found: 285.1232; IR (thin film) 3291, 3010, 2982, 2850, 1690, 1604, 1505, 1494, 1456, 1432; Enantiomeric ratio was measured by chiral phase HPLC (AD-H, 10% i-PrOH/Hexanes, 1.0 mL/min, 210 nm), Rt (major) = 19.49 Min, Rt (minor) = 17.13 Min; e.r. = 88:12.

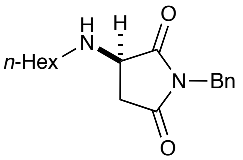

(R)-1-benzyl-3-(hexylamino)pyrrolidine-2,5-dione (15): Prepared according to the general procedure using *n*-hexylamine (0.022 g, 0.22 mmol, 1.1 equiv) to afford 0.036 g (63% yield) of product as a clear oil.

Analytical data for **15**: ^{1}H NMR (500 MHz, Chloroform-d) δ 7.32 (m, 5H), 4.65 (s, 2H), 3.75 (dd, J = 8.3, 4.9 Hz, 1H), 2.92 (dd, J = 18.0, 8.3 Hz, 1H), 2.65 (dt, J = 11.1, 7.1 Hz, 1H), 2.54 (m, 2H), 1.83 (s, 1H), 1.47 (q, J = 7.3 Hz, 2H), 1.29 (m, 7H), 0.88 (t, J = 6.8 Hz, 3H). ^{13}C NMR (126 MHz, $CDCl_3$) δ = 177.7, 175.0, 135.5, 128.8, 128.7, 128.0, 56.4, 47.7, 42.4, 36.3, 31.6, 29.9, 26.8, 22.5, 14.0. HRMS (ESI): Mass calculated for $C_{17}H_{25}N_2O_2$ [M+H]: 289.1916; found: 289.1911; IR (thin film) 3298, 3031, 2952, 2928, 2849, 1694, 1494, 1465, 1455, 1430; Enantiomeric ratio was measured by chiral phase HPLC (AD-H, 10% i-PrOH/Hexanes, 1.0 mL/min, 210 nm), Rt (major) = 11.37 Min, Rt (minor) = 9.12 Min; e.r. = 92:8.

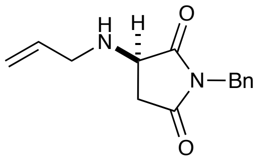

(R)-1-benzyl-3-(propylamino)pyrrolidine-2,5-dione (16): Prepared according to the general procedure using allylamine (0.013 g, 0.22 mmol, 1.1 equiv) to afford 0.048 g (97% yield) of product as a clear solid.

Analytical data for **16**: ^{1}H NMR (500 MHz, Chloroform-d) δ 7.37 (d, *J* = 6.8 Hz, 2H), 7.30 (m, 3H), 5.85 (ddt, *J* = 16.5, 10.3, 6.1 Hz, 1H), 5.18 (m, 2H), 4.65 (s, 2H), 3.79 (dd, *J* = 8.3, 5.0 Hz, 1H), 3.30 (m, 2H), 2.91 (dd, *J* = 17.9, 8.3 Hz, 1H), 2.53 (dd, *J* = 18.0, 5.0 Hz, 1H), 1.94 (s, 1H). ^{13}C NMR (126 MHz, Chloroform-d) ^{13}C NMR, δ 177.6, 174.8, 135.5, 135.4, 128.8, 128.7, 128.0, 117.3, 55.5, 50.3, 42.4, 36.5. HRMS (ESI): Mass calculated for $C_{14}H_{17}N_2O_2$ [M+H]: 245.1290; found: 245.1285; IR (thin film): 3301, 3035, 2928, 2855, 1690, 1497, 1455, 1430, 1397; Enantiomeric ratio was measured by chiral phase HPLC (AD-H, 10% i-PrOH/Hexanes, 1.0 mL/min, 210 nm), Rt (major) = 13.87 Min, Rt (minor) = 11.63 Min; e.r. = 91:9

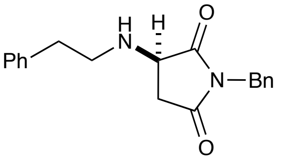

(R)-1-benzyl-3-(phenethylamino)pyrrolidine-2,5-dione (17): Prepared according to the general procedure using phenethylamine (0.027 g, 0.22 mmol, 1.1 equiv) to afford 0.062 g (81% yield) of product as a clear solid.

Analytical data for **17**: ^{1}H NMR (500 MHz, Chloroform-d) δ 7.33 (m, 7H), 7.21 (m, 3H), 4.64 (d, *J* = 1.6 Hz, 2H), 3.75 (dd, *J* = 8.3, 4.9 Hz, 1H), 2.88 (m, 5H), 2.50 (dd, *J* = 18.0, 4.9 Hz, 1H), 1.85 (s, 1H). ^{13}C NMR (126 MHz, Chloroform-d) δ 177.4, 174.8, 139.1, 135.5, 128.8, 128.7, 128.6, 128.6, 128.0, 126.5, 56.3, 48.8, 42.4, 36.2, 36.2; HRMS (ESI): Mass calculated for $C_{19}H_{21}N_2O_2$ [M+H]: 309.1603; found: 309.1598; IR (thin film) 330, 3028, 2918, 2860, 1693, 1604, 1497, 1465, 1453, 1431. Enantiomeric ratio was measured by chiral phase HPLC (AD-H, 10% i-PrOH/Hexanes, 1.0 mL/min, 210 nm), Rt (major) = 17.06 Min, Rt (minor) = 14.05 Min; e.r. = 88:12.

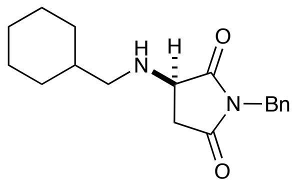

(R)-1-benzyl-3-((cyclohexylmethyl)amino)pyrrolidine-2,5-dione (18): Prepared according to the general procedure using cyclohexylmethanamine (0.025 g, 0.22 mmol, 1.1 equiv) to afford 0.058 g (97% yield) of product as a clear solid.

Analytical data for **18**: ^{1}H NMR (500 MHz, Chloroform-d) δ 7.33 (m, 5H), 4.65 (d, *J* = 1.9 Hz, 2H), 3.73 (dd, *J* = 8.3, 4.9 Hz, 1H), 2.91 (dd, *J* = 18.0, 8.3 Hz, 1H), 2.50 (dt, *J* = 17.8, 5.5 Hz, 2H), 2.37 (dd, *J* = 11.3, 6.7 Hz, 1H), 1.83 (s, 1H), 1.71 (m, 5H), 1.41 (m, 1H), 1.20 (m, 3H), 0.90 (m, 2H). ^{13}C NMR (126 MHz, $CDCl_3$) δ = 177.7, 175.0, 135.5, 128.8, 128.6, 128.0, 56.5, 54.2, 42.4, 38.0, 36.3, 31.2, 26.5, 25.9. Mass calculated for $C_{18}H_{25}N_2O_2$ [M+H]: 301.1916; found: 301.1911; IR (thin film) 3303, 2916, 2850, 1694, 1494, 1461, 1431; Enantiomeric ratio was measured by chiral phase HPLC (AD-H, 10% i-PrOH/Hexanes, 1.0 mL/min, 210 nm), Rt (major) = 9.75 Min, Rt (minor) = 9.18 Min; e.r. = 97:3.

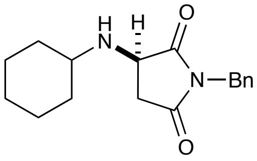

(R)-1-benzyl-3-(cyclohexylamino)pyrrolidine-2,5-dione (19): Prepared according to the general procedure using cyclohexylamine (0.022 g, 0.22 mmol, 1.1 equiv) to afford 0.048 g (84% yield) of product as a clear solid.

Analytical data for **19**: ^{1}H NMR (500 MHz, Chloroform-d) δ 7.32 (m, 5H), 4.65 (s, 2H), 3.86 (dd, *J* = 8.2, 5.0 Hz, 1H), 2.93 (dd, *J* = 17.9, 8.2 Hz, 1H), 2.54 (m, 2H), 1.86 (m, 2H), 1.60 (m, 3H), 1.18 (m, 5H). ^{13}C NMR (126 MHz, $CDCl_3$) δ 178.2, 175.0, 135.5, 128.9, 128.7, 128.0, 55.7, 54.2, 42.5, 38.1, 34.1, 33.1, 25.8, 24.9, 24.9. HRMS (ESI): Mass calculated for $C_{17}H_{23}N_2O_2$ [M+H]: 287.1760; found: 287.1754; IR (thin film) 3520, 3248, 3035, 2920, 2849, 1688, 1630, 1520, 1498, 1470, 1455, 1439, 1401; Enantiomeric ratio was measured by chiral phase supercritical fluid HPLC (IA, 2% MeOH/CO_2, 1.0 mL/min, 210 nm), Rt (major) = 2.16 Min, Rt (minor) = 2.38 Min; e.r. = 95:5.

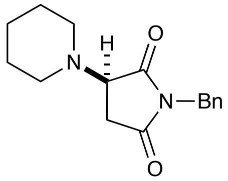

(R)-1-benzyl-3-(piperidin-1-yl)pyrrolidine-2,5-dione (20): Prepared according to the general procedure using piperidine (0.019 g, 0.22 mmol, 1.1 equiv) to afford 0.052 g (95% yield) of product as a clear oil.

Analytical data for **20**: ^{1}H NMR (500 MHz, Chloroform-d) δ 7.32 (m, 5H), 4.64 (m, 2H), 3.77 (dd, *J* = 9.1, 4.7 Hz, 1H), 2.82 (dd, *J* = 18.6, 9.0 Hz, 1H), 2.69 (m, 3H), 2.41 (dt, *J* = 11.0, 5.3 Hz, 2H), 1.59 (m, 4H), 1.43 (p, *J* = 6.0 Hz, 2H). ^{13}C NMR (126 MHz, $CDCl_3$) δ 176.2, 175.0, 135.8, 128.8, 128.6, 127.9, 63.2, 50.2, 42.1, 31.6, 26.0, 24.0. HRMS (ESI): Mass calculated for $C_{16}H_{21}N_2O_2$ [M+H]: 273.1603; found: 273.1598; IR (thin film) 2934, 2846, 1691, 1499, 1470, 1454, 1441, 1422; Enantiomeric ratio was measured by chiral phase HPLC (AD-H, 10% i-PrOH/Hexanes, 1.0 mL/min, 210 nm), Rt (major) = 23.70 Min, Rt (minor) = 27.27 Min; e.r. = 97:3.

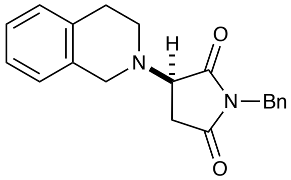

(R)-1-benzyl-3-(3,4-dihydroisoquinolin-2(1H)-yl)pyrrolidine-2,5-dione (21): Prepared according to the general procedure using 1,2,3,4-tetrahydroisoquinoline (0.029 g, 0.22 mmol, 1.1 equiv) to afford 0.045 g (71% yield) of product as a clear oil.

Analytical data for **21**: ^{1}H NMR (500 MHz, Chloroform-d) δ 7.40 (m, 2H), 7.31 (m, 3H), 7.11 (m, 3H), 6.95 (m, 1H), 4.69 (m, 2H), 4.05 (d, *J* = 14.3 Hz, 1H), 3.97 (dd, *J* = 9.0, 4.7 Hz, 1H), 3.70 (d, *J* = 14.3 Hz, 1H), 2.87 (m, 6H). ^{13}C NMR (126 MHz, $CDCl_3$) δ ^{13}C NMR, δ 175.9, 174.7, 135.7, 133.7, 133.6, 128.9, 128.8, 128.7, 128.0, 126.5, 126.3, 125.8, 62.2, 51.9, 47.0, 42.3, 32.3, 29.4. HRMS (ESI): Mass calculated for $C_{20}H_{21}N_2O_2$ [M+H]: 321.1603; found: 321.1598; IR (thin film) 3282, 3004, 2922, 1692, 1605, 1585, 1498, 1455, 1424, 1400; Enantiomeric ratio was measured by chiral phase HPLC (AD-H, 10% i-PrOH/Hexanes, 1.0 mL/min, 210 nm), Rt (major) = 20.75 Min, Rt (minor) = 13.99 Min; e.r. = 93:7.

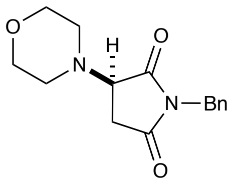

(R)-1-benzyl-3-morpholinopyrrolidine-2,5-dione (22): Prepared according to the general procedure using morpholine (0.019 g, 0.22 mmol, 1.1 equiv) to afford 0.051 g (93% yield) of product as a clear oil.

Analytical data for **22**: ^{1}H NMR (500 MHz, Chloroform-d) δ 7.28 (m, 5H), 4.60 (m, 2H), 3.66 (dt, *J* = 19.5, 4.4 Hz, 5H), 2.76 (m, 3H), 2.61 (dd, *J* = 18.4, 4.9 Hz, 1H), 2.43 (dt, *J* = 10.3, 4.6 Hz, 2H). ^{13}C NMR, δ 175.5, 174.5, 135.6, 128.8, 128.7, 128.0, 66.8, 62.5, 49.5, 42.2, 31.4. HRMS (ESI): Mass calculated for $C_{15}H_{19}N_2O_3$ [M+H]: 275.1396; found: 275.1390; IR (thin film) 2853, 1698, 1496, 1454, 1429; Enantiomeric ratio was measured by chiral phase HPLC (AD-H, 10% i-PrOH/Hexanes, 0.5 mL/min, 210 nm), Rt (major) = 51.83 Min, Rt (minor) = 49.28 Min; e.r. = 97:3.

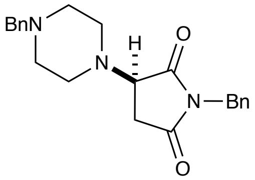

(R)-1-benzyl-3-(4-benzylpiperazin-1-yl)pyrrolidine-2,5-dione (23): Prepared according to the general procedure using 1-benzylpiperazine (0.039 g, 0.22 mmol, 1.1 equiv) to afford 0.068 g (93% yield) of product as a clear oil.

Analytical data for **23**: ^{1}H NMR (500 MHz, Chloroform-d) δ 7.32 (m, 10H), 4.65 (d, *J* = 5.3 Hz, 2H), 3.79 (dd, *J* = 8.8, 4.9 Hz, 1H), 3.51 (s, 2H), 2.80 (m, 3H), 2.67 (dd, *J* = 18.4, 5.0 Hz, 1H), 2.50 (s, 6H). ^{13}C NMR (126 MHz, $CDCl_3$) δ 175.8, 174.7, 137.9, 135.7, 129.1, 128.8, 128.6, 128.2, 128.0, 127.1, 62.8, 62.4, 52.8, 49.0, 42.2, 31.2. Mass calculated for $C_{22}H_{26}N_3O_2$ [M+H]: 364; found: 364. HRMS (ESI): Mass calculated for $C_{22}H_{26}N_3O_2$ [M+H]: 364.2025; found: 364.2020; IR (thin film) 2918, 2814, 1692, 1496, 1453, 1426, 1401; Enantiomeric ratio was measured by chiral phase HPLC (AD-H, 10% i-PrOH/Hexanes, 1.0 mL/min, 210 nm), Rt (major) = 22.91 Min, Rt (minor) = 15.41 Min; e.r. = 93:7.

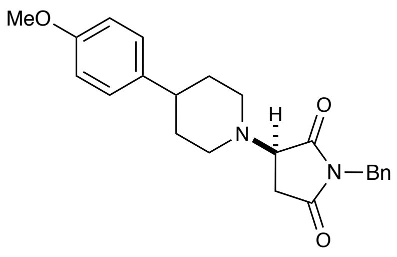

(R)-1-benzyl-3-(4-(4-methoxyphenyl)piperidin-1-yl)pyrrolidine-2,5-dione (24): Prepared according to the general procedure using 4-(4-methoxyphenyl)piperidine (0.042 g, 0.22 mmol, 1.1 equiv) to afford 0.070 g (92% yield) of product as a colorless solid. An improved procedure was developed by only changing the temperature to -40 °C to afford 0.068 g (90% yield) of product as a colorless solid.

Analytical data for **24**: ^{1}H NMR (500 MHz, Chloroform-d) δ 7.33 (m, 5H), 7.12 (m, 2H), 6.84 (m, 2H), 4.67 (m, 2H), 3.85 (ddd, *J* = 9.4, 4.8, 1.9 Hz, 1H), 3.78 (d, *J* = 1.9 Hz, 3H), 2.87 (td, *J* = 13.3, 11.2, 7.6 Hz, 4H), 2.69 (ddd, *J* = 18.5, 4.9, 1.8 Hz, 1H), 2.45 (m, 1H), 2.27 (td, *J* = 11.3, 2.7 Hz, 1H), 1.77 (m, 4H). ^{13}C NMR (126 MHz, $CDCl_3$) δ 176.1, 174.9, 158.0, 138.0, 135.7, 128.8, 128.7, 128.0, 127.6, 113.8, 62.8, 55.2, 51.5, 48.5, 42.2, 41.4, 33.8, 33.5, 31.7. HRMS (ESI): Mass calculated for $C_{23}H_{27}N_2O_3$ [M+H]: 379.2022; found: 379.2016. IR (thin film) 3291, 2848, 2914, 1691, 1597, 1514, 1491, 1454, 1435.
For the standard reaction run at –20 °C, enantiomeric ratio was measured by chiral phase HPLC (AD-H, 10% i-PrOH/Hexanes, 1.0 mL/min, 210 nm), Rt (major) = 31.84 Min, Rt (minor) = 17.77 Min; e.r. = 90:10. For the same reaction run at -40 °C, enantiomeric ratio was measured by chiral phase HPLC (AD-H, 10% i-PrOH/Hexanes, 1.0 mL/min, 210 nm), Rt (major) = 32.26 Min, Rt (minor) = 17.91 Min; e.r. = 94:6.

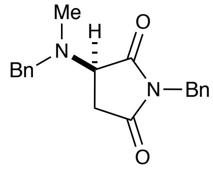

(R)-1-benzyl-3-(benzyl(methyl)amino)pyrrolidine-2,5-dione (25): Prepared according to the general procedure using *N*-methyl-phenethylamine (0.024 g, 0.22 mmol, 1.1 equiv) to afford 0.055 g (89% yield) of product as a clear oil.

Analytical data for **25**: ^{1}H NMR (500 MHz, Chloroform-d) δ 7.32 (m, 10H), 4.67 (m, 2H), 3.86 (m, 2H), 3.70 (d, *J* = 13.1 Hz, 1H), 2.80 (dd, *J* = 18.6, 9.1 Hz, 1H), 2.63 (dd, *J* = 18.6, 4.8 Hz, 1H), 2.25 (s, 3H). ^{13}C NMR (126 MHz, $CDCl_3$) δ 176.6, 174.9, 137.9, 135.8, 128.9, 128.8, 128.6, 128.5, 128.0, 127.5, 60.3, 59.1, 42.2, 37.2, 31.8. HRMS (ESI): Mass calculated for $C_{19}H_{21}N_2O_2$ [M+H]: 309.1603; found: 309.1598; IR (thin film) 3069, 2955, 1701, 1493, 1467, 1417; Enantiomeric ratio was measured by chiral phase HPLC (IA, 2% i-PrOH/Hexanes, 1.0 mL/min, 210 nm), Rt (major) = 19.75 Min, Rt (minor) = 20.98 Min; e.r. = 78:22.

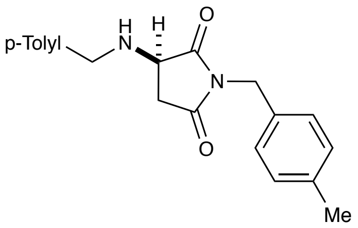

(R)-1-(4-methylbenzyl)-3-((4-methylbenzyl)amino)pyrrolidine-2,5-dione (26): Prepared according to the general procedure using 1-(4-methylbenzyl)-1H-pyrrole-2,5-dione (0.040 g, 0.20 mmol, 1.0 equiv) and *p*-tolylmethanamine (0.027 g, 0.22 mmol, 1.1 equiv) to afford 0.060 g (94% yield) of product as a clear solid.

Analytical data for **26**: ^{1}H NMR (500 MHz, Chloroform-d) δ 7.23 (s, 2H), 7.12 (m, 6H), 4.58 (d, J = 2.9 Hz, 2H), 3.75 (m, 3H), 2.81 (dd, J = 17.9, 8.2 Hz, 1H), 2.47 (dd, J = 17.9, 5.0 Hz, 1H), 2.30 (d, J = 11.1 Hz, 6H), 2.13 (s, 1H). ^{13}C NMR (126 MHz, Chloroform-d) δ 177.7, 174.9, 137.8, 137.2, 135.6, 132.6, 129.3, 129.3, 128.8, 128.2, 55.4, 51.6, 42.2, 36.5, 21.1, 21.1. HRMS (ESI): Mass calculated for $C_{20}H_{23}N_2O_2$ [M+H]: 323.1760; found: 323.1554; IR (thin film) 3289, 2980, 2918, 2852, 1691, 1615, 1514, 1454, 1437; Enantiomeric ratio was measured by chiral phase HPLC (AD-H, 10% i-PrOH/Hexanes, 1.0 mL/min, 210 nm), Rt (major) = 16.75 Min, Rt (minor) = 13.13 Min; e.r. = 92:8.

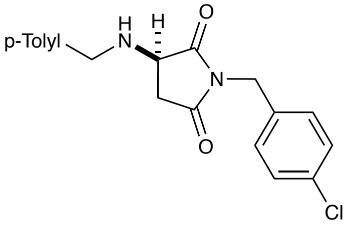

(R)-1-(4-chlorobenzyl)-3-((4-methylbenzyl)amino)pyrrolidine-2,5-dione (27): Prepared according to the general procedure using 1-(4-chlorobenzyl)-1H-pyrrole-2,5-dione (0.044 g, 0.20 mmol, 1.0 equiv) and *p*-tolylmethanamine (0.027 g, 0.22 mmol, 1.1 equiv) to afford 0.066 g (97% yield) of product as a clear solid.

Analytical data for **27**: ^{1}H NMR (500 MHz, Chloroform-d) δ 7.30 (m, 4H), 7.16 (m, 4H), 4.60 (d, J = 1.9 Hz, 2H), 3.78 (m, 3H), 2.85 (dd, J = 18.0, 8.2 Hz, 1H), 2.50 (dd, J = 18.0, 4.9 Hz, 1H), 2.33 (s, 3H), 2.15 (s, 1H). ^{13}C NMR (126 MHz, Chloroform-d) δ 177.5, 174.8, 137.3, 135.5, 134.0, 133.9, 130.3, 129.3, 128.8, 128.2, 55.4, 51.6, 41.7, 36.4, 21.1. HRMS (ESI): Mass calculated for $C_{19}H_{20}ClN_2O_2$ [M+H]: 343.1213; found: 343.1208; IR (thin film) 3291, 2917, 2847, 1691, 1597, 1514, 1491, 1454, 1435; Enantiomeric ratio was measured by chiral phase HPLC (AD-H, 10% i-PrOH/Hexanes, 1.0 mL/min, 210 nm), Rt (major) = 17.92 Min, Rt (minor) = 15.52 Min; e.r. = 94:6.

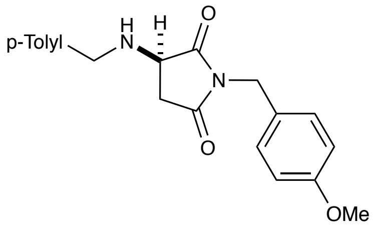

(R)-1-(4-methoxybenzyl)-3-((4-methylbenzyl)amino)pyrrolidine-2,5-dione (28): Prepared according to the general procedure using 1-(4-methoxybenzyl)-1H-pyrrole-2,5-dione (0.040 g, 0.20 mmol, 1.0 equiv) and *p*-tolylmethanamine (0.027 g, 0.22 mmol, 1.1 equiv) to afford 0.062 g (91% yield) of product as a clear solid.

Analytical data for **28**: ^{1}H NMR (500 MHz, Chloroform-d) δ 7.31 (m, 2H), 7.15 (m, 4H), 6.82 (m, 2H), 4.58 (d, J = 2.8 Hz, 2H), 3.78 (m, 5H), 3.71 (dd, J = 8.3, 4.9 Hz, 1H), 2.83 (dd, J = 17.9, 8.3 Hz, 1H), 2.48 (dd, J = 17.9, 5.0 Hz, 1H), 2.33 (s, 3H), 2.15 (s, 1H). ^{13}C NMR (126 MHz, Chloroform-d) δ 177.7, 174.9, 159.3, 137.2, 135.6, 130.3, 129.3, 128.2, 127.8, 114.0, 55.4, 55.3, 51.6, 41.9, 36.4, 21.1. HRMS (ESI): Mass calculated for $C_{20}H_{23}N_2O_3$ [M+H]: 339.1709; found: 339.1703; IR (thin film): 3284, 3018, 2913, 2857, 1687, 1614, 1585, 1513, 1437, 1398, 1353; Enantiomeric ratio was measured by chiral phase HPLC (AD-H, 10% i-PrOH/Hexanes, 1.0 mL/min, 210 nm), Rt (major) = 31.8 Min, Rt (minor) = 23.20 Min; e.r. = 87:13.

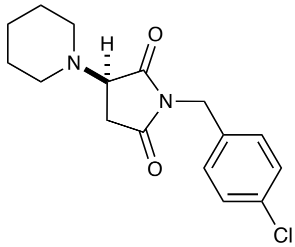

(R)-1-(4-chlorobenzyl)-3-(piperidin-1-yl)pyrrolidine-2,5-dione (29): Prepared according to the general procedure using 1-(4-chlorobenzyl)-1H-pyrrole-2,5-dione (0.044 g, 0.20 mmol, 1.0 equiv) and piperidine (0.019 g, 0.22, 1.1 equiv) to afford 0.055 g (89% yield) of product as a clear solid.

Analytical data for **29**: ^{1}H NMR (500 MHz, Chloroform-d) δ 7.30 (m, 4H), 4.62 (m, 2H), 3.77 (dd, J = 9.1, 4.7 Hz, 1H), 2.82 (dd, J = 18.6, 9.0 Hz, 1H), 2.67 (m, 3H), 2.40 (dt, J = 10.8, 5.3 Hz, 2H), 1.55 (d, J = 5.5 Hz, 4H), 1.44 (m, 2H). ^{13}C NMR (126 MHz, Chloroform-d) δ 176.1, 174.9, 134.2, 133.9, 130.3, 128.8, 63.2, 50.2, 41.4, 31.6, 26.0, 24.0. HRMS (ESI): Mass calculated for $C_{16}H_{20}ClN_2O_2$ [M+H]: 307.1213; found: 323.1208; IR (thin film) 2927, 2822, 1688, 1508, 1449, 1424, 1395. For the standard reaction run at -20 °C, enantiomeric ratio was measured by chiral phase HPLC (AD-H, 10% i-PrOH/Hexanes, 1.0 mL/min, 210 nm), Rt (major) = 10.68 Min, Rt (minor) = 13.16 Min; e.r. = 93:7. For the same reaction run at -40 °C, enantiomeric ratio was measured by chiral phase HPLC (AD-H, 10% i-PrOH/Hexanes, 1.0 mL/min, 210 nm), Rt (major) = 10.64 Min, Rt (minor) = 13.13 Min; e.r. = 84:16.

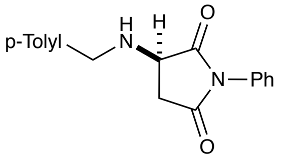

(R)-3-((4-methylbenzyl)amino)-1-phenylpyrrolidine-2,5-dione (30): Prepared according to the general procedure using *p*-tolylmethanamine (0.027 g, 0.22 mmol, 1.1 equiv) and 1-phenyl-1H-pyrrole-2,5-dione (0.035 g, 0.2 mmol, 1.0 equiv) to afford 0.059 g (93% yield) of **30** as a white solid.

Analytical data for **30**: ^{1}H NMR (500 MHz, Chloroform-d) δ 7.50 (dd, *J* = 8.4, 7.0 Hz, 2H), 7.43 (m, 1H), 7.29 (m, 4H), 7.20 (d, *J* = 7.7 Hz, 2H), 3.95 (m, 3H), 3.05 (dd, *J* = 18.0, 8.4 Hz, 1H), 2.73 (dd, *J* = 18.0, 5.3 Hz, 1H), 2.38 (s, 4H). ^{13}C NMR (126 MHz, $CDCl_3$) δ 176.8, 174.1, 137.4, 135.3, 131.6, 129.4, 129.2, 128.7, 128.4, 126.3, 55.4, 51.6, 36.5, 21.1. HRMS (ESI): Mass calculated for $C_{18}H_{19}N_2O_2$ [M+H]: 295.1447; found: 295.1441; IR (thin film): 3299, 3047, 1698, 1594, 1513,1495, 1453, 1395, 1370. Enantiomeric ratio was measured by chiral phase HPLC (IA, 30% i-PrOH/Hexanes, 0.5 mL/min, 210 nm), Rt (major) = 28.33 Min, Rt (minor) = 27.03 Min; e.r. = 74:26

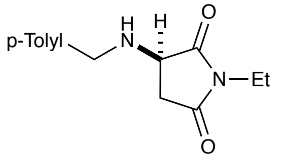

(R)-1-ethyl-3-((4-methylbenzyl)amino)pyrrolidine-2,5-dione (31): Prepared according to the general procedure using *p*-tolylmethanamine (0.027 g, 0.22 mmol, 1.1 equiv) and 1-ethyl-1H-pyrrole-2,5-dione (0.025 g, 0.20 mmol, 1.0 equiv) to afford 0.045 g (91% yield) of product **31** as a clear oil.

Analytical data for **31**: ^{1}H NMR (500 MHz, Chloroform-d) δ 7.17 (m, 4H), 3.81 (m, 2H), 3.72 (dd, *J* = 8.2, 4.9 Hz, 1H), 3.55 (q, *J* = 7.2 Hz, 2H), 2.83 (dd, *J* = 17.9, 8.2 Hz, 1H), 2.49 (dd, *J* = 17.9, 4.9 Hz, 1H), 2.34 (s, 3H), 1.16 (t, *J* = 7.2 Hz, 3H). ^{13}C NMR (126 MHz, $CDCl_3$) δ 177.8, 175.2, 137.3, 135.5, 129.3, 128.2, 55.4, 51.7, 36.5, 33.8, 21.1, 13.0. HRMS (ESI): Mass calculated for $C_{14}H_{19}N_2O_2$ [M+H]: 247.1447; found: 247.1441; IR (thin film) 3294, 2981, 2848, 1686, 1516, 1491, 1447, 1405; Enantiomeric ratio was measured by chiral phase HPLC (AD-H, 10% i-PrOH/Hexanes, 1.0 mL/min, 210 nm), Rt (major) = 12.56 Min, Rt (minor) = 11.21 Min; e.r. = 80:20.

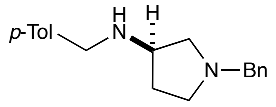

(R)-1-benzyl-N-(4-methylbenzyl)pyrrolidin-3-amine (34): A flame-dried 25 mL round bottom flask equipped with a magnetic stirring bar and a nitrogen inlet was charged with THF (7.6 mL) and lithium aluminum hydride (0.215 g, 5.67 mmol, 5.0 equiv). To the resulting suspension was added 1-benzyl-3-((4-methylbenzyl)amino)pyrrolidine-2,5-dione **6** (0.350 g, 1.135 mmol) portion-wise as a solid. The reaction flask was equipped with a reflux condenser and heated to 60 °C for 14 h. The reaction mixture was then cooled to 23 ºC and poured into ice cold 1.0 M NaOH (50 mL) and stirred for 10 min. The mixture was transferred to a separatory funnel and extracted with diethyl ether (3 x 100 mL). The organic layer was collected, dried over sodium sulfate, filtered and concentrated on a rotary evaporator to give the product **34** as analytically pure pale yellow oil (0.305 g, 1.08 mmol, 95%).

Analytical data for **34**: ^{1}H NMR (500 MHz, Chloroform-d) δ 7.31 (dt, *J* = 8.6, 4.1 Hz, 4H), 7.25 (m, 2H), 7.19 (m, 2H), 7.11 (m, 2H), 3.69 (d, *J* = 1.8 Hz, 2H), 3.60 (qd, *J* = 12.9, 1.8 Hz, 2H), 3.34 (ddtd, *J* = 8.8, 6.7, 4.8, 1.7 Hz, 1H), 2.75 (ddd, *J* = 8.8, 6.7, 1.7 Hz, 1H), 2.63 (tdd, *J* = 8.4, 6.0, 1.8 Hz, 1H), 2.53 (tdd, *J* = 9.4, 6.7, 1.7 Hz, 1H), 2.39 (ddd, *J* = 9.5, 5.0, 1.8 Hz, 1H), 2.33 (d, *J* = 1.7 Hz, 3H), 2.13 (m, 1H), 1.61 (dddt, *J* = 12.8, 10.9, 6.1, 3.0 Hz, 1H), 1.37 (s, 1H). ^{13}C NMR (126 MHz, $CDCl_3$) δ 139.1, 137.3, 136.4, 129.0, 128.8, 128.2, 128.1, 126.9, 60.8, 60.5, 56.7, 53.1, 52.1, 32.2, 21.1. HRMS (ESI): Mass calculated for $C_{19}H_{25}N_2$ [M+H]: 281.2018; found: 281.2012; IR (thin film): 3025, 2955, 2910, 2783, 1514, 1494, 1452; Enantiomeric ratio was measured by chiral phase HPLC (OD-H, 5% i-PrOH/Hexanes, 1.0 mL/min, 210 nm), Rt (major) = 15.91 Min, Rt (minor) = 11.56 Min; e.r. = 99:1.

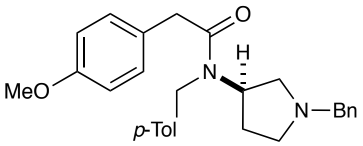

(R)-N-(1-benzylpyrrolidin-3-yl)-2-(4-methoxyphenyl)-N-(4-methylbenzyl)acetamide (35): To a 25 ml flask charged with dichloromethane (10.9 ml) was added sequentially (R)-1-benzyl-N-(4-methylbenzyl)pyrrolidin-3-amine **34** (0.305 g, 1.088 mmol), Hunig's Base (2.280 mL, 13.05 mmol, 12 equiv), and 2-(4-methoxyphenyl)acetyl chloride (0.602 g, 3.26 mmol, 3.0 equiv) at 23 °C. The reaction was allowed to stir at 23 °C for 48 h, at which point it was poured into a separatory funnel containing saturated $NaHCO_3$ (10 mL) and extracted with dichloromethane (3 x 15 mL). The combined organic layers were washed with water (15 mL), saturated brine (15 mL), then dried over Na_2SO_4, filtered, and concentrated on a rotary evaporator. The crude residue was purified via column chromatography (9:1 hexanes:acetone) to afford product **35** as a colorless oil (0.201 g, 0.469 mmol, 43%), which was characterized as a ~2:1 mixture of amide rotamers at 23 ºC by NMR.

Analytical data for **35**: ^{1}H NMR (500 MHz, Chloroform-d) δ 7.20 (m, 11H), 7.06 (q, *J* = 8.5, 7.4 Hz, 6H), 6.83 (dd, *J* = 20.1, 8.1 Hz, 3H), 5.17 (m, 1H), 4.65 (dt, *J* = 32.4, 18.0 Hz, 4H), 3.79 (d, *J* = 7.3 Hz, 6H), 3.54 (t, *J* = 14.2 Hz, 2H), 3.47 (m, 4H), 2.79 (t, *J* = 7.5 Hz, 1H), 2.59 (ddd, *J* = 26.5, 10.4, 4.0 Hz, 1H), 2.47 (t, *J* = 9.1 Hz, 1H), 2.30 (m, 8H), 1.90 (t, *J* = 8.8 Hz, 1H), 1.70 (dt, *J* = 18.2, 6.2 Hz, 2H). ^{13}C NMR (126 MHz, $CDCl_3$) δ 172.4, 171.4, 158.4, 138.9, 138.5, 136.7, 136.6, 136.0, 135.7, 129.7, 129.7, 129.5, 129.0, 128.6, 128.4, 128.3, 128.2, 127.3, 127.1, 127.0, 126.8, 126.8, 125.4, 114.1, 114.0, 60.0, 57.5, 57.4, 57.0, 56.0, 55.2, 53.6, 53.3, 53.3, 47.5, 45.5, 40.9, 40.6, 30.0, 29.9, 24.7, 21.0. HRMS (ESI): Mass calculated for $C_{28}H_{33}N_2O_2$ [M+H]: 429.2542; found: 429.2537; IR (thin film): 3334, 3055, 2898, 2875, 1716, 1652, 1558, 1512, 1456, 1419, 1379.

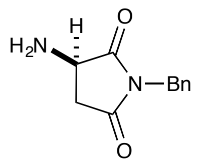

(R)-3-amino-1-benzylpyrrolidine-2,5-dione (36): To a flame dried 10 mL round bottom flask under an inert atmosphere was added 10% Pd/C (21.28 mg, 0.020 mmol), (R)-1-benzyl-3-((4-methylbenzyl)amino)pyrrolidine-2,5-dione **6** (61.7 mg, 0.2 mmol), followed by MeOH (4.0 ml). The flask was then equipped with a balloon of H_2 (1 atm) and stirred at 23 °C for 5 h, at which point the reaction mixture was filtered through a 0.5 cm plug of celite^{TM}. The filter cake was rinsed with 20 mL of ethyl acetate, and the resulting clear homogeneous filtrate was concentrated *in vacuo*. The resulting crude residue was purified on SiO_2 in the following manner: a chloroform solution (0.5 mL) of the crude residue was loaded onto a dry pad of silica gel (2 x 2 cm), which was first flushed with hexanes (15 mL) then with MeCN (~15 mL) until all of **36** had been eluted (TLC monitoring). The MeCN eluent was concentrated *in vacuo* to give **36** as a colorless solid (39 mg, 0.192 mmol, 96% yield).

Analytical data for **36**: ^{1}H NMR (500 MHz, Chloroform-d) δ 7.34 (m, 5H), 4.66 (s, 2H), 3.90 (dd, *J* = 8.7, 5.3 Hz, 1H), 3.05 (dd, *J* = 18.1, 8.7 Hz, 1H), 2.46 (dd, *J* = 18.1, 5.4 Hz, 1H) 1.68 (s, 2H). ^{13}C NMR (126 MHz, $CDCl_3$) δ 179.0, 174.5, 135.5, 128.9, 128.7, 128.1, 50.5, 42.5, 37.9. HRMS (ESI): Mass calculated for $C_{11}H_{13}N_2O_2$ [M+H]: 205.0977; found: 205.0972; IR (thin film): 3302, 3036, 2932, 2810, 1697, 1608, 1581, 1512, 1501, 1455, 1423, 1362. Enantiomeric ratio was measured by chiral phase HPLC (OD-H, 2% i-PrOH/Hexanes, 1.0 mL/min, 210 nm), Rt (major) = 52.71 Min, Rt (minor) = not observed Min; e.r. = >99:1.

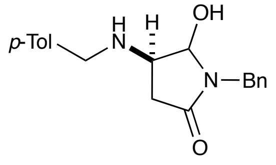

(R)-1-benzyl-4-((4-methylbenzyl)amino)-5-hydroxypyrrolidin-2-one (SI-1): To a flame dried 25 mL flask charged with 2:1 CH_2Cl_2:MeOH (2 mL) was added (R)-1-benzyl-3-((4-methylbenzyl)amino)pyrrolidine-2,5-dione **6** (61.7 mg, 0.2 mmol), and the

resulting solution was cooled to 0 °C using an external ice bath. Sodium borohydride (7.6 mg, 0.2 mmol, 1.0 equiv) was added in a single bolus, after which the reaction was warmed to 4 °C and stirred at this temperature for 14 h. The crude reaction was quenched with 5 mL saturated aqueous $NaHCO_3$, washed into a separatory funnel with 5 mL of EtOAc, and extracted with EtOAc (3 x 5 mL). The combined organic layers were washed with saturated aqueous brine (5 mL), dried over Na_2SO_4, filtered and concentrated on a rotary evaporator. The crude oily product was triturated with ethyl acetate, resulting in precipitation of a white solid. The precipitate was filtered, washed with cold ethyl acetate (0.5 mL) and dried under high-vacuum (~0.1 Torr) to give a white solid (0.026 g, 0.084 mmol, 42%) which was characterized as the *trans*-isomer of **SI-1**.

Analytical data for ***trans*-SI-1:** ^{1}H NMR (500 MHz, Chloroform-d) δ 7.32 (m, 6H), 7.13 (m, 4H), 4.83 (m, 2H), 4.25 (d, *J* = 14.9 Hz, 1H), 3.72 (m, 2H), 3.23 (m, 1H), 2.85 (dd, *J* = 17.1, 7.6 Hz, 1H), 2.33 (s, 3H), 2.22 (dd, *J* = 17.2, 4.1 Hz, 1H), 2.07 (s, 1H). ^{13}C NMR (126 MHz, $CDCl_3$), δ 172.6, 137.0, 136.3, 136.2, 129.2, 128.8, 128.3, 128.1, 127.7, 87.7, 59.7, 51.5, 43.6, 37.1, 21.1.

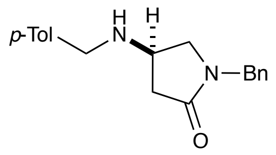

(R)-1-benzyl-4-((4-methylbenzyl)amino)pyrrolidin-2-one (37): To a 25 mL round bottom flask was added **SI-1** (49 mg, 0.158 mmol, 1.0 equiv), followed by dichloromethane (16 mL), and triethylsilane (0.252 mL, 1.58 mmol, 10.0 equiv). The resulting solution was cooled to -78 °C in an acetone/dry ice bath, whereupon BF_3•OEt_2 (0.050 mL, 0.395 mmol, 2.5 equiv) was added dropwise via syringe. The cooling bath was removed after 15 min, and the reaction was stirred at 23 °C for 16 h. The crude reaction was quenched by addition of 10 mL of saturated aqueous $NaHCO_3$, and then transferred to a separatory funnel. The mixture was extracted with dichloromethane (3 x 15 mL), and the combined organic portions were washed with brine (15 mL), collected and dried over sodium sulfate, filtered, and concentrated to a crude oily residue. The crude product was purified via flash column chromatography (Hexanes:Acetone, gradient 10:1 → 1:1) to yield **37** as a colorless oil (39 mg, 0.132 mmol, 84%).

Analytical data for **37**: ^{1}H NMR (500 MHz, Chloroform-d) δ 7.31 (m, 4H), 7.24 (m, 2H), 7.12 (m, 4H), 4.46 (d, *J* = 2.9 Hz, 2H), 3.69 (m, 2H), 3.48 (tt, *J* = 7.5, 5.0 Hz, 1H), 3.42 (dd, *J* = 9.8, 6.9 Hz, 1H), 3.07 (dd, *J* = 9.8, 4.4 Hz, 1H), 2.69 (dd, *J* = 16.9, 7.7 Hz, 1H), 2.34 (m, 4H). ^{13}C NMR (126 MHz, $CDCl_3$), δ 173.0, 136.9, 136.4, 136.3, 129.2, 128.7, 128.1, 128.0, 127.6, 53.0, 51.4, 50.3, 46.4, 38.9, 21.1. HRMS (ESI): Mass calculated for $C_{19}H_{23}N_2O$ [M+H]: 295.1810; found: 295.1805; IR (thin film): 3283, 3017, 2919, 2856, 1685, 1614, 1585, 1513, 1496, 1437, 1399, 1354. Enantiomeric ratio was measured by chiral phase HPLC (AD-H, 10% i-PrOH/Hexanes, 1.0 mL/min, 210 nm), Rt (major) = 18.35 Min, Rt (minor) = 20.53 Min; e.r. = 97:3.

References:

[1] Pangborn, A. B.; Giardello, M. A.; Grubbs, R. H.; Rosen, R. K.; Timmers F. J., *Organometallics* **1996**, *15*, 1518–1520.

[2] Perrin, D. D.; Armarego, W. L. *Purification of Laboratory Chemicals;* 3rd Ed., Pergamon Press, Oxford. **1988**.

[3] Isozaki, H.; Yasugi, M.; Takigawa, N.; Hotta, K.; Ichihara, E.; Taniguchi, A.; Toyooka, S.; Hashida, S.; Sendo, T.; Tanimoto, M.; Kiura, K.; *Jpn. J. Clin. Onc.* **2014**, 44, 963–968

[4] Katayama, R.; Khan, T.M.; Benes, C.; Lifshits, E.; Ebi, H.; Rivera, W.M.; Shakespeare, W.C.; Iafrate, A.J.; Engelmana, J.A.; Shaw, A.T., *Proc. Nat. Acad. Sci. U.S.A.* **2011**, 108, 7535.

[5] Katayama R.; Shaw A.T.; Khan T.M.; Mino-Kenudson, M.; Solomon,B.J.; Halmos, B.; Jessop, N.A.; Wain, J.C.; Yeo, A.T.; Benes, C.; Drew, L.; Saeh, J.C.; Crosby, K.; Sequist, L.V.; Iafrate, A.J.; Engelman, J.A., *Sci. Trans. Med*. **2012**, 4, 120.

X-ray Crystallography Data
Determination of the Absolute Configuration of 10

The absolute stereochemistry of **10** was determined by X-ray diffraction. **10** was recrystallized from ethyl acetate/hexane.

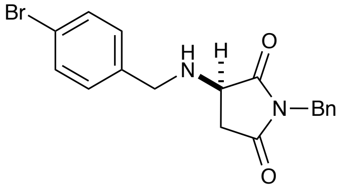

10

X-ray crystal structure of **10**:
ORTEP: C= gray, O= red, N= Blue, Br= maroon

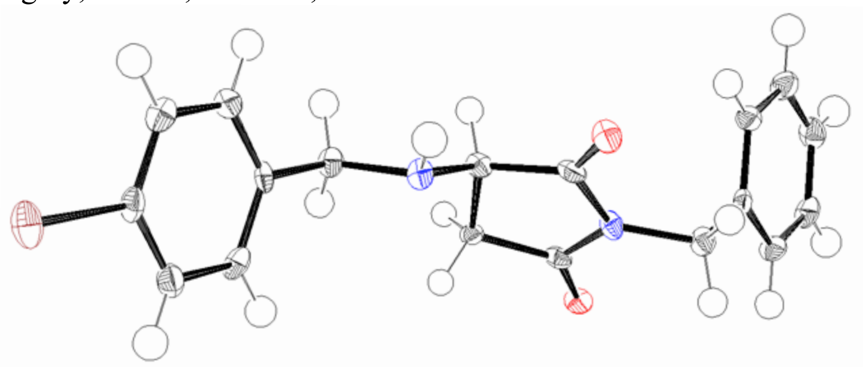

X-ray diffraction was performed at 100.01 K and raw frame data were processed using SAINT. Molecular structure was solved using direct methods and refined on F2 by full-matrix leastsquare techniques. The GOF = 1.111 for 212 variables refined to R1 = 0.0485 for 2336 reflections with I>2α(I). A multi-scan absorption correction was performed and the Flack parameter was -0.02(4). Further information can be found in the CIF file. This crystal was deposited in the Cambridge Crystallographic Data Centre and assigned as CCDC 1531303.

Determination of structure of Ca[B]$_2$

The absolute stereochemistry and structure of **Ca[B]$_2$** was determined by X-ray diffraction. **Ca[B]$_2$** was recrystallized from toluene.

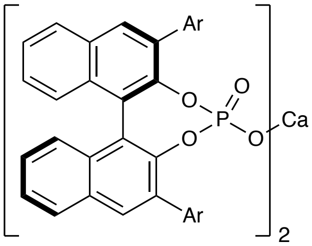

Ar = 9-phenanthracene

Ca[**B**]$_2$

X-ray crystal structure of **Ca[B]$_2$**:

ORTEP:
C= gray, O= red, P= orange, Ca= pink

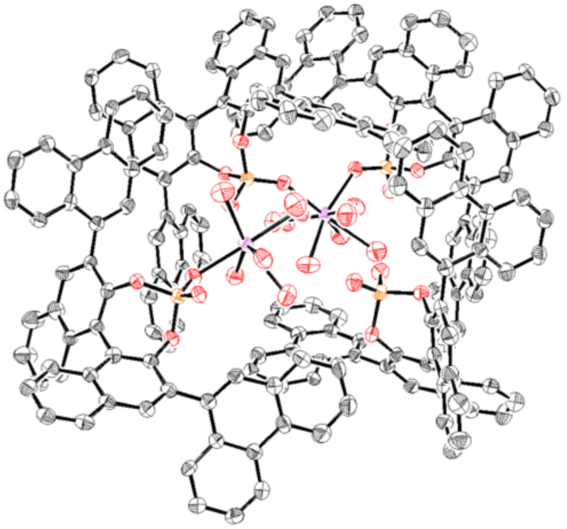

CYL View:
C= gray, O= red, P= orange, Ca= green

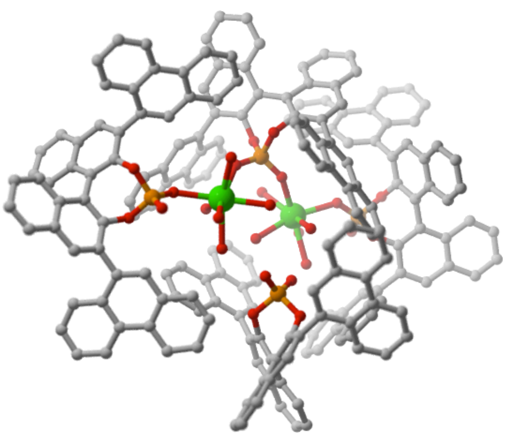

X-ray diffraction was performed at 99.99 K and raw frame data were processed using SAINT. Molecular structure was solved using the algorithm implemented in SHELXT and refined on F2 by full-matrix leastsquare techniques. The GOF = 0.928 for 2078 variables refined to R1 = 0.0630 for 20787 reflections with I>2α(I). A multi-scan absorption correction was performed and the Flack parameter was 0.026(10). Further information can be found in the CIF file. This crystal was deposited in the Cambridge Crystallographic Data Centre and assigned as CCDC 1531265.

Selected Spectra

^{1}H NMR spectrum of **Ca[B]$_2$** (500 MHz, $CDCl_3$)

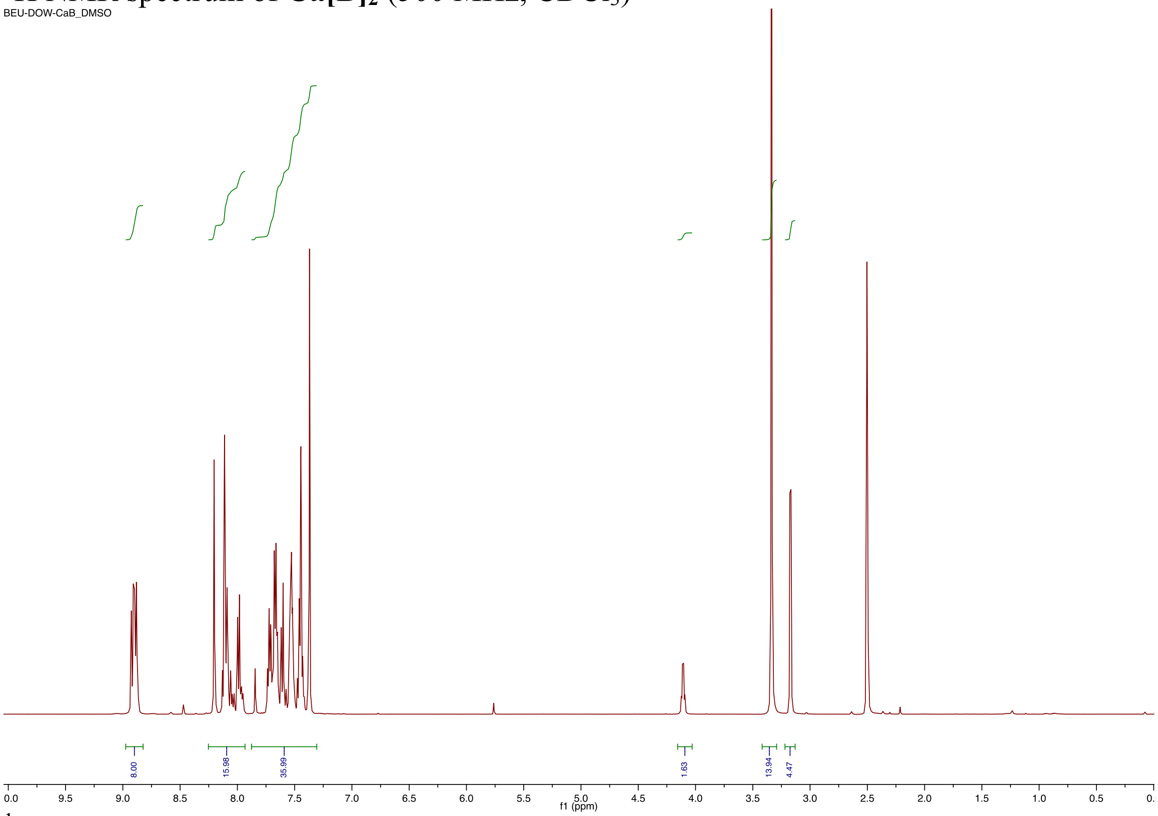

^{1}H NMR spectrum of **Ca[B]$_2$** (500 MHz, $CDCl_3$)

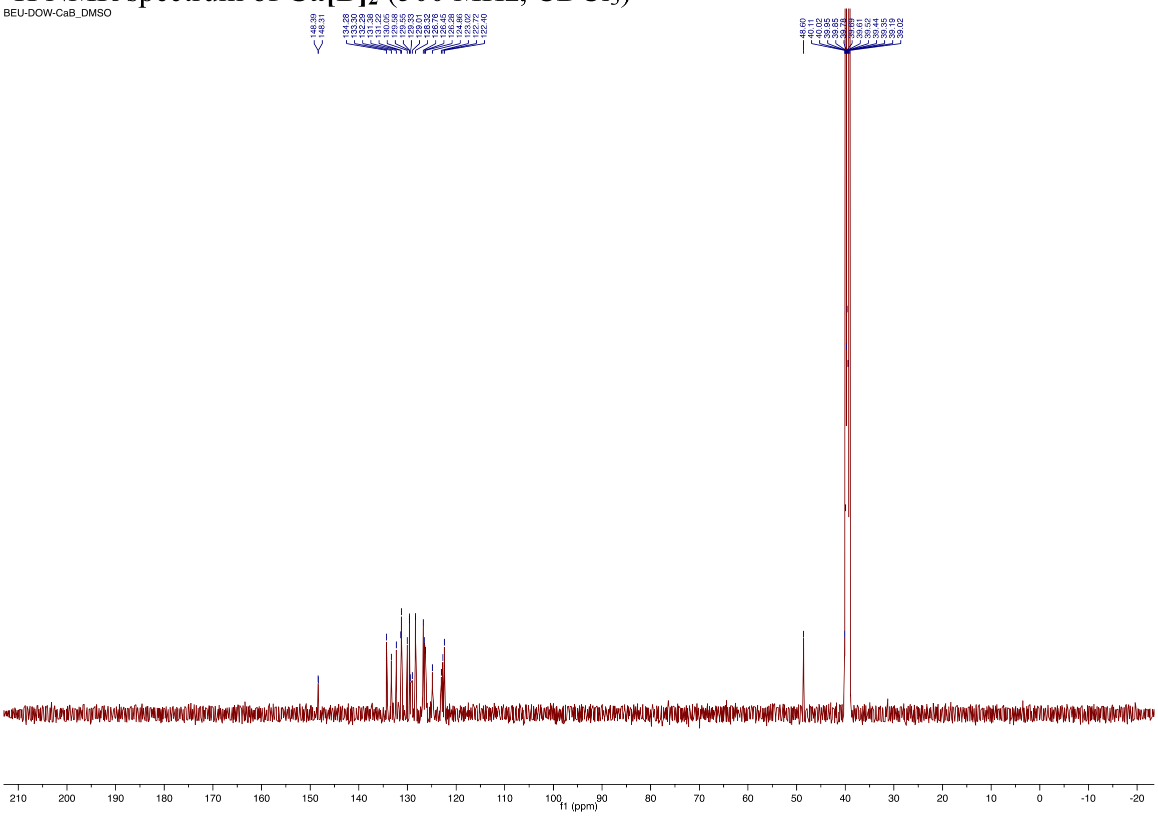

^{1}H NMR spectrum of **6** (500 MHz, $CDCl_3$)

BEU-DOW10-05

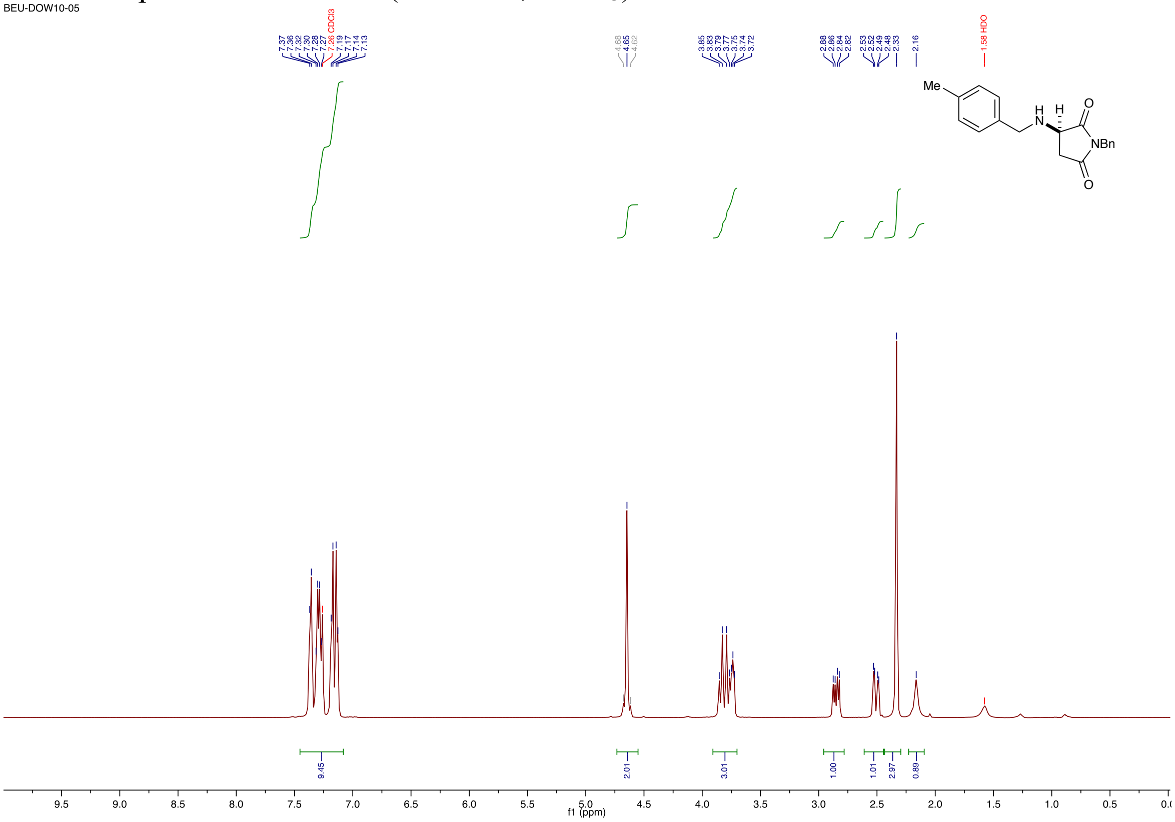

^{13}C NMR spectrum of **6** (126 MHz, $CDCl_3$)

BEU-DOW10-05

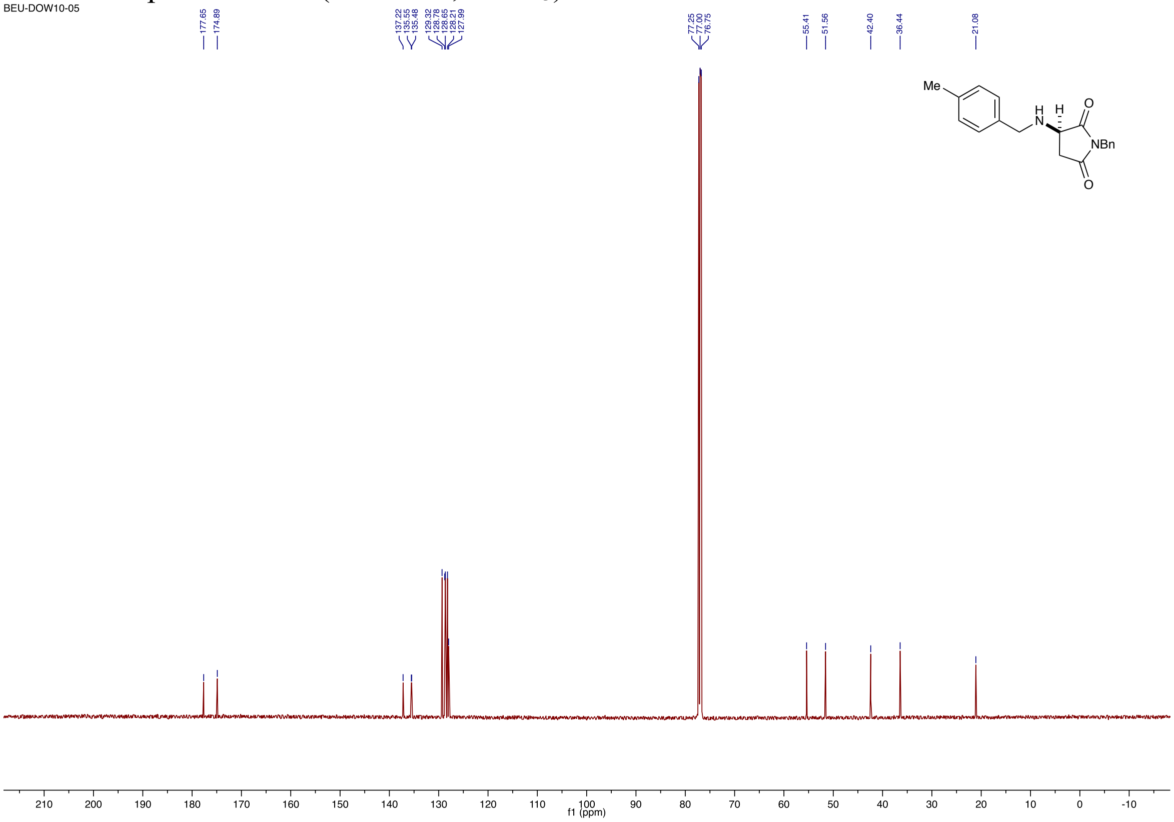

^{1}H NMR spectrum of **7** (500 MHz, $CDCl_3$)

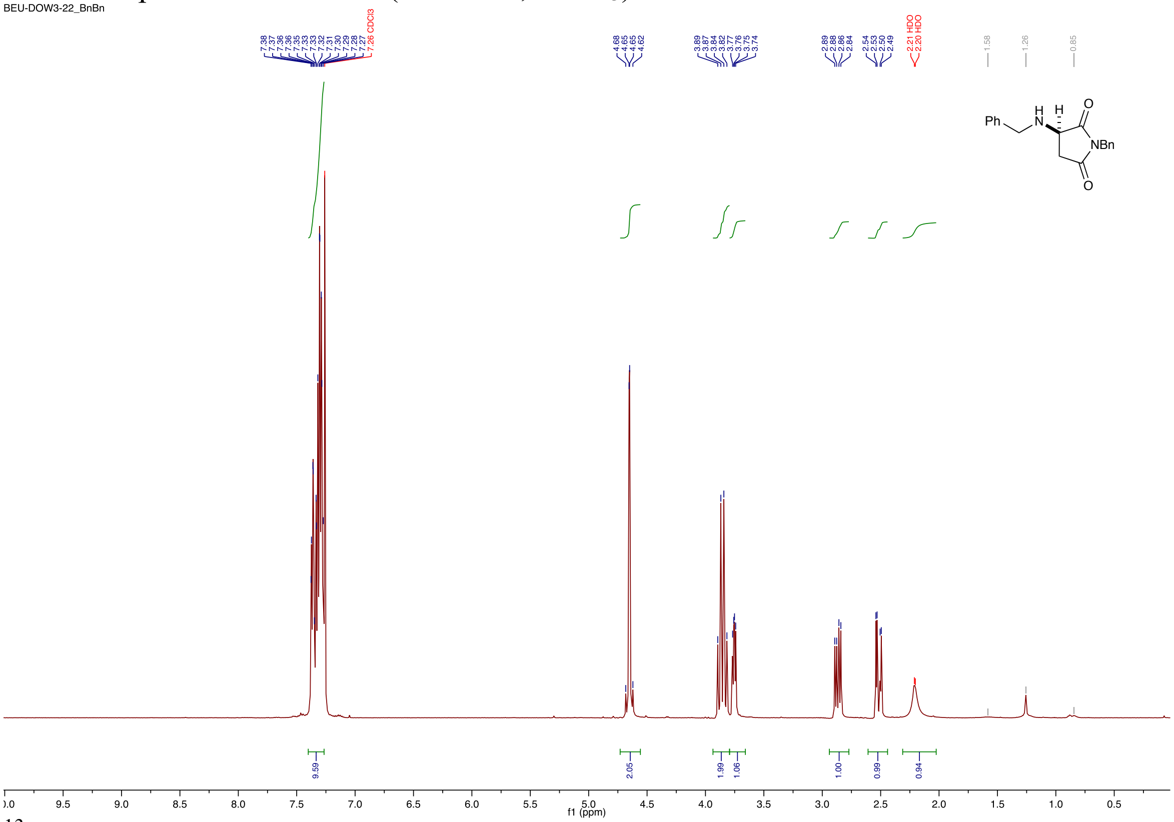

^{13}C NMR spectrum of **7** (126 MHz, $CDCl_3$)

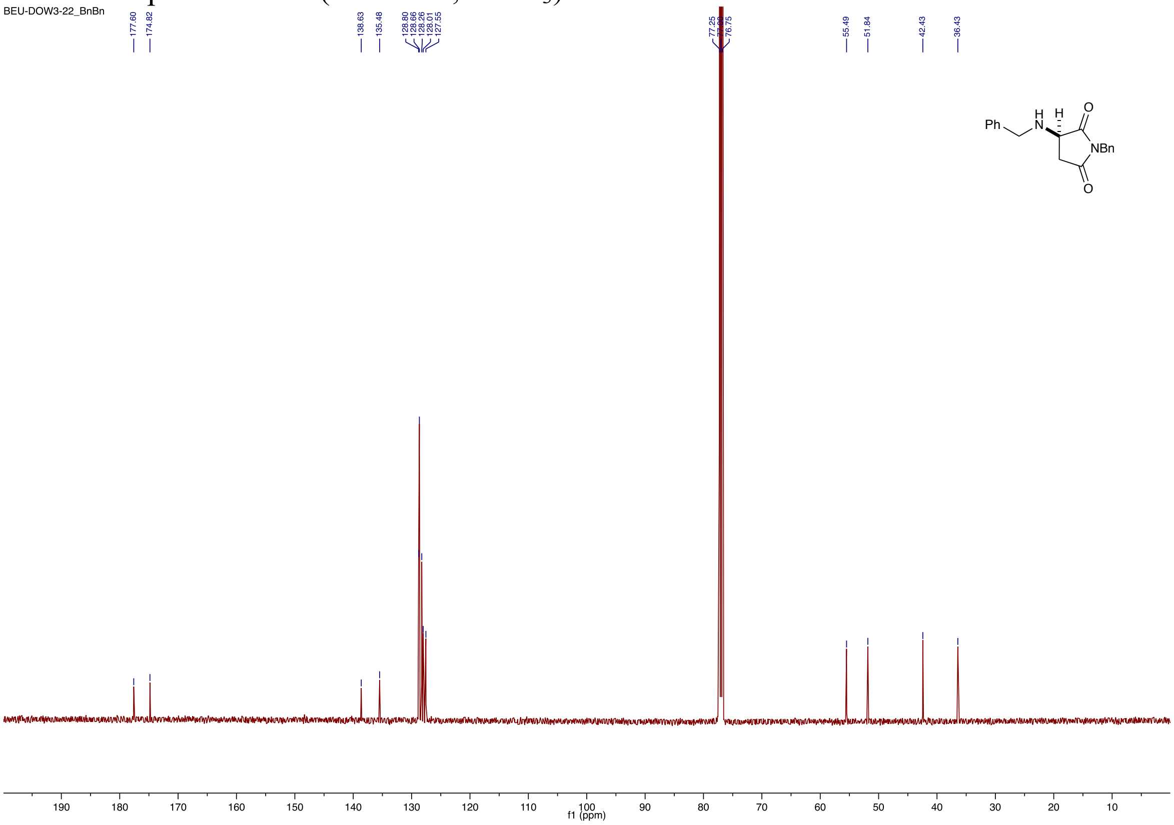

^{1}H NMR spectrum of **8** (500 MHz, $CDCl_3$)

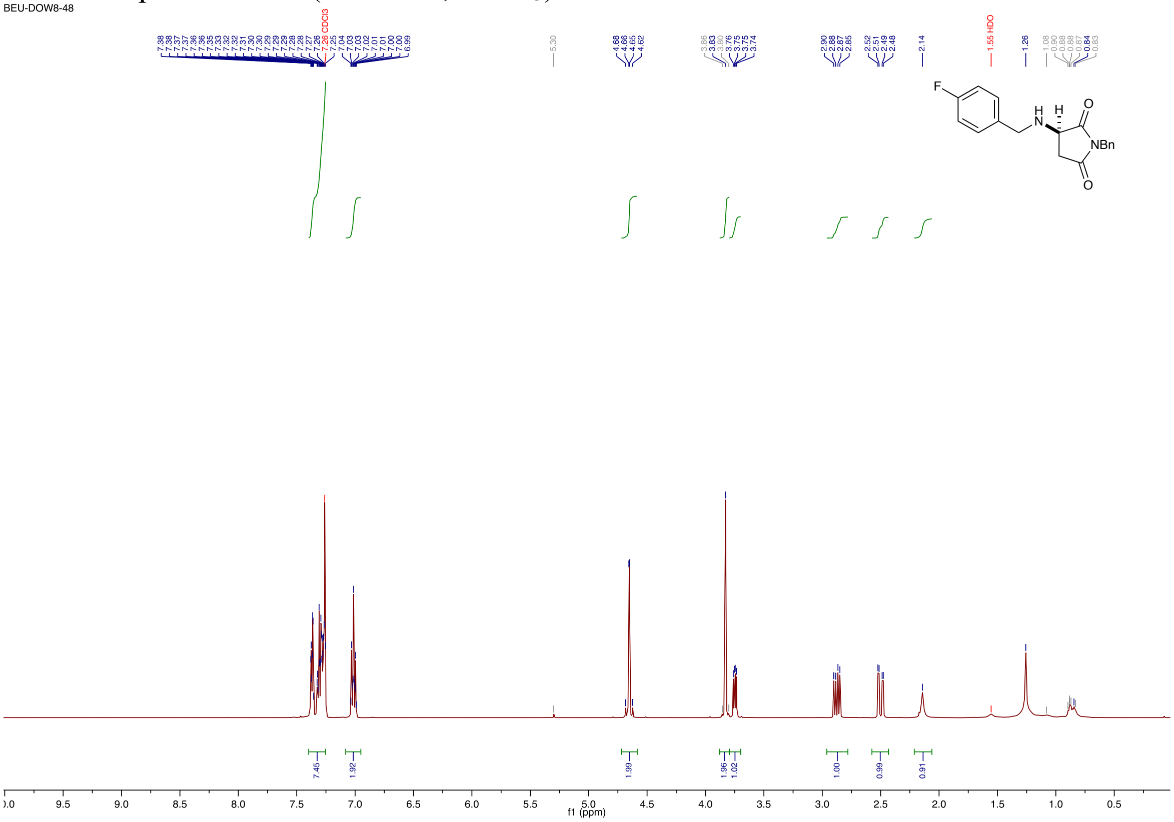

^{13}C NMR spectrum of **8** (126 MHz, $CDCl_3$)

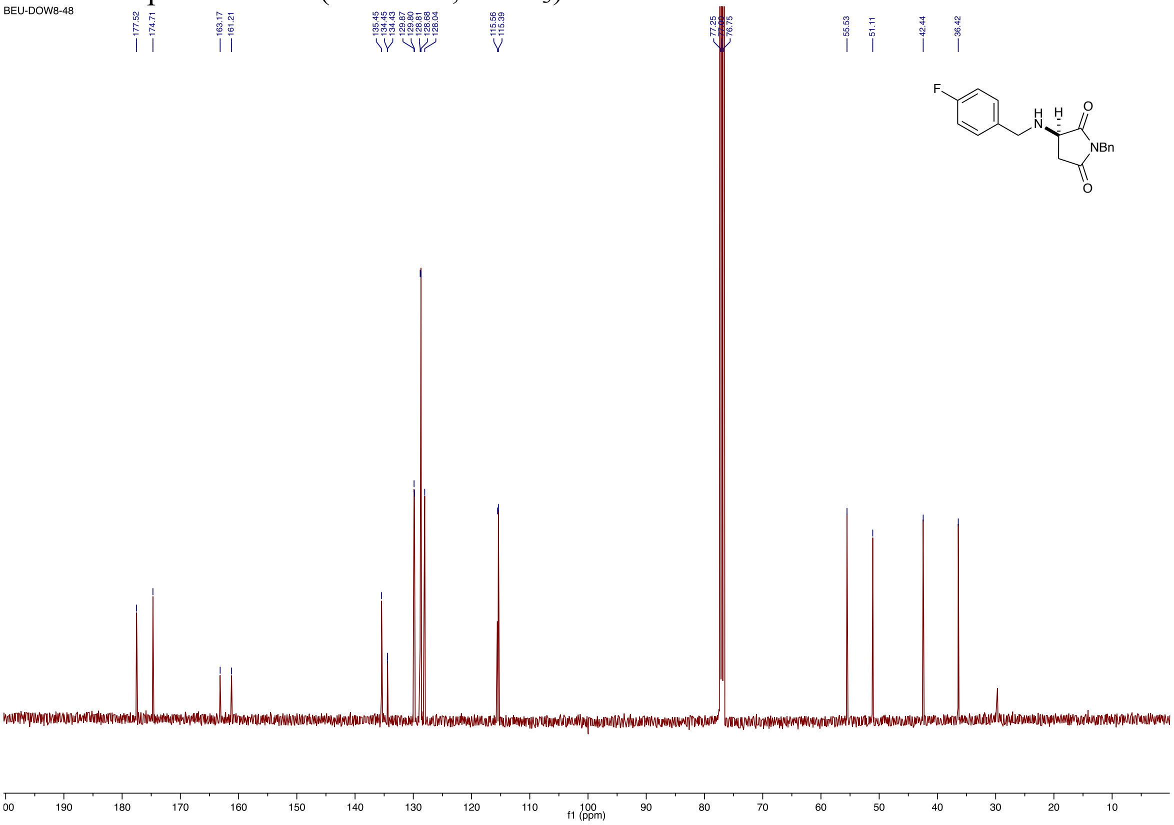

^{1}H NMR spectrum of **9** (500 MHz, $CDCl_3$)

BEU-DOW8-33

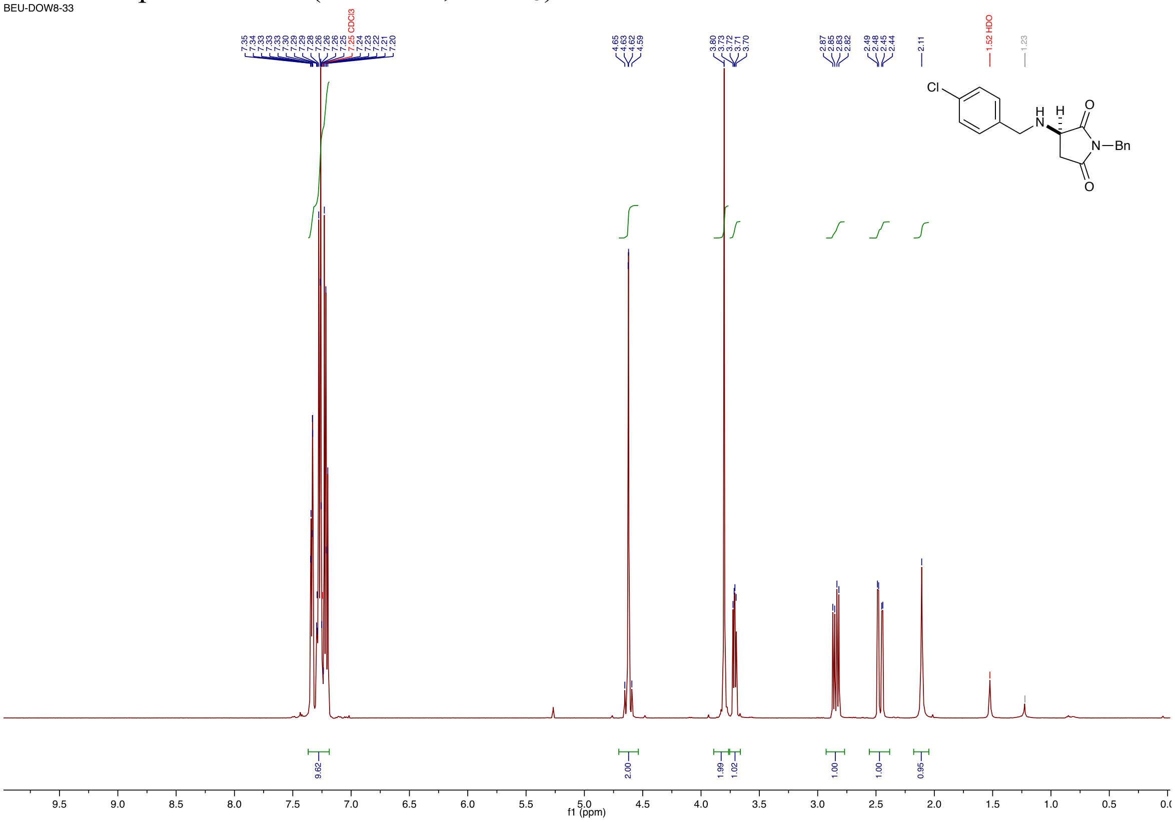

^{13}C NMR spectrum of **9** (126 MHz, $CDCl_3$)

BEU-DOW8-33

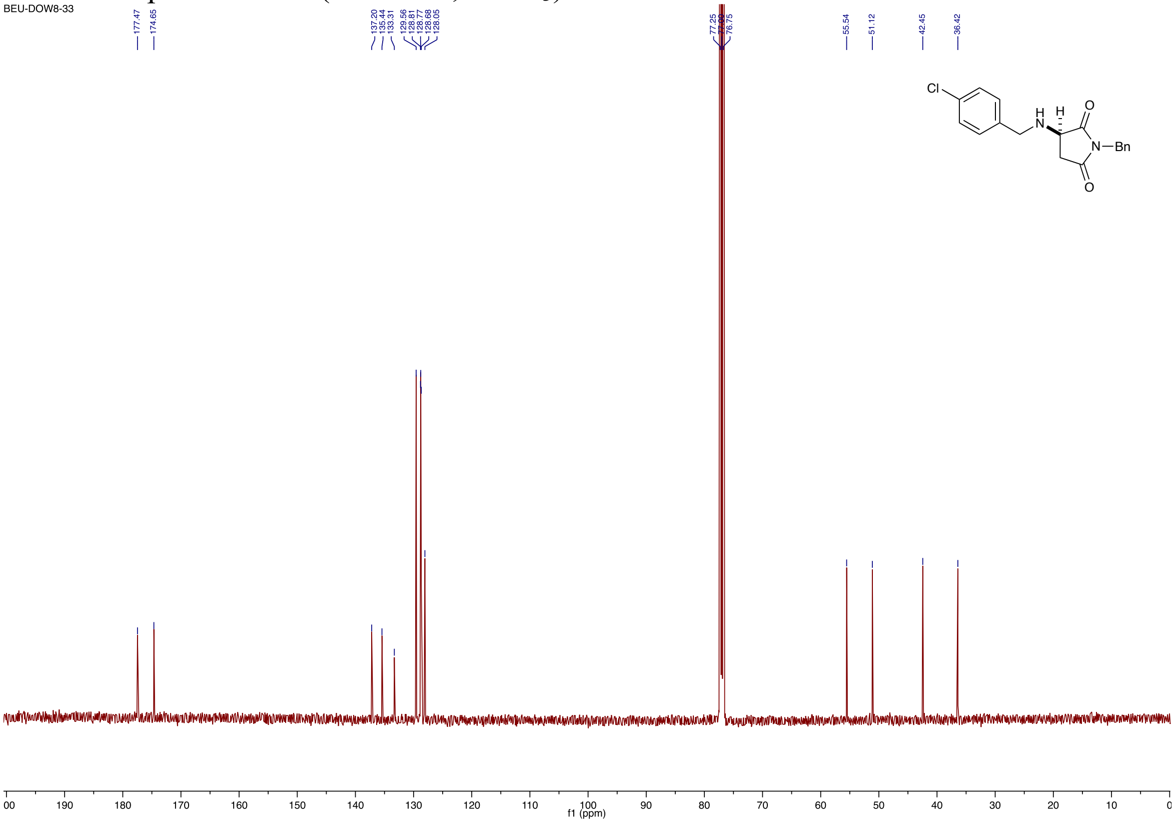

^{1}H NMR spectrum of **10** (500 MHz, $CDCl_3$)

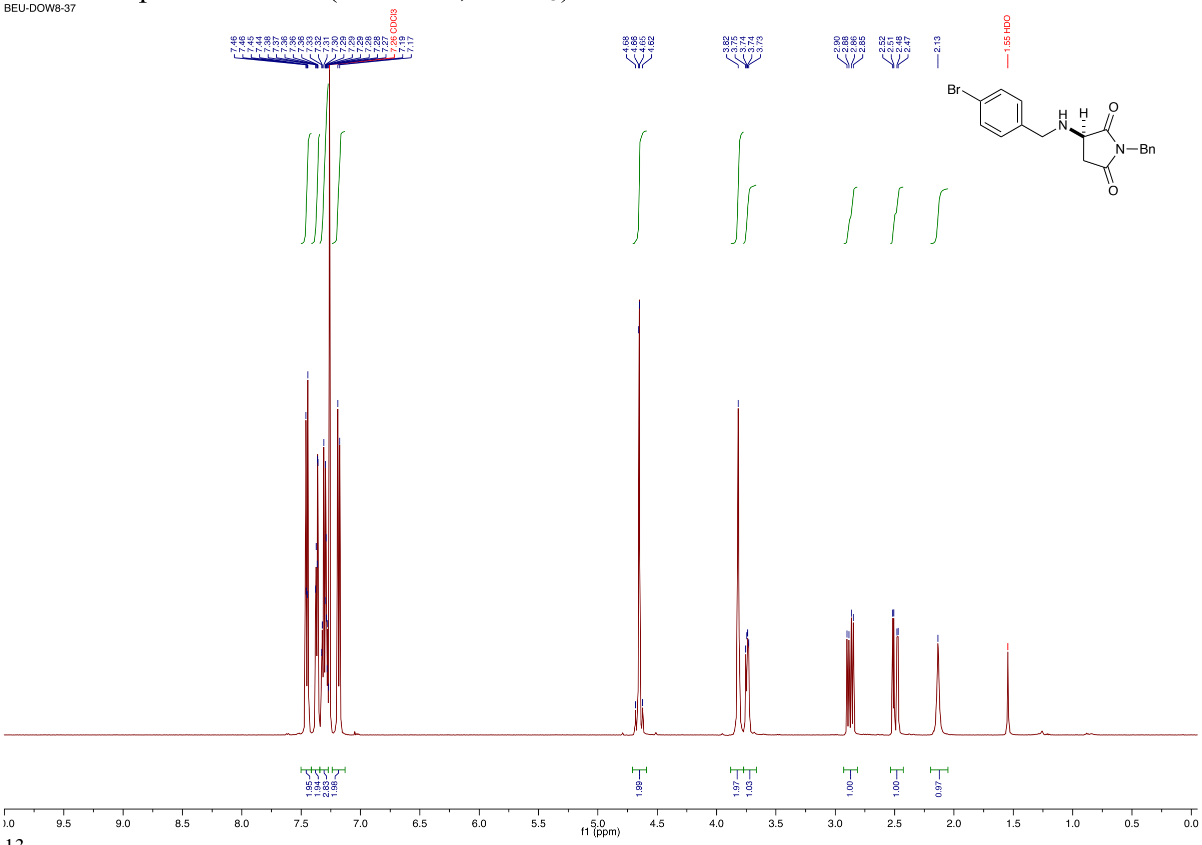

^{13}C NMR spectrum of **10** (126 MHz, $CDCl_3$)

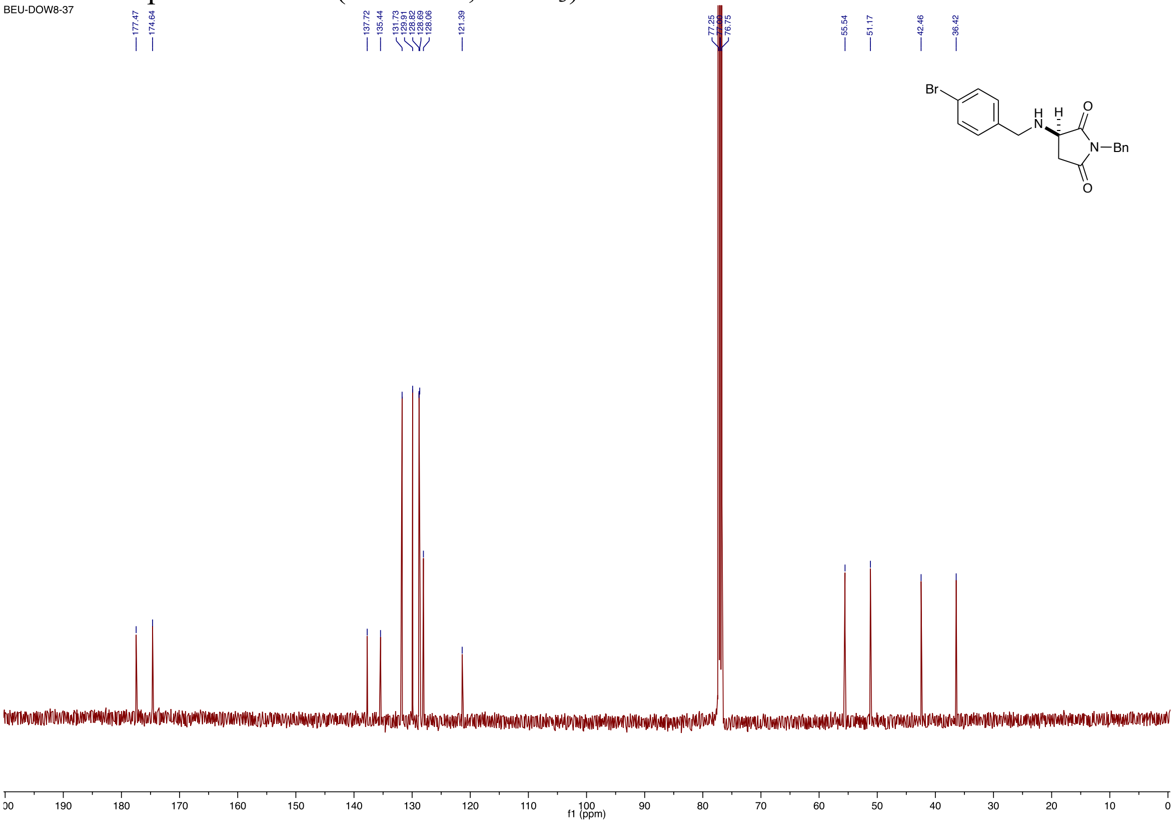

^{1}H NMR spectrum of **11** (500 MHz, $CDCl_3$)

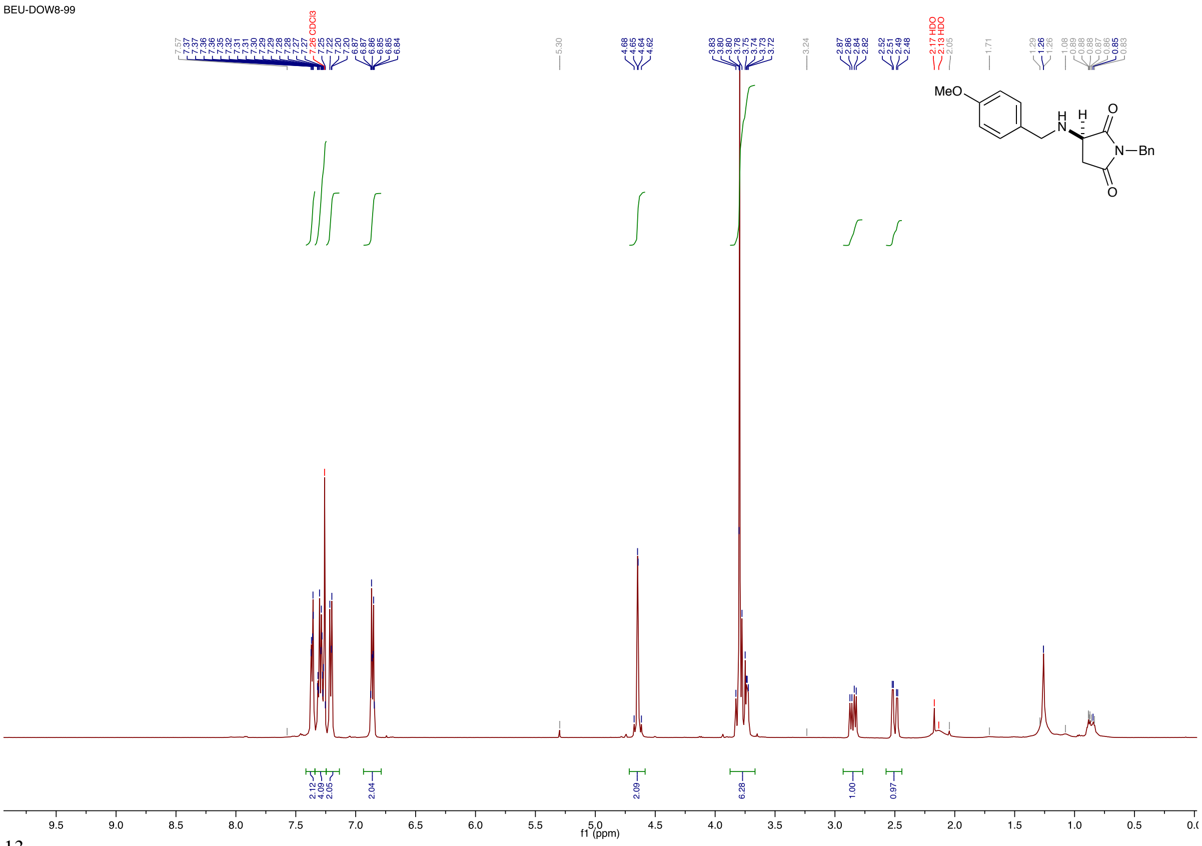

^{13}C NMR spectrum of **11** (126 MHz, $CDCl_3$)

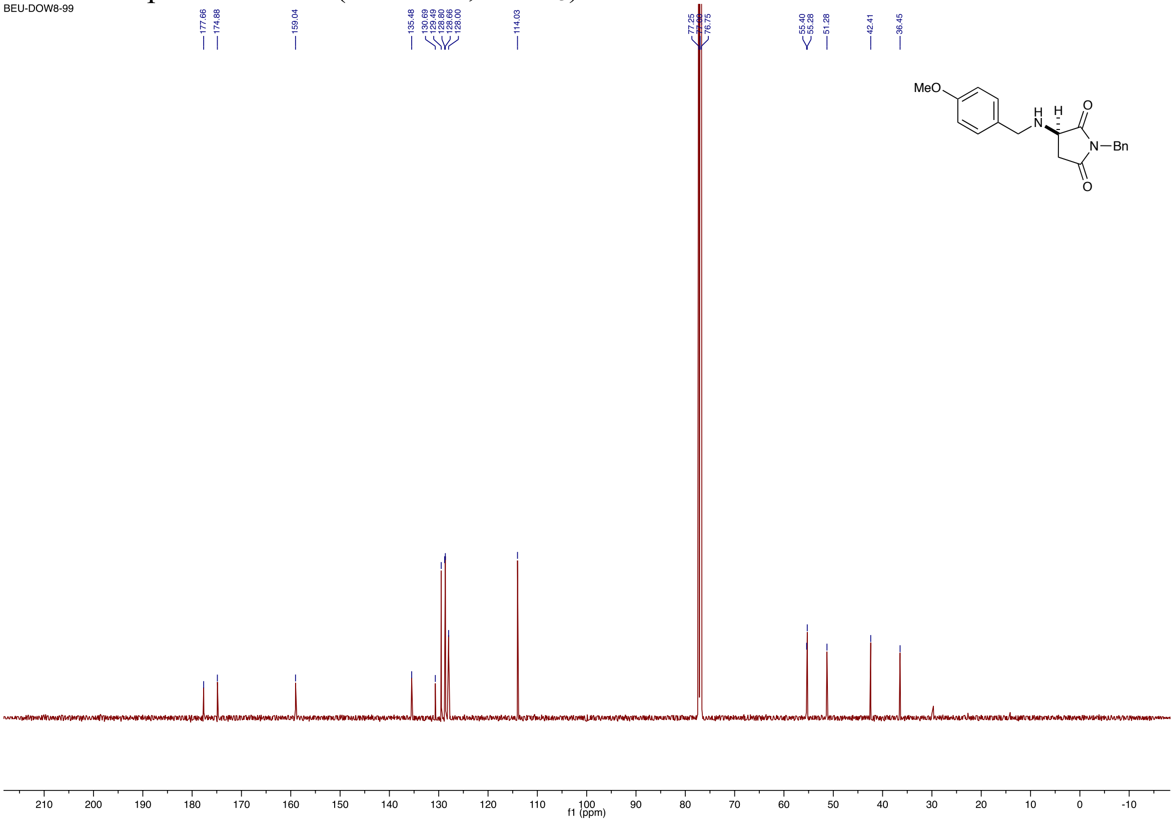

^{1}H NMR spectrum of **12** (500 MHz, $CDCl_3$)

BEU-DOW8-50

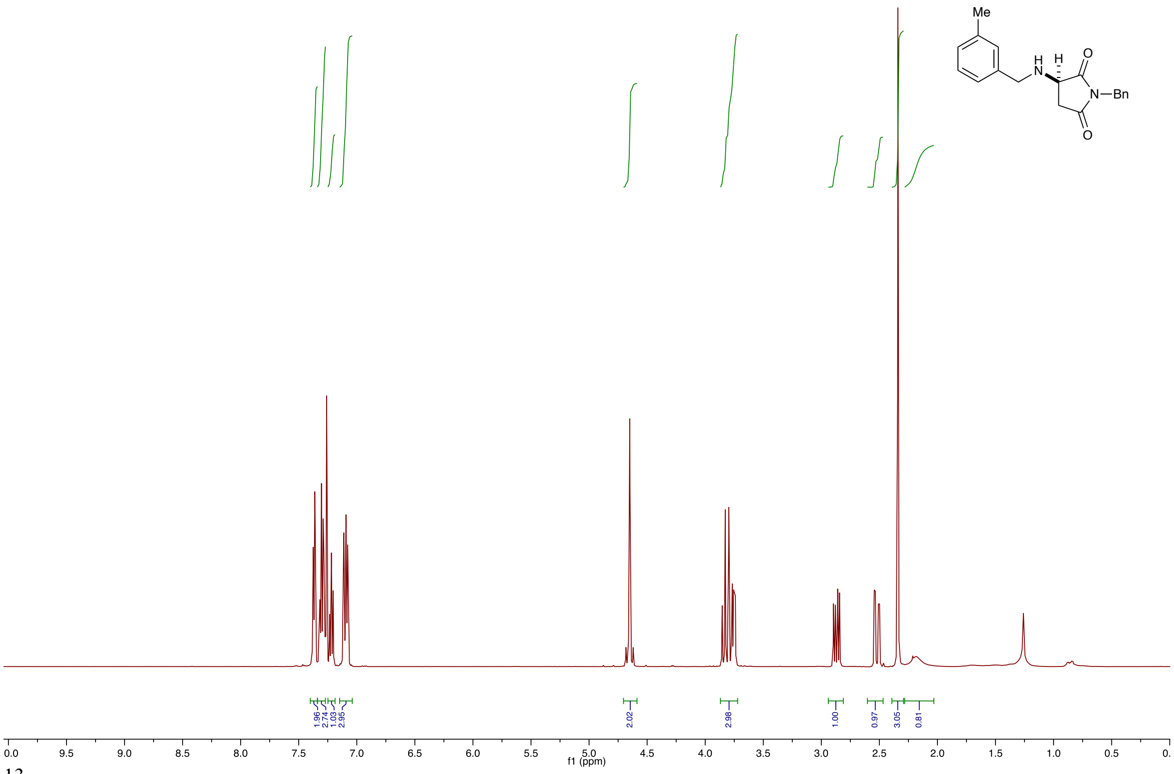

^{13}C NMR spectrum of **12** (126 MHz, $CDCl_3$)

BEU-DOW8-50

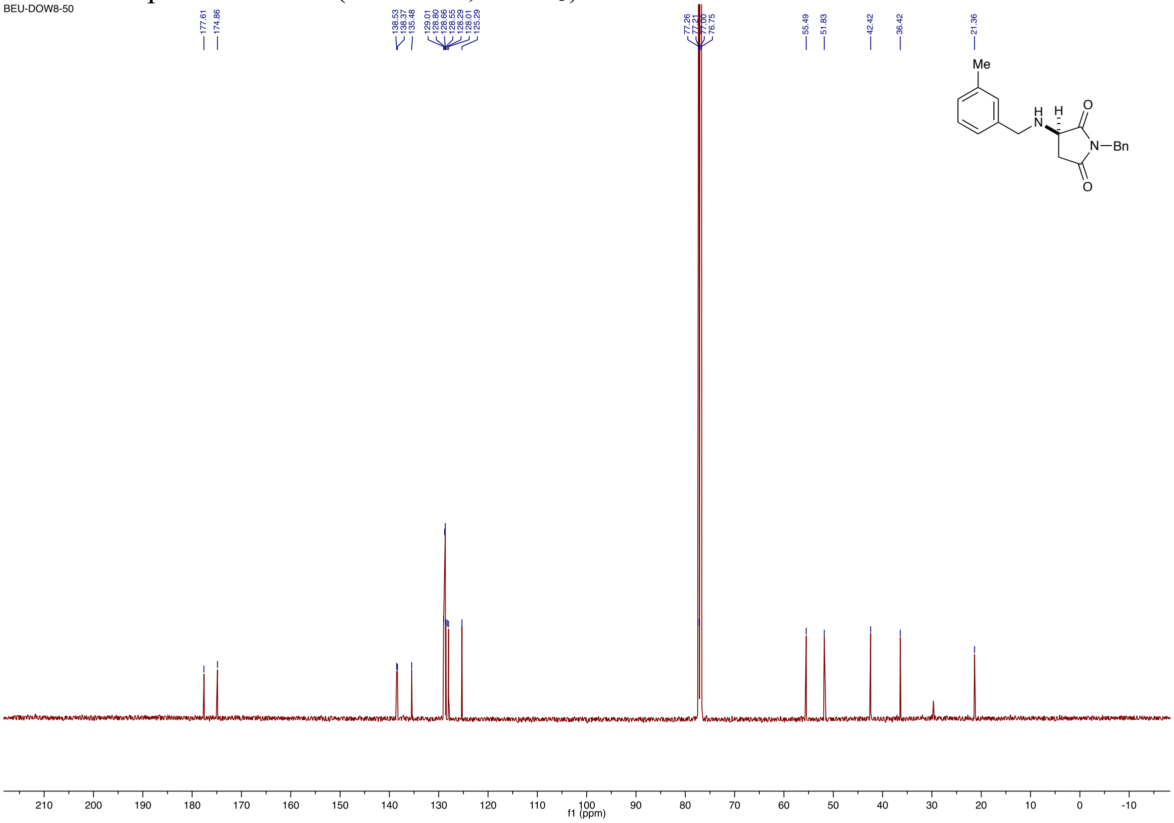

^{1}H NMR spectrum of **13** (500 MHz, $CDCl_3$)

RDD03-25

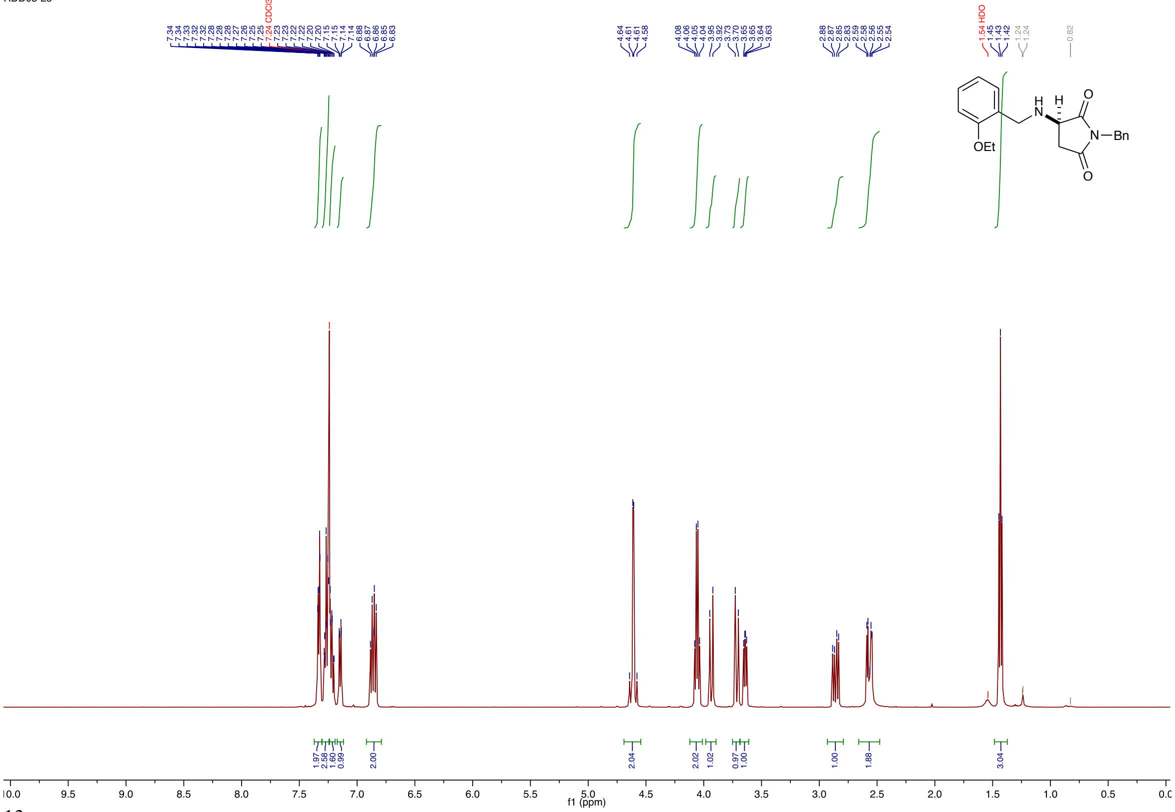

^{13}C NMR spectrum of **13** (126 MHz, $CDCl_3$)

RDD03-25

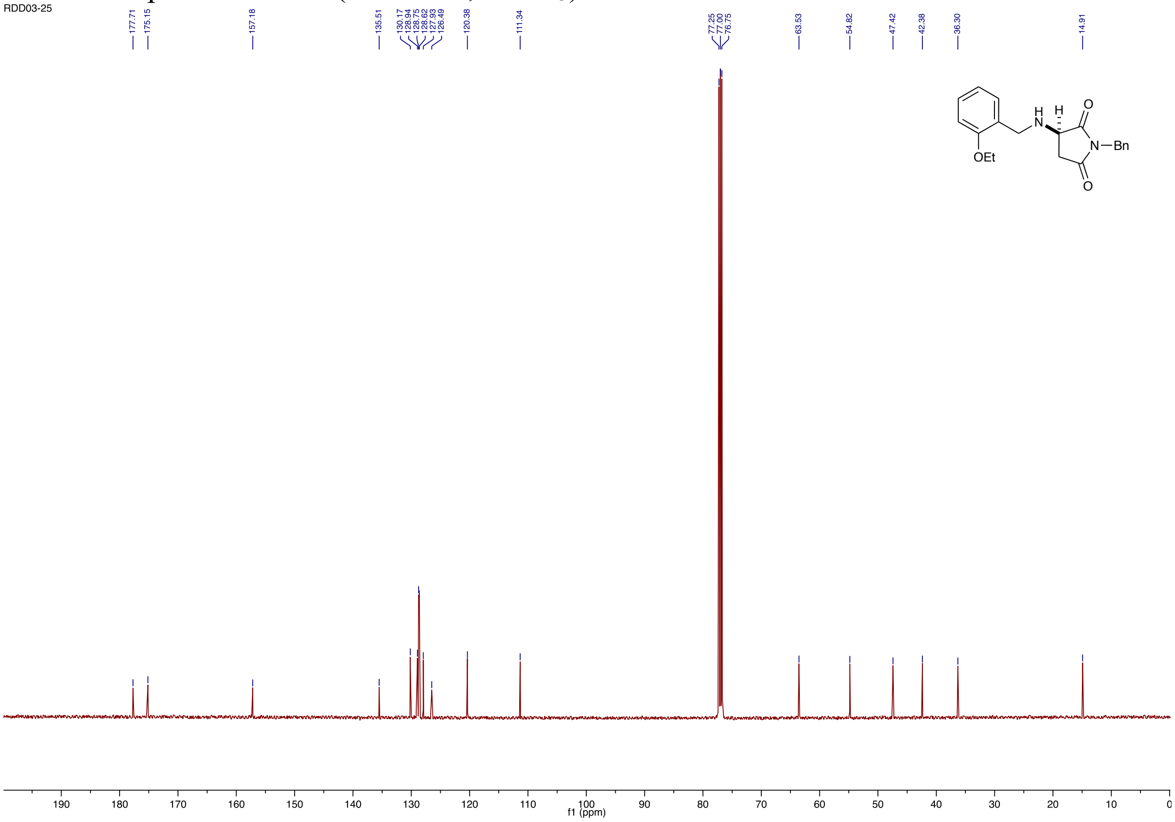

^{1}H NMR spectrum of **14** (500 MHz, $CDCl_3$)

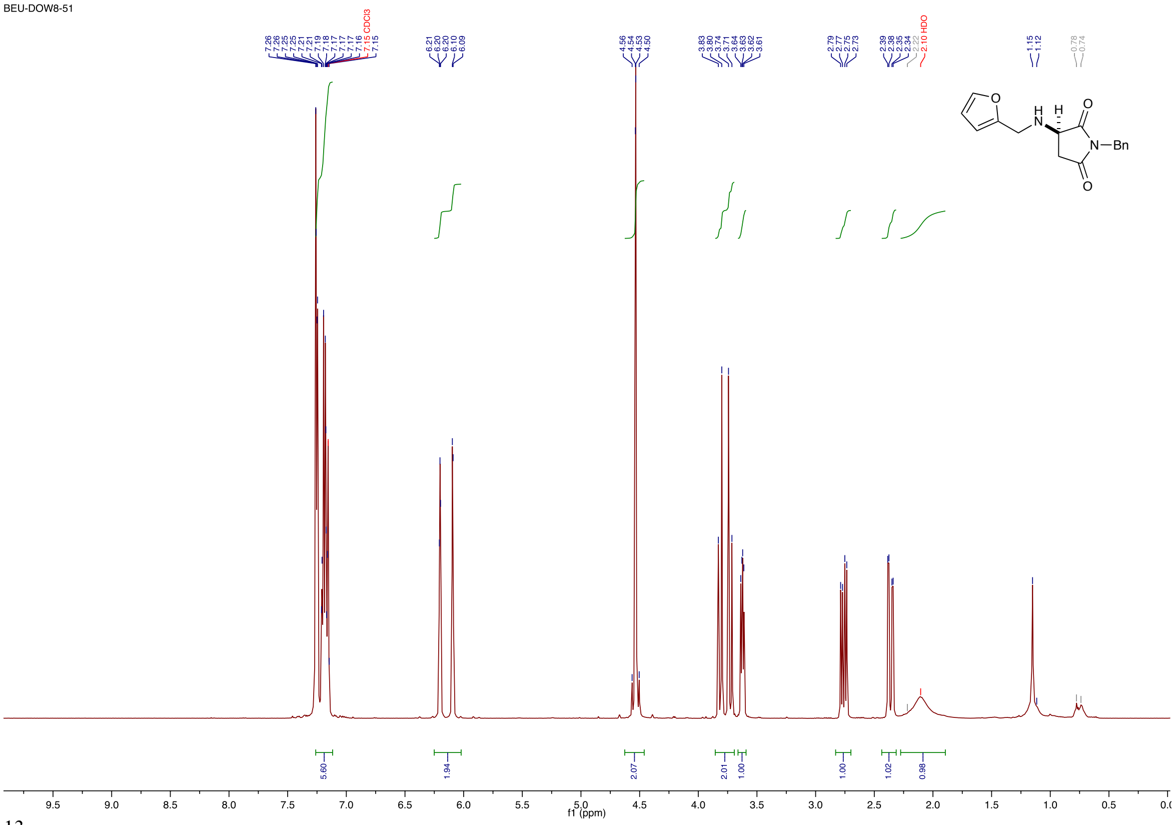

^{13}C NMR spectrum of **14** (126 MHz, $CDCl_3$)

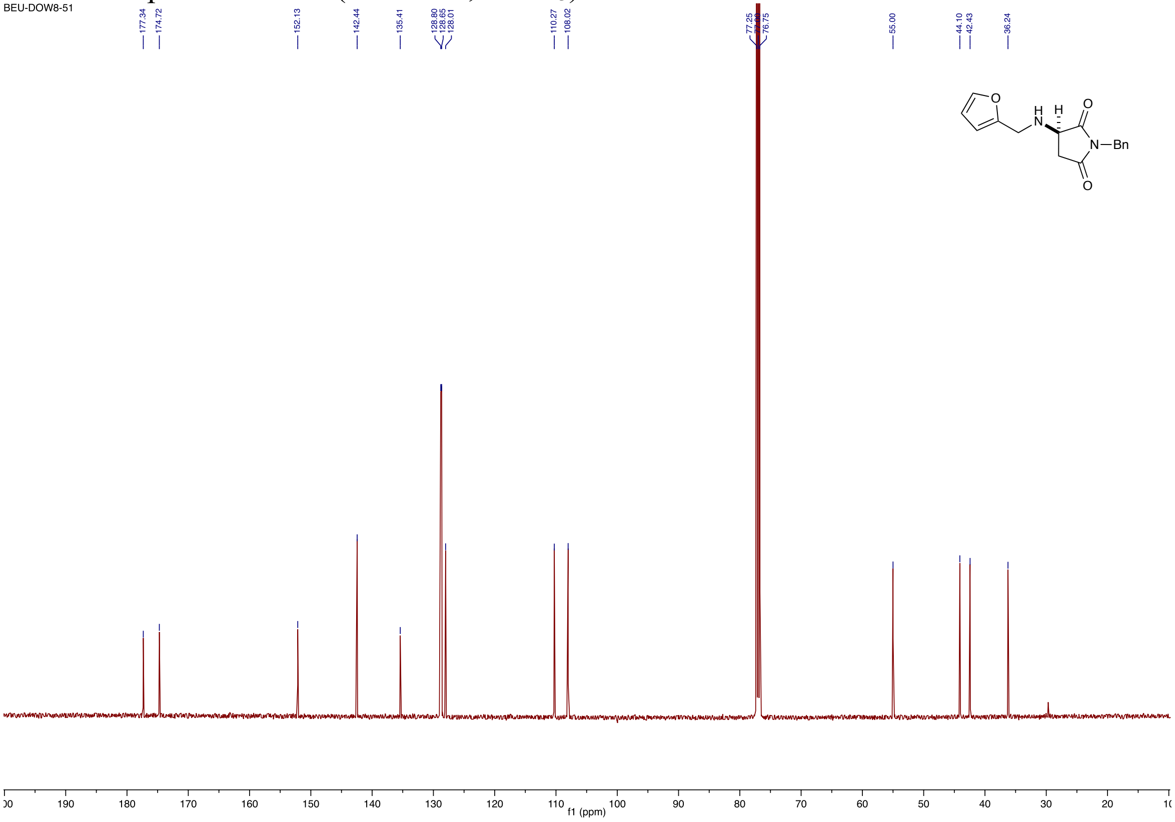

^{1}H NMR spectrum of **15** (500 MHz, $CDCl_3$)

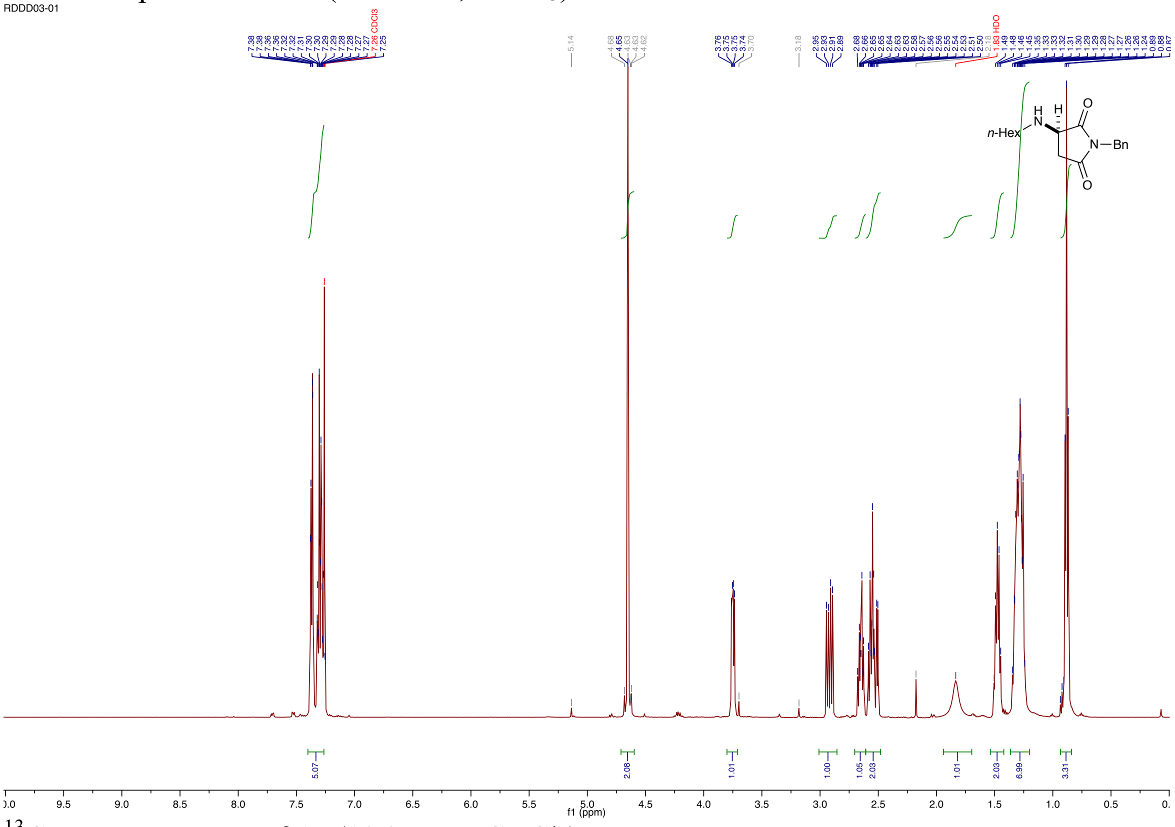

^{13}C NMR spectrum of **15** (126 MHz, $CDCl_3$)

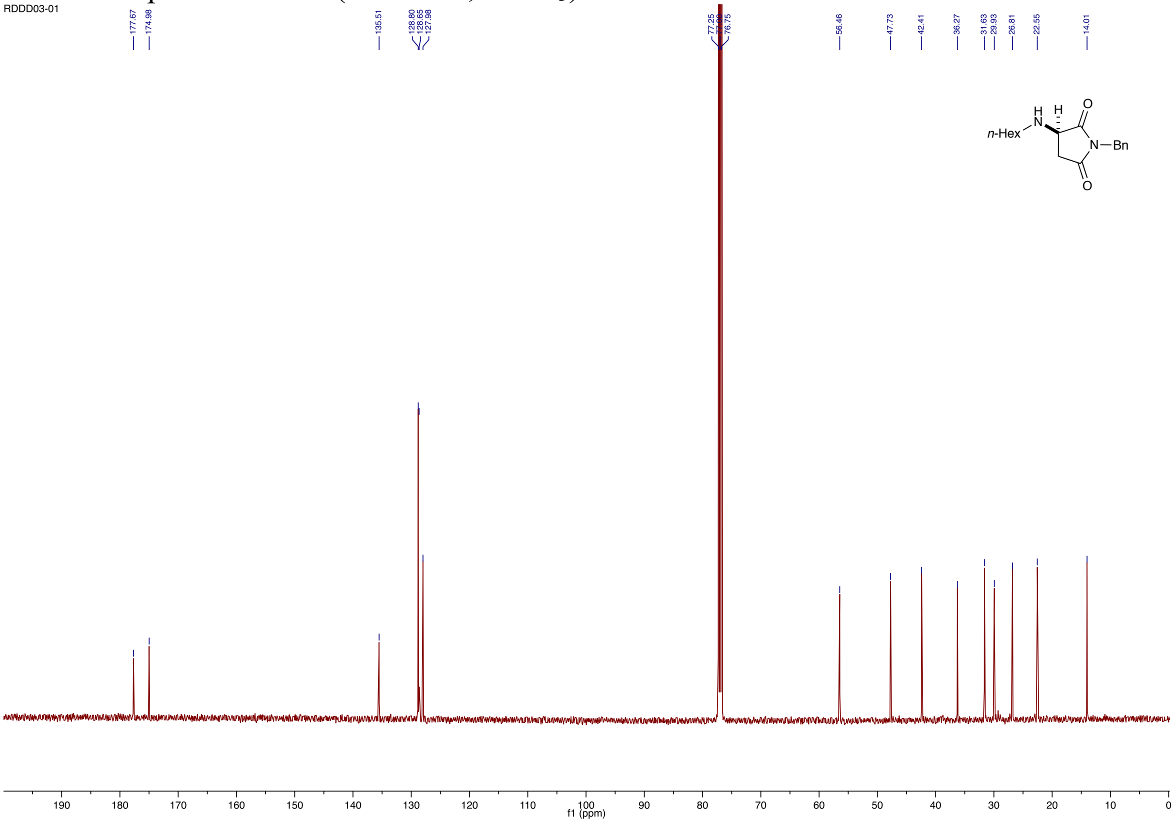

^{1}H NMR spectrum of **16** (500 MHz, $CDCl_3$)

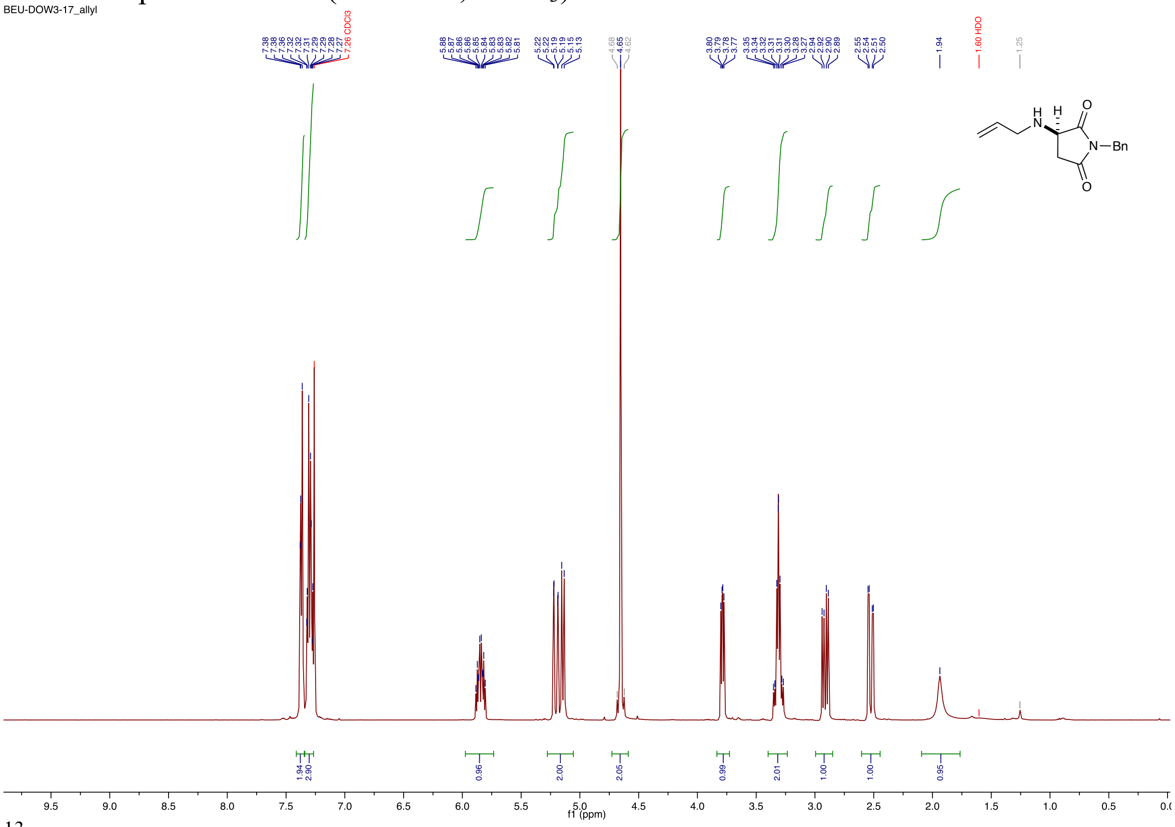

^{13}C NMR spectrum of **16** (126 MHz, $CDCl_3$)

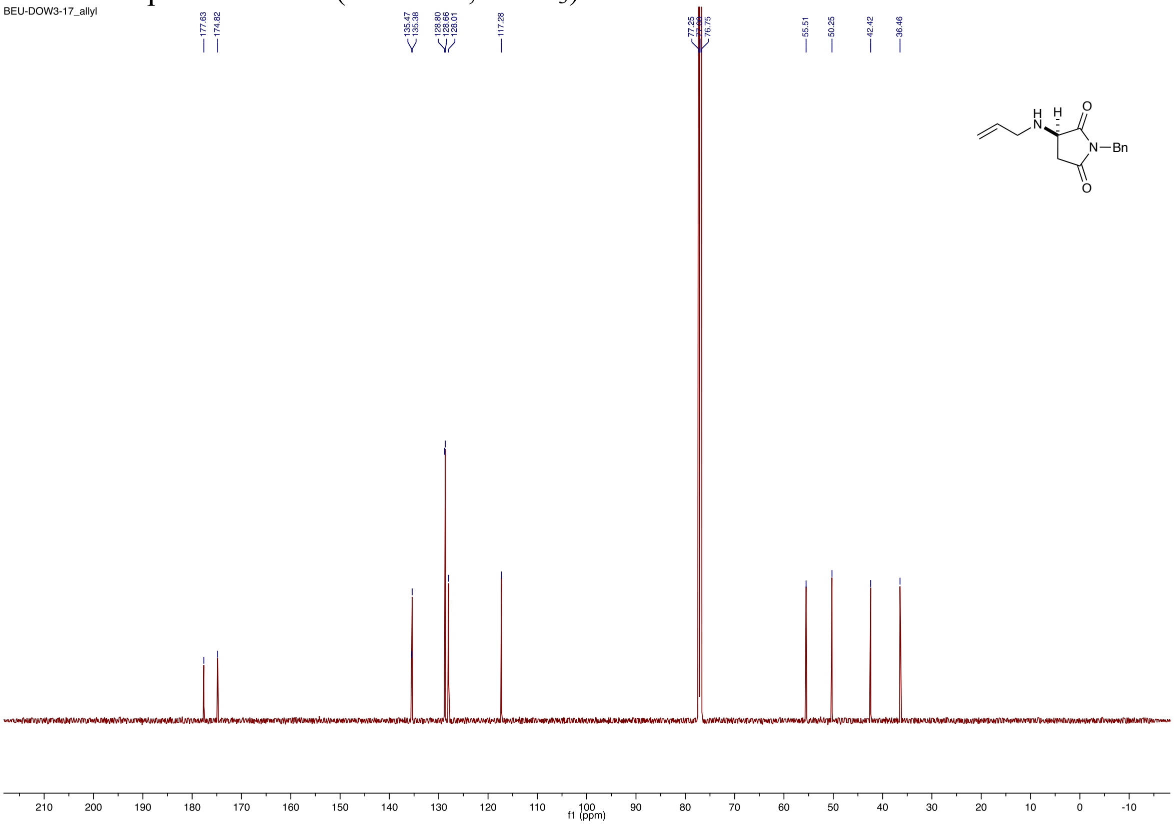

^{1}H NMR spectrum of **17** (500 MHz, $CDCl_3$)

BEU-DOW8-52

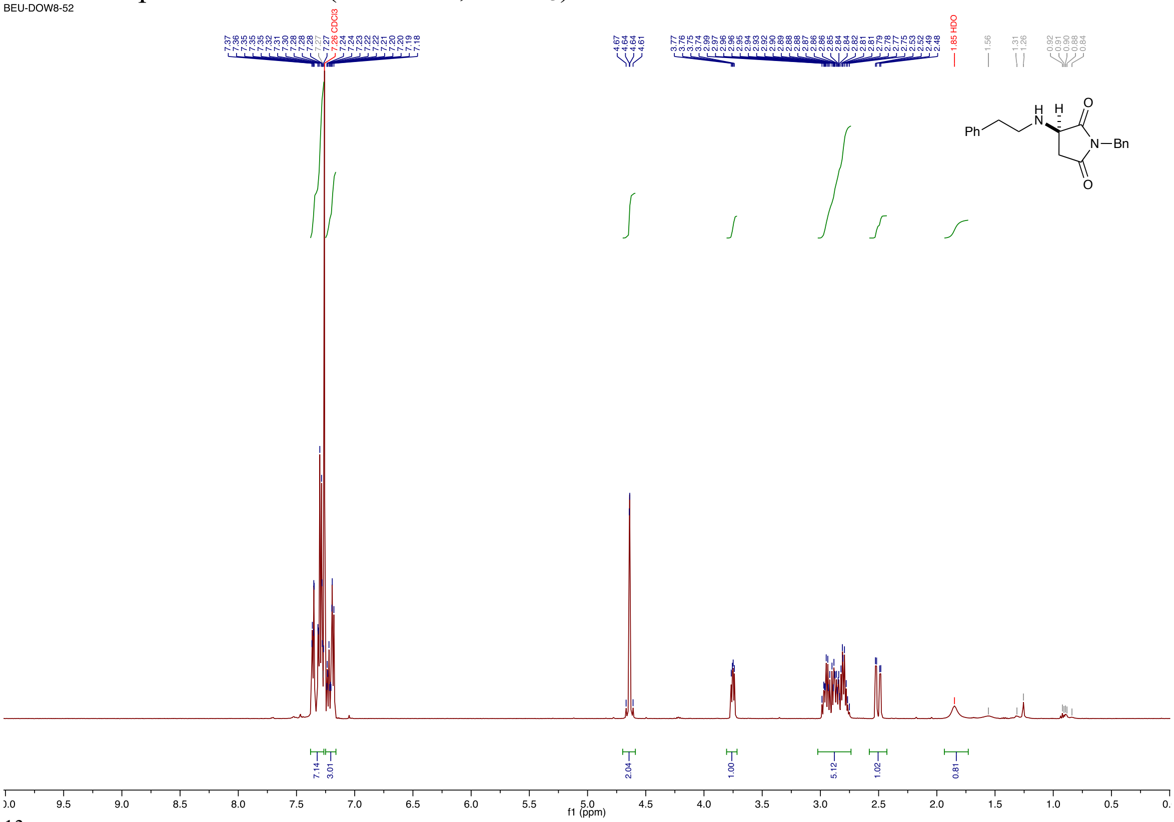

^{13}C NMR spectrum of **17** (126 MHz, $CDCl_3$)

BEU-DOW8-52

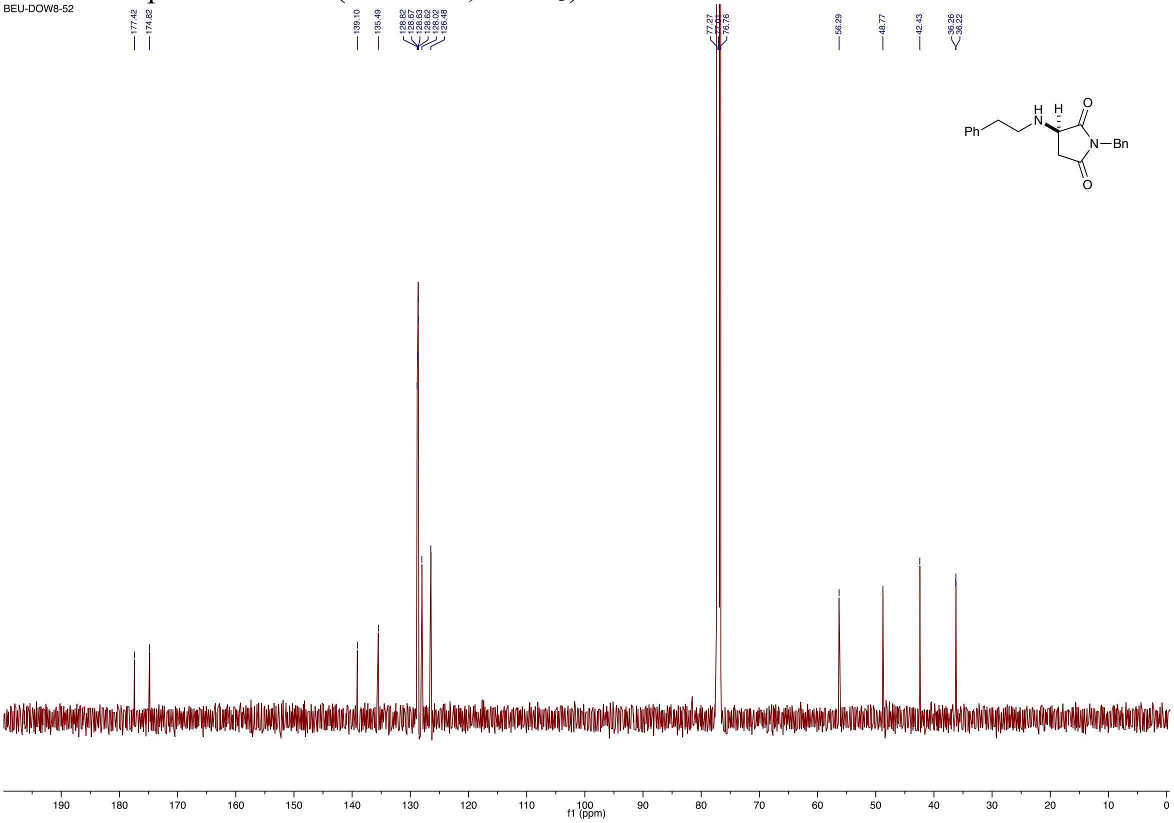

^{1}H NMR spectrum of **18** (500 MHz, $CDCl_3$)

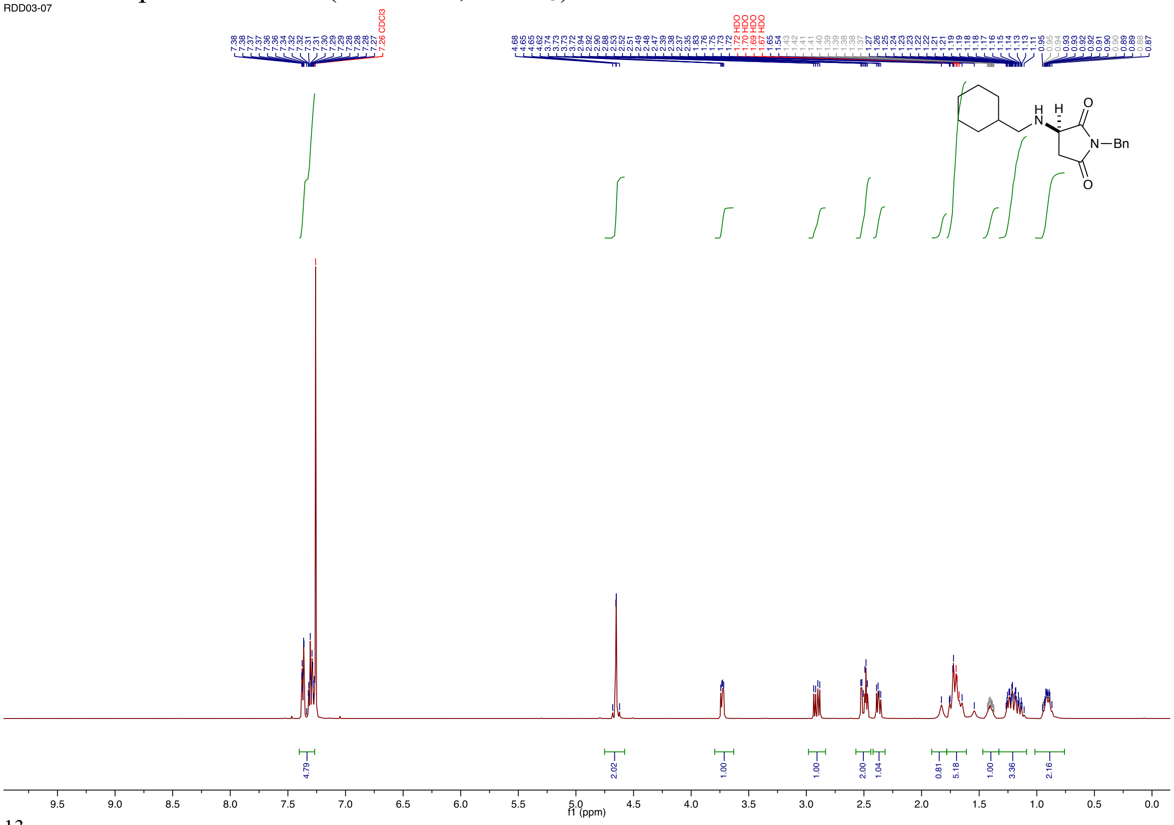

^{13}C NMR spectrum of **18** (126 MHz, $CDCl_3$)

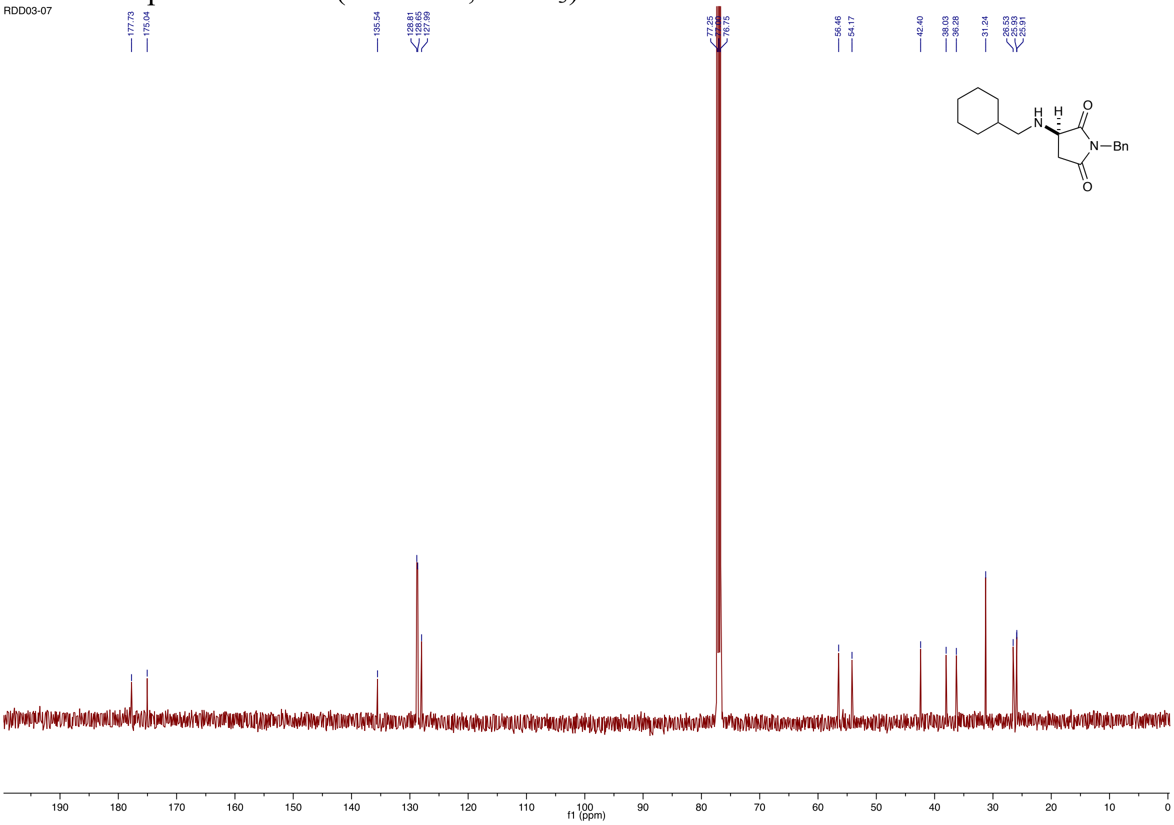

^{1}H NMR spectrum of **19** (500 MHz, $CDCl_3$)

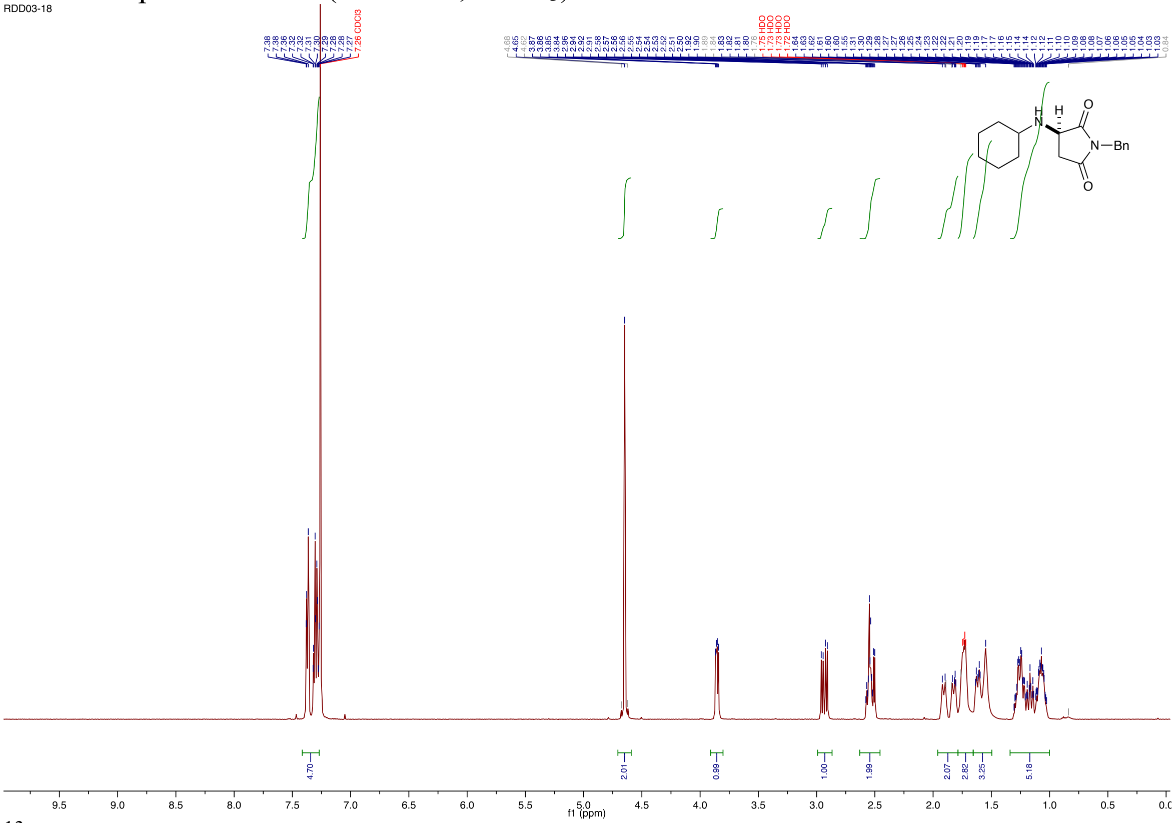

^{13}C NMR spectrum of **19** (126 MHz, $CDCl_3$)

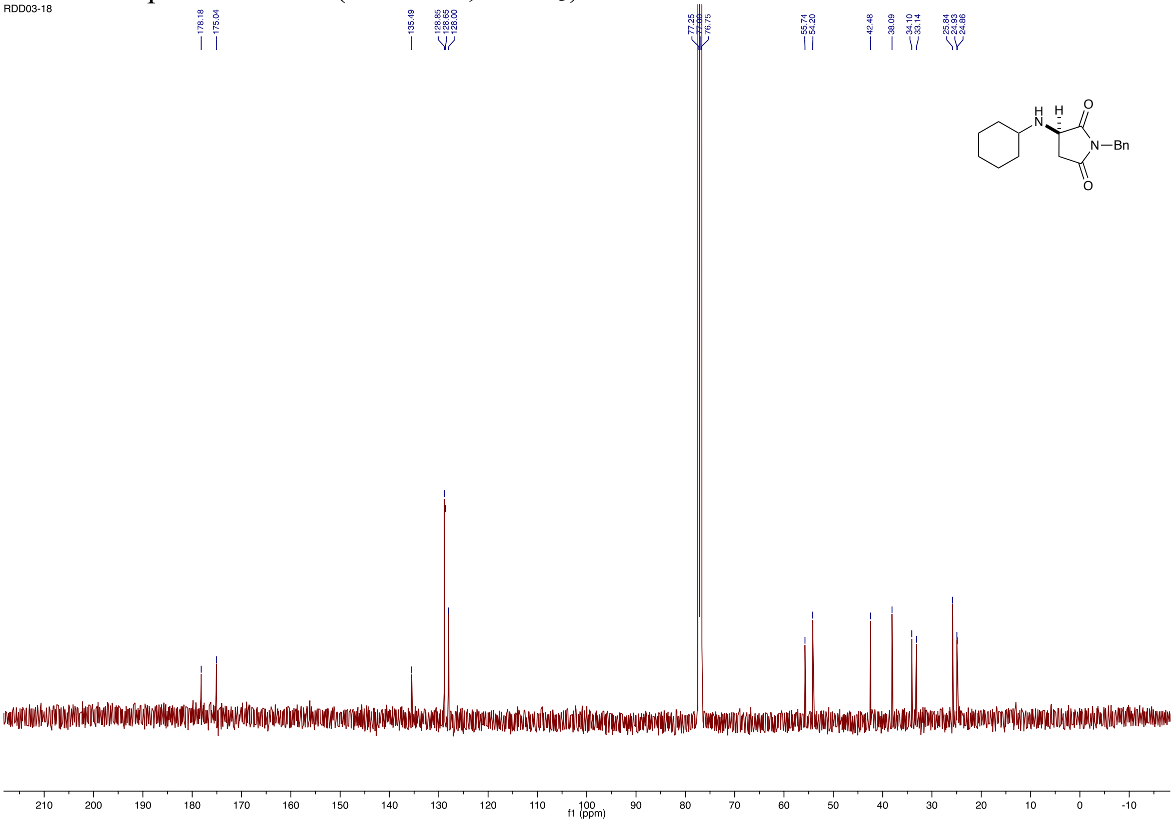

^{1}H NMR spectrum of **20** (500 MHz, $CDCl_3$)

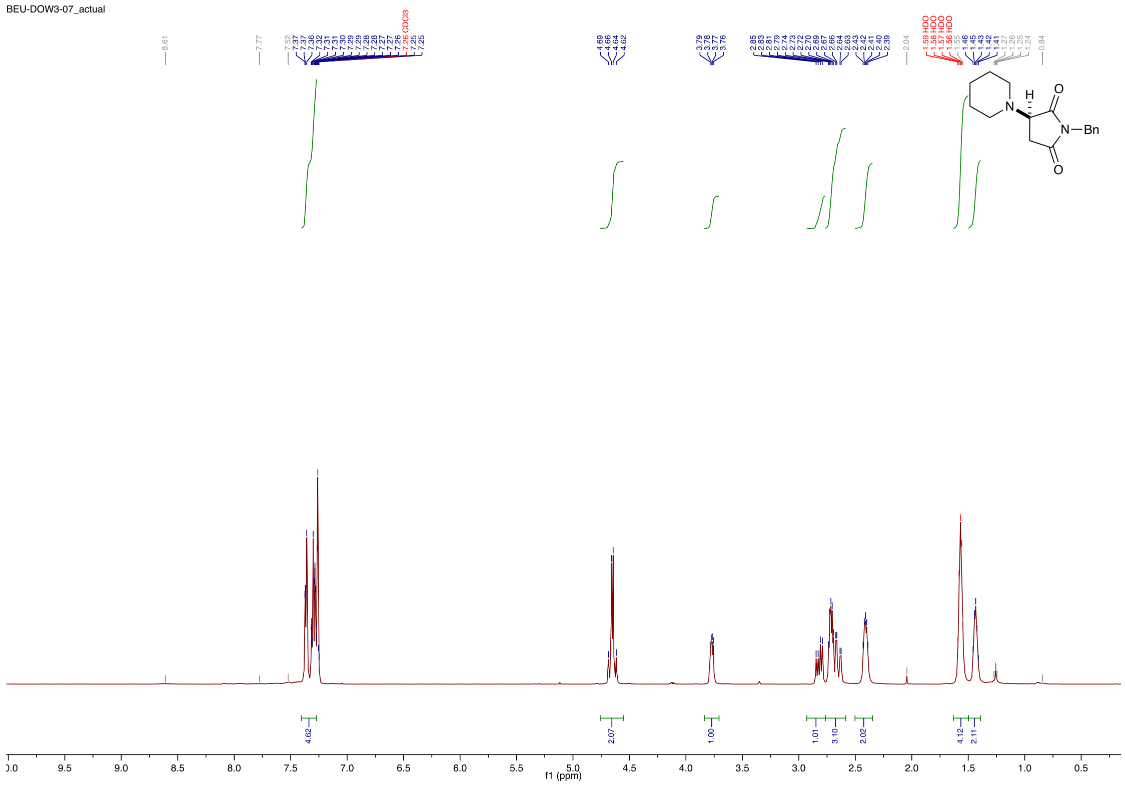

^{13}C NMR spectrum of **20** (126 MHz, $CDCl_3$)

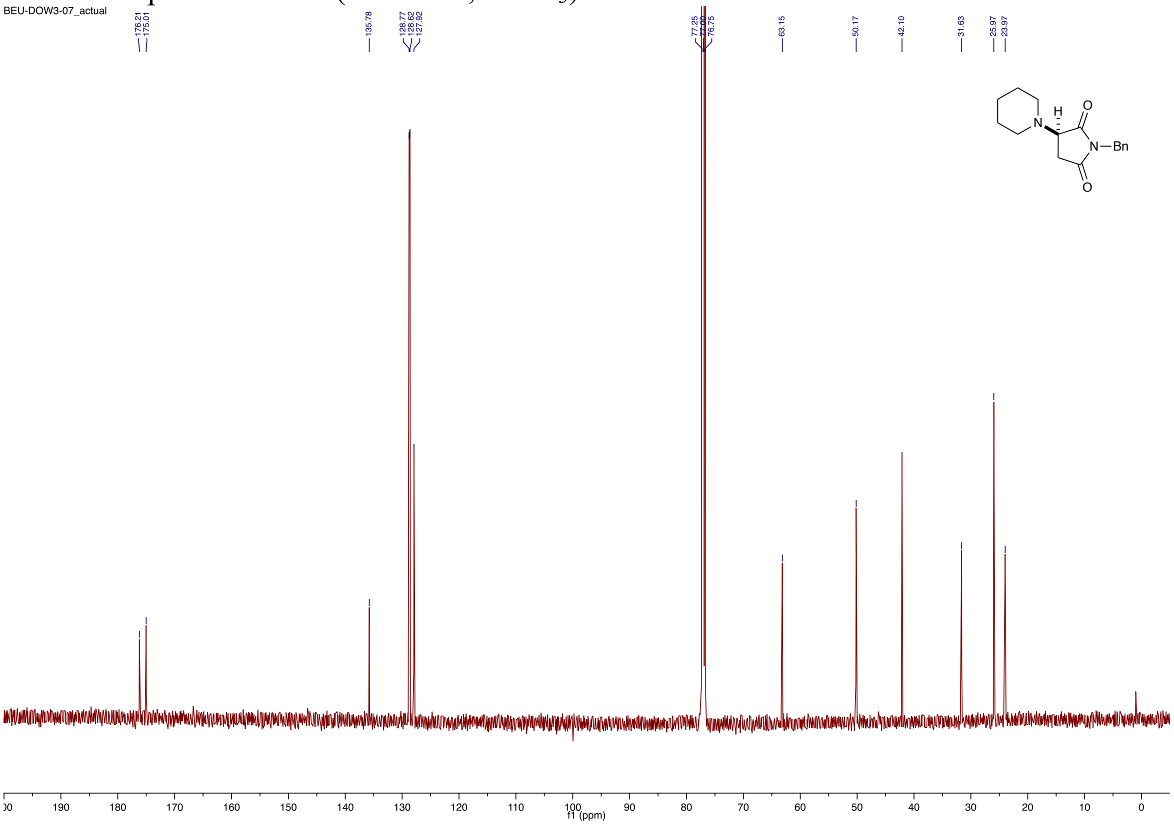

^{1}H NMR spectrum of **21** (500 MHz, $CDCl_3$)

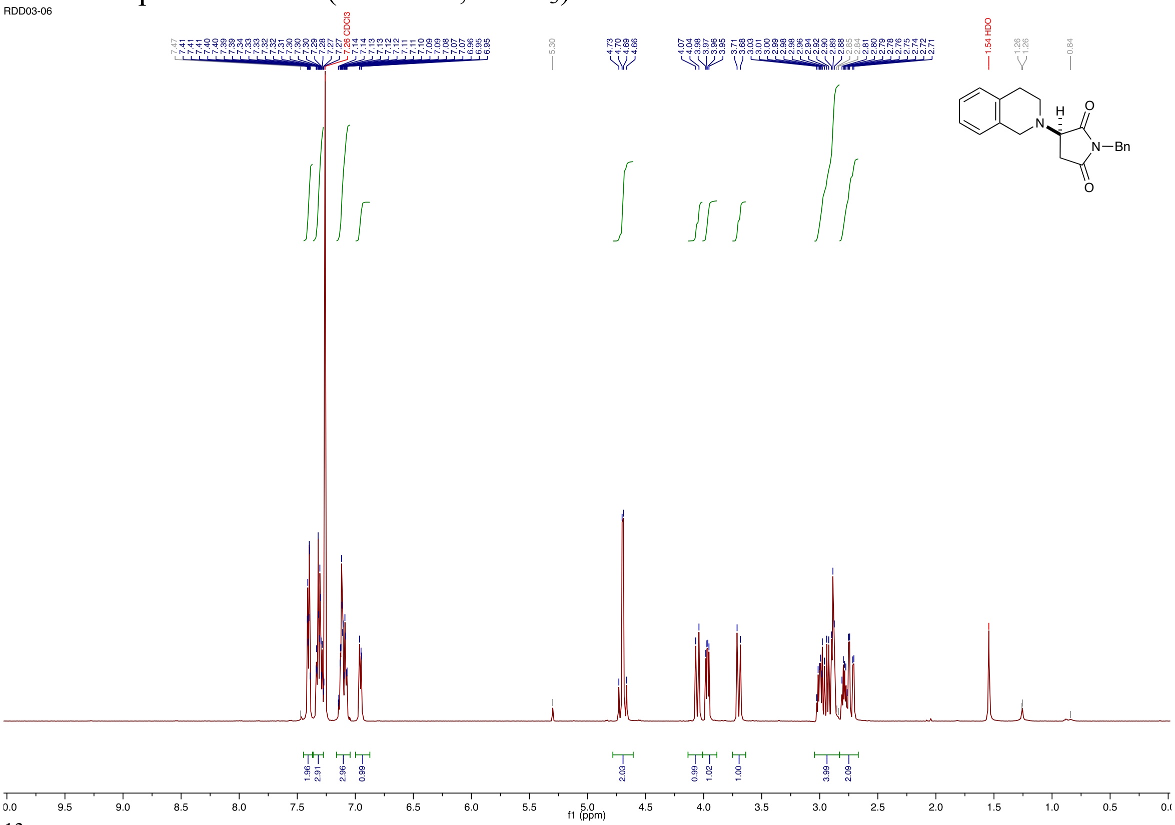

^{13}C NMR spectrum of **21** (126 MHz, $CDCl_3$)

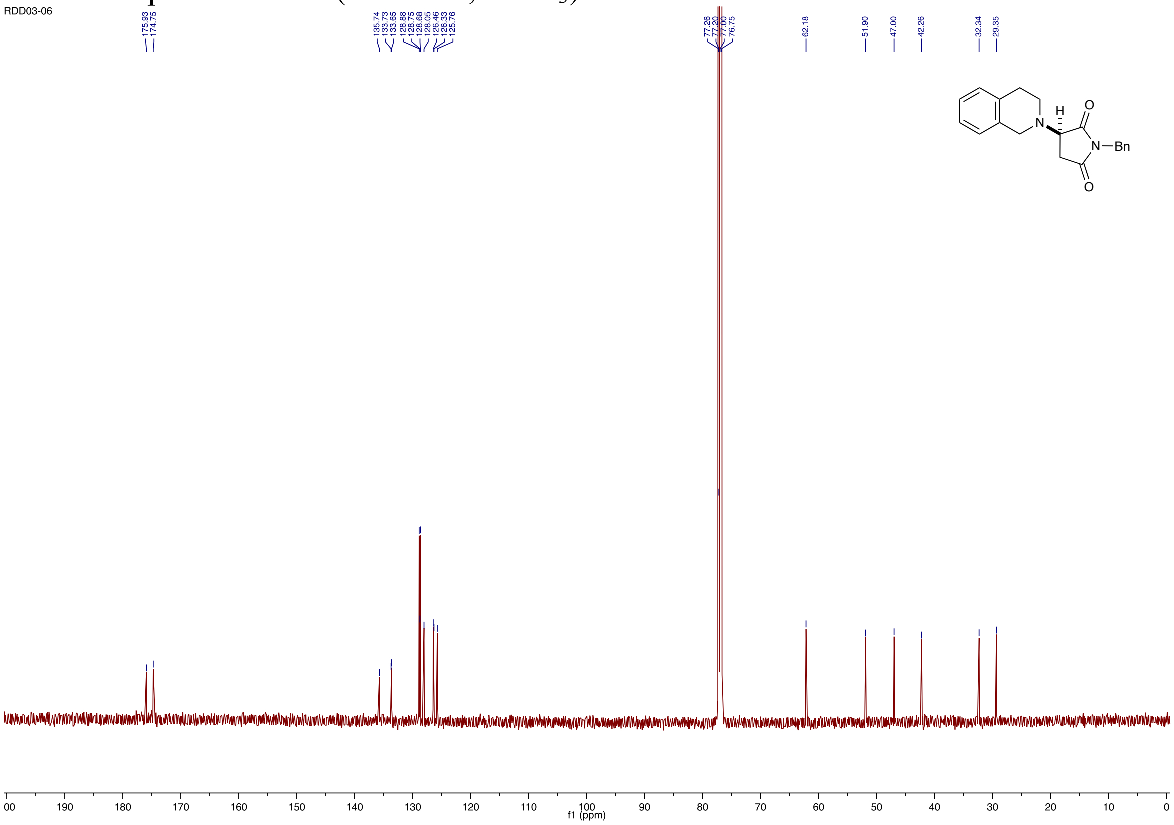

^{1}H NMR spectrum of **22** (500 MHz, $CDCl_3$)

RDD03-09

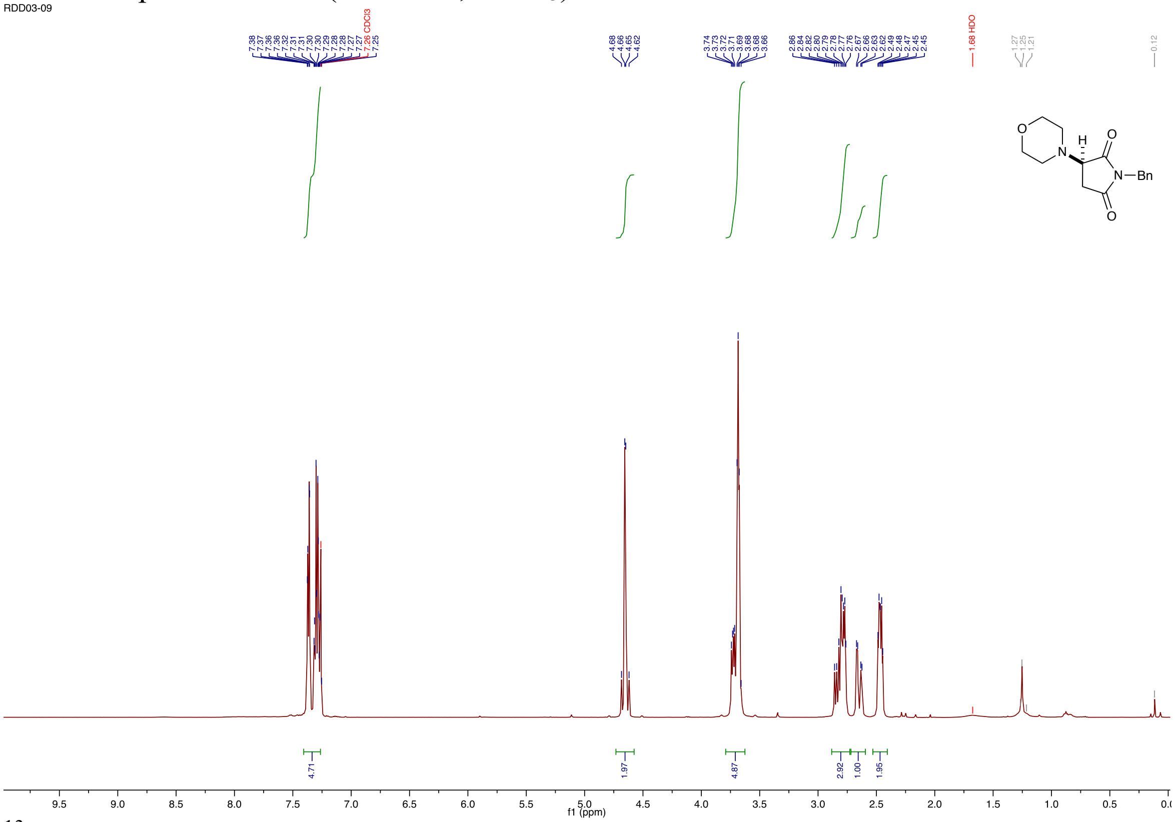

^{13}C NMR spectrum of **22** (126 MHz, $CDCl_3$)

RDD03-09

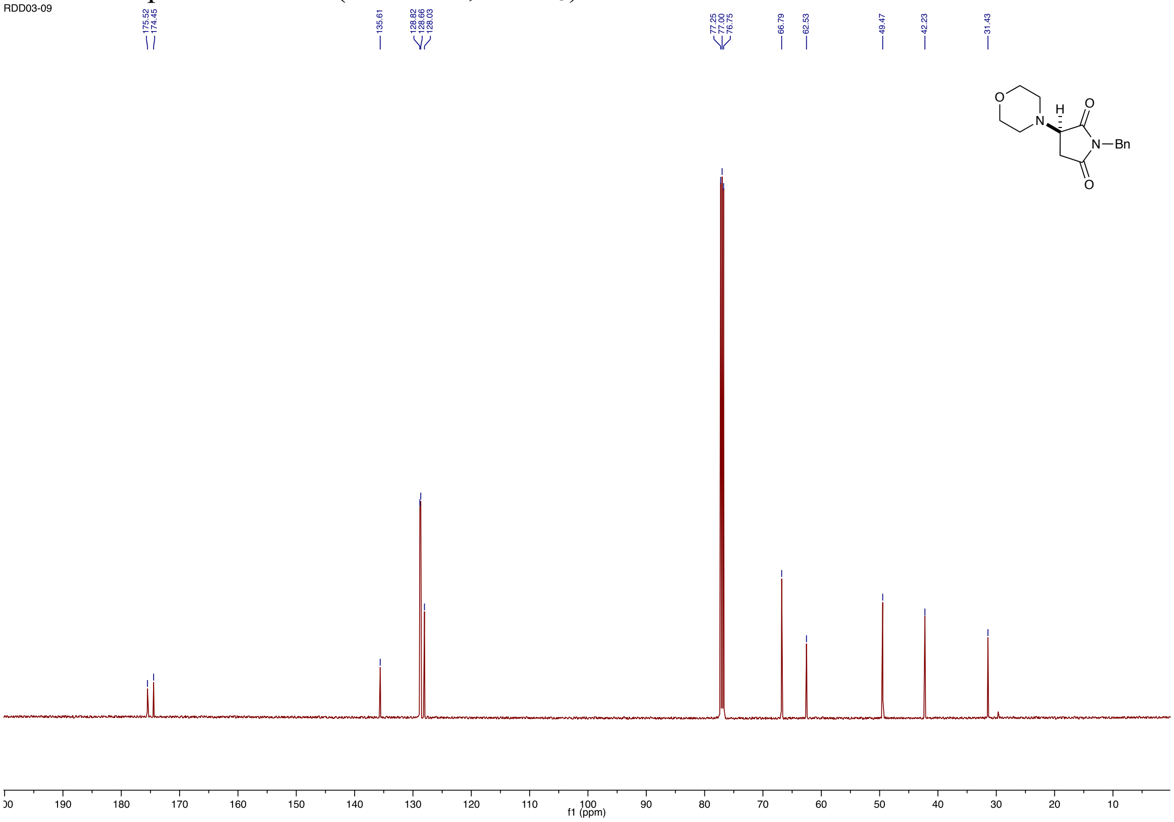

^{1}H NMR spectrum of **23** (500 MHz, $CDCl_3$)

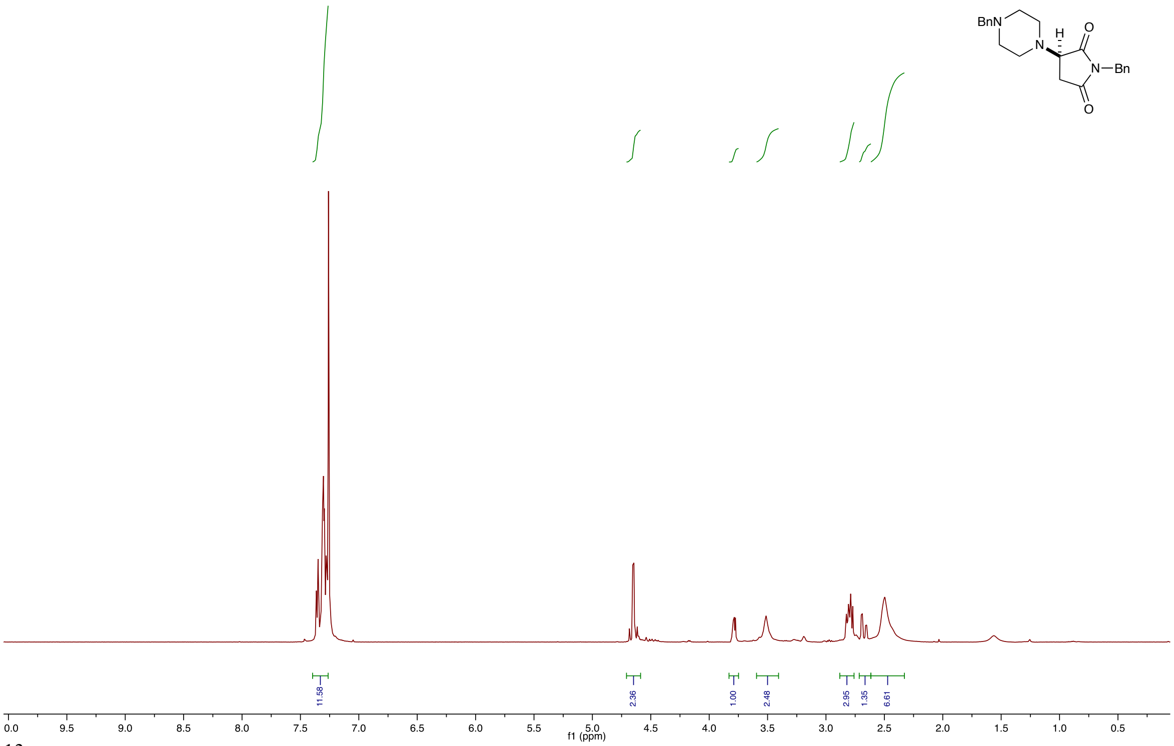

^{13}C NMR spectrum of **23** (126 MHz, $CDCl_3$)

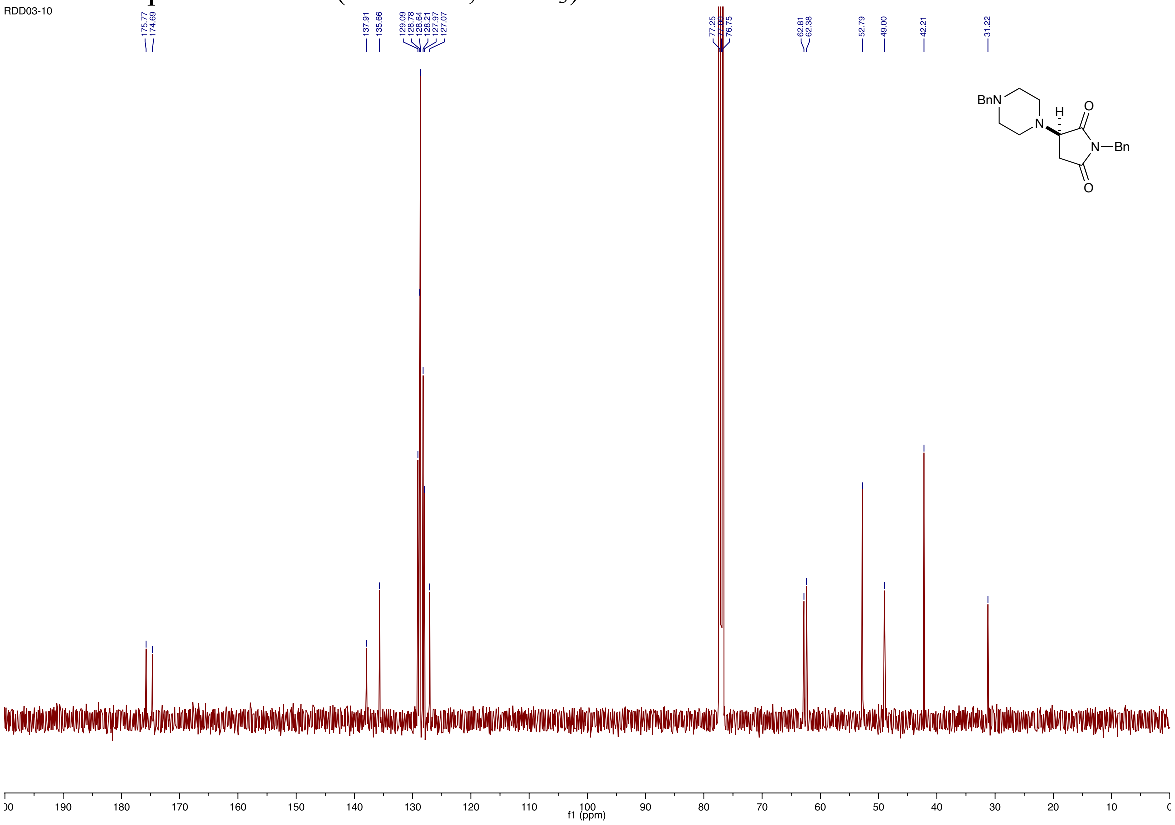

^{1}H NMR spectrum of **24** (500 MHz, $CDCl_3$)

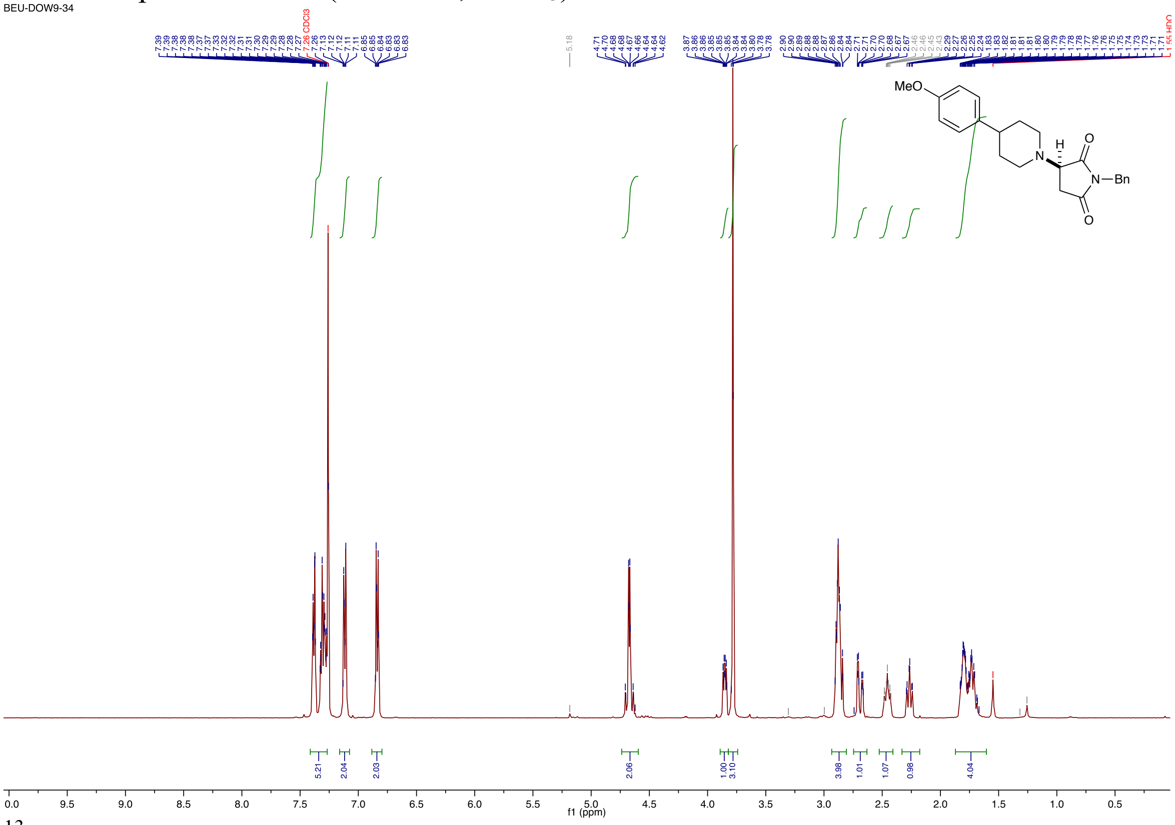

^{13}C NMR spectrum of **24** (126 MHz, $CDCl_3$)

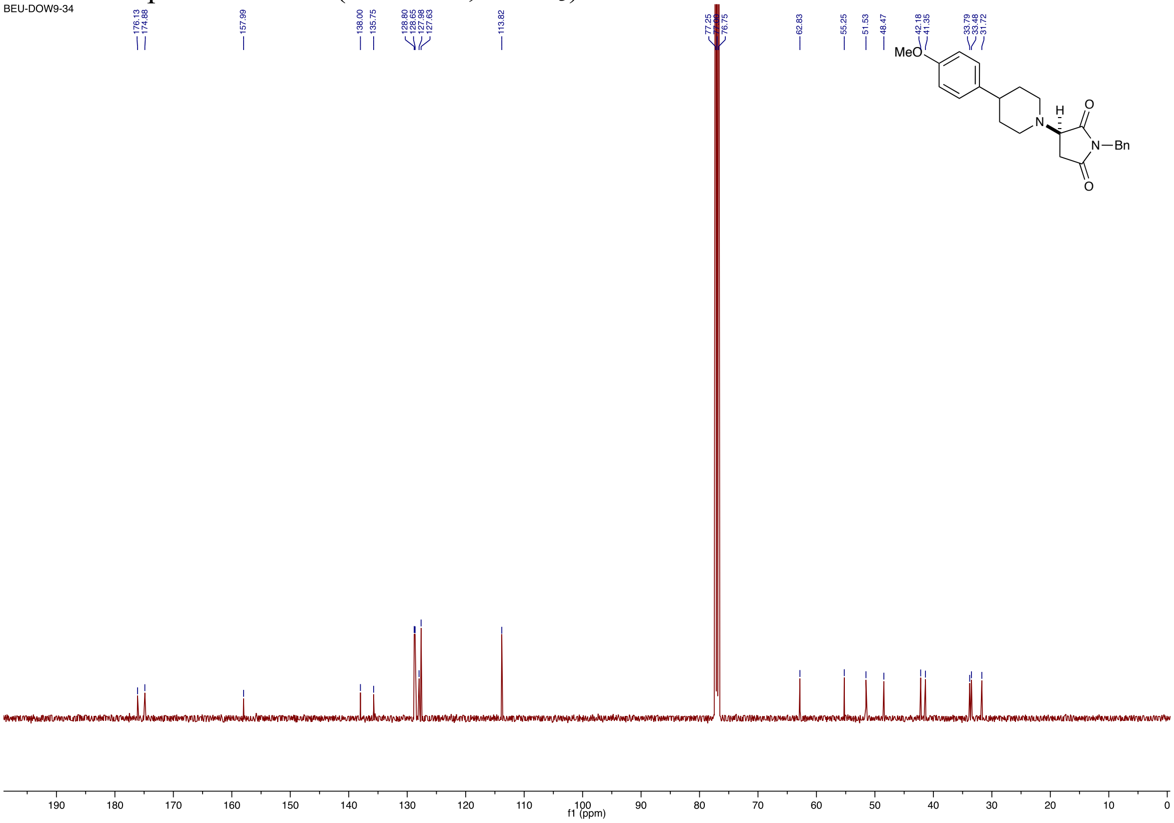

^{1}H NMR spectrum of **25** (500 MHz, $CDCl_3$)

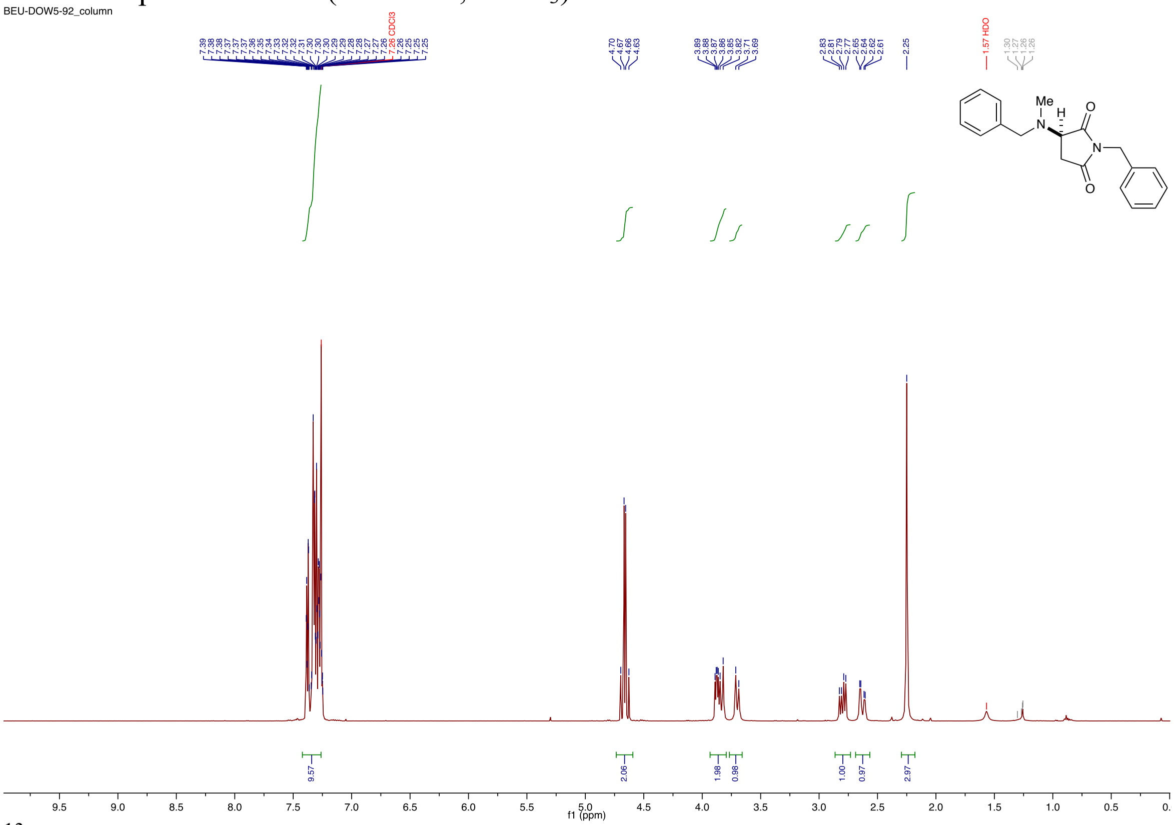

^{13}C NMR spectrum of **25** (126 MHz, $CDCl_3$)

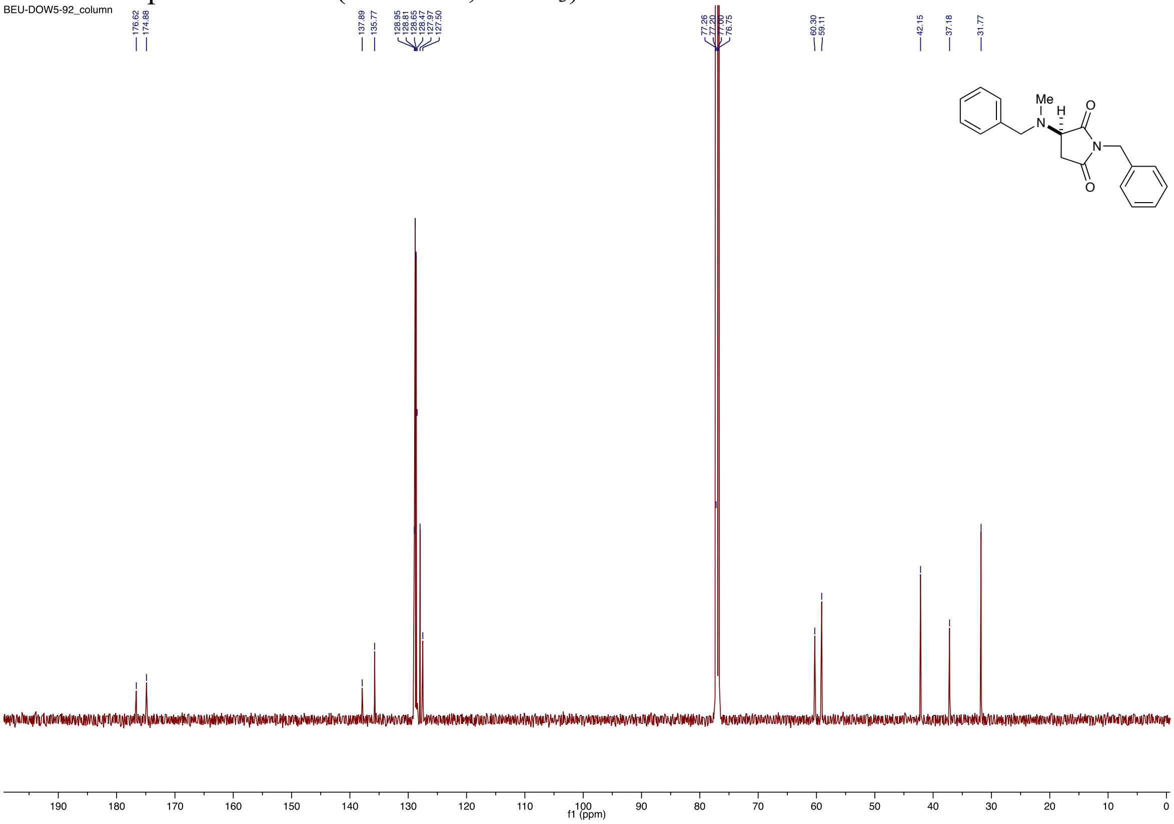

^{1}H NMR spectrum of **26** (500 MHz, $CDCl_3$)

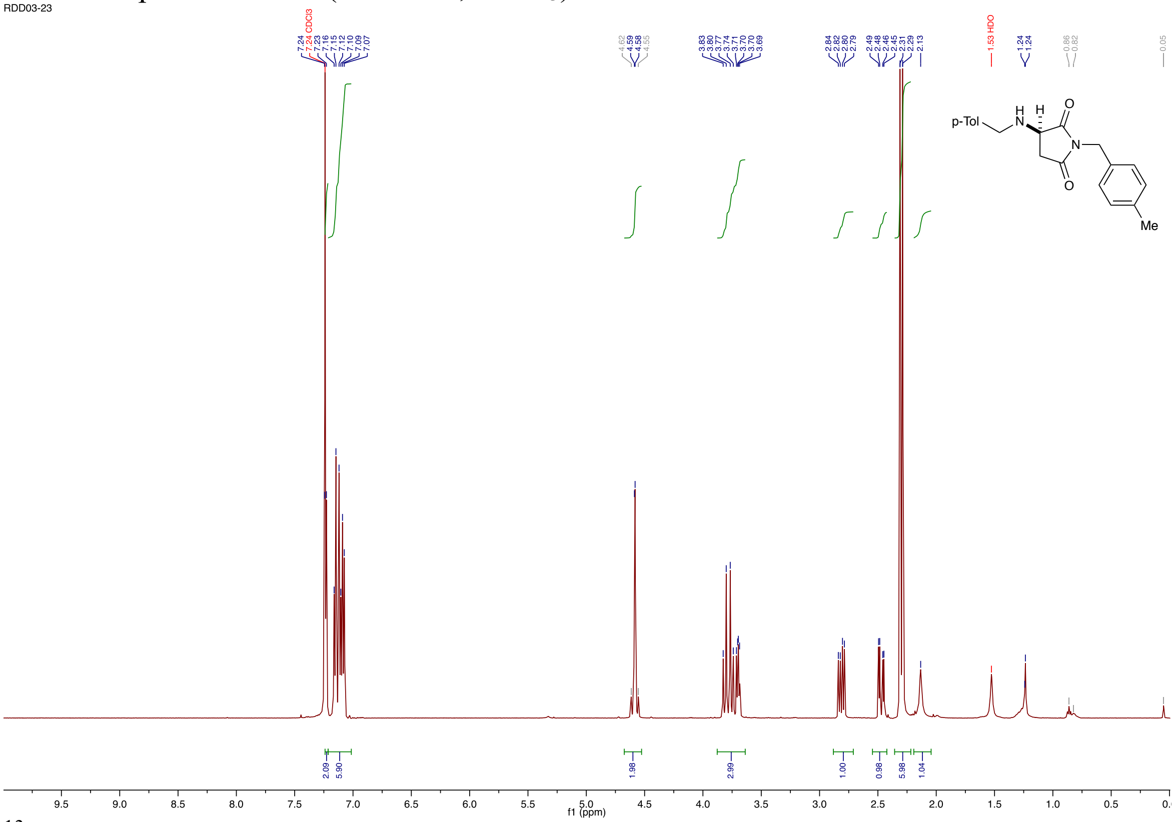

^{13}C NMR spectrum of **26** (126 MHz, $CDCl_3$)

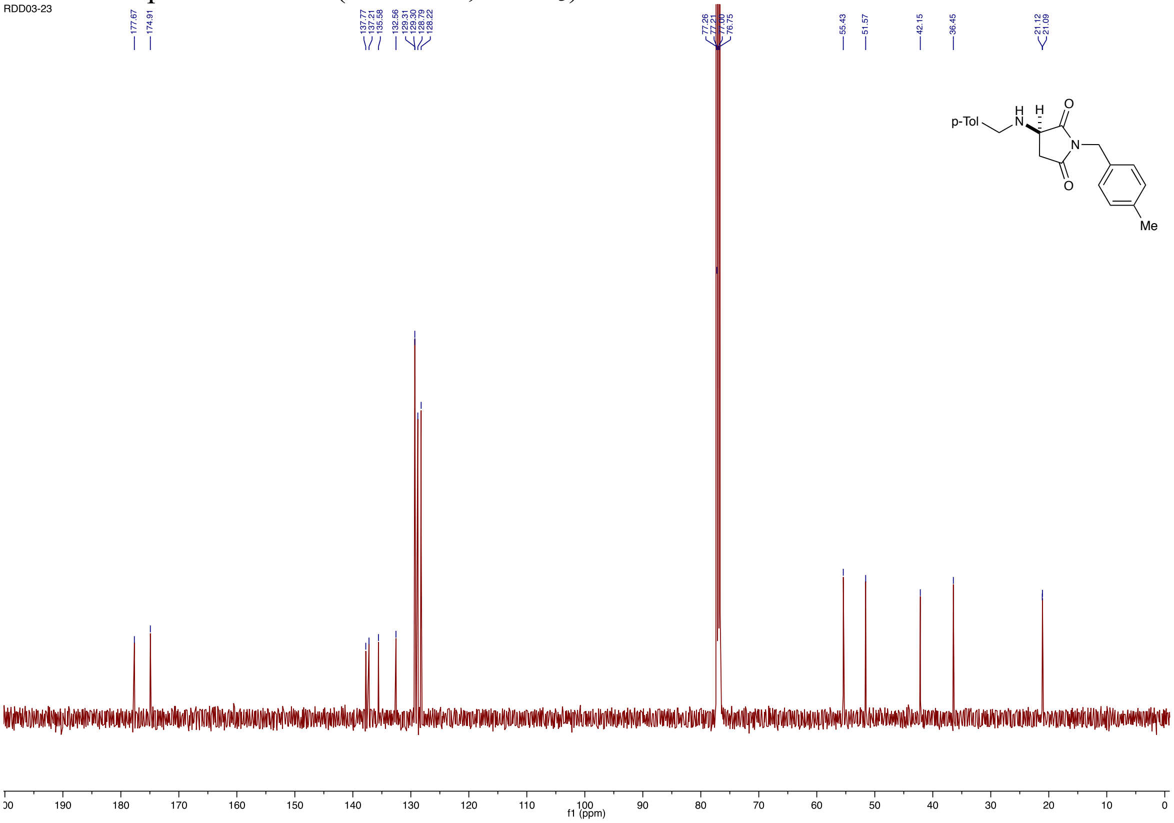

^{1}H NMR spectrum of **27** (500 MHz, $CDCl_3$)

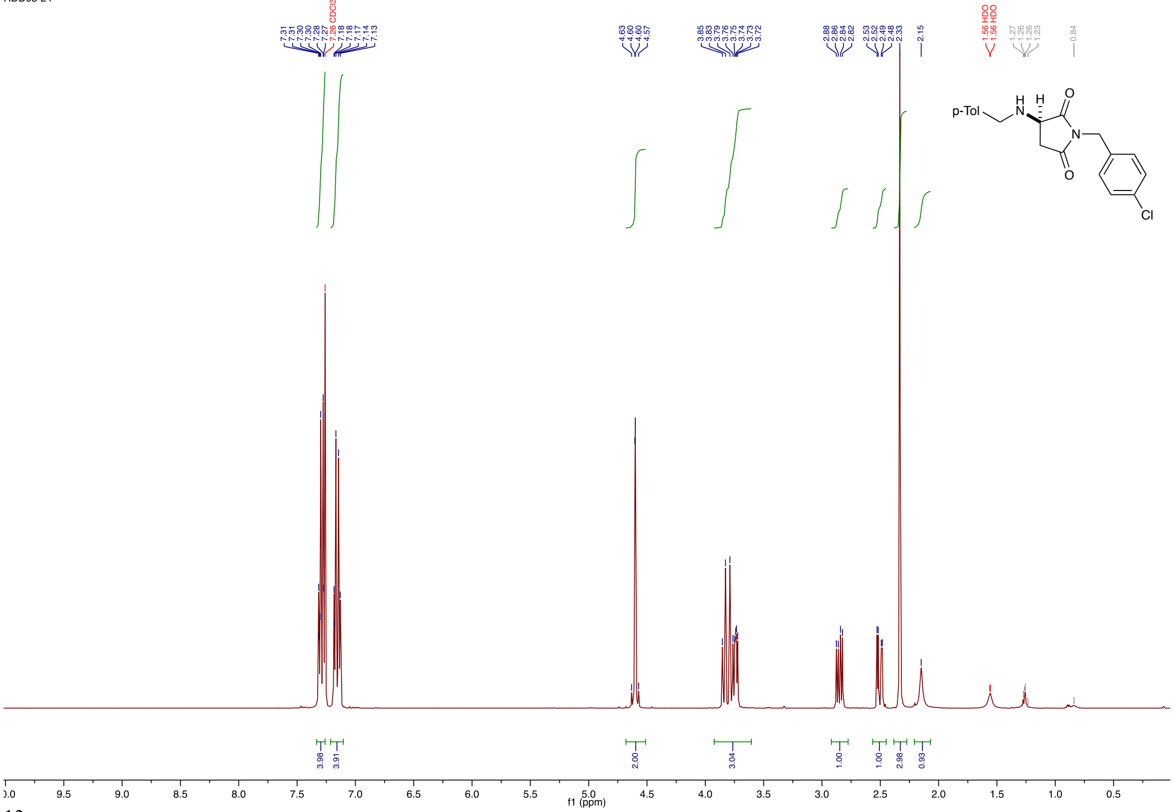

^{13}C NMR spectrum of **27** (126 MHz, $CDCl_3$)

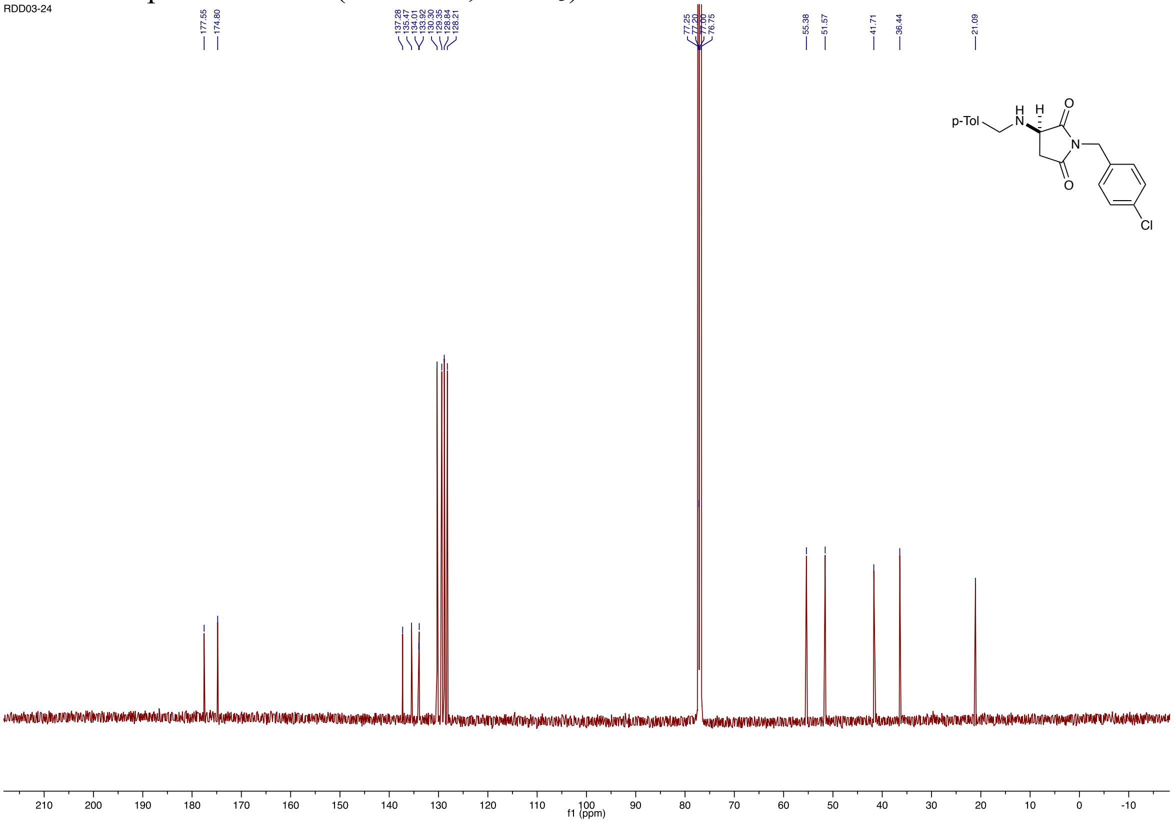

^{1}H NMR spectrum of **28** (500 MHz, $CDCl_3$)

BEU-DOW9-69_(24)

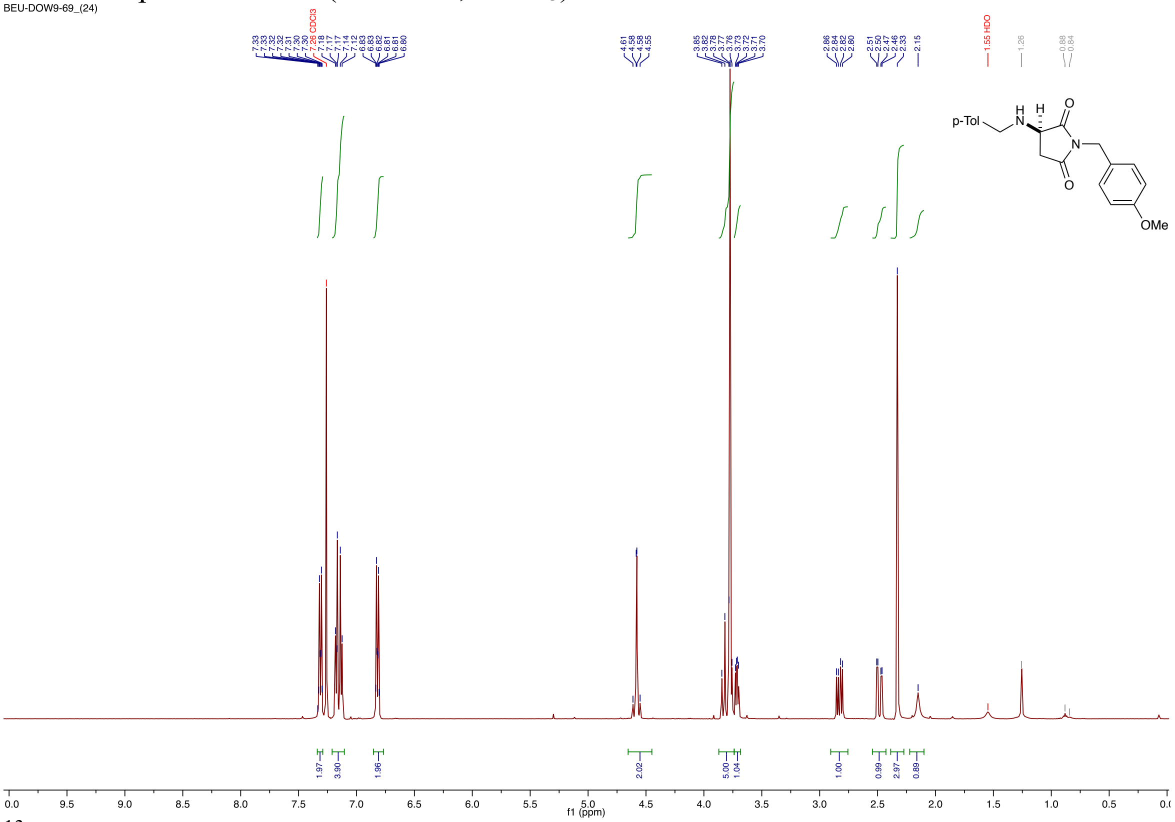

^{13}C NMR spectrum of **28** (126 MHz, $CDCl_3$)

BEU-DOW9-69_(24)

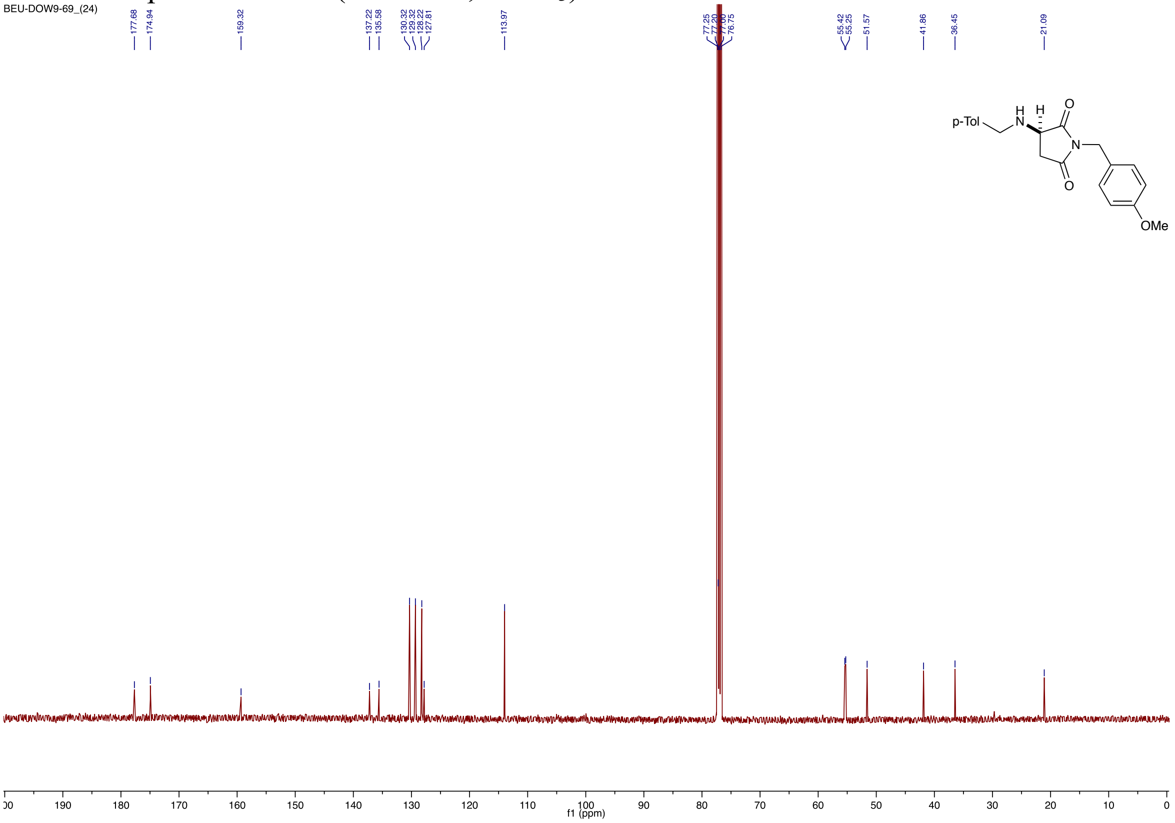

^{1}H NMR spectrum of **29** (500 MHz, $CDCl_3$)

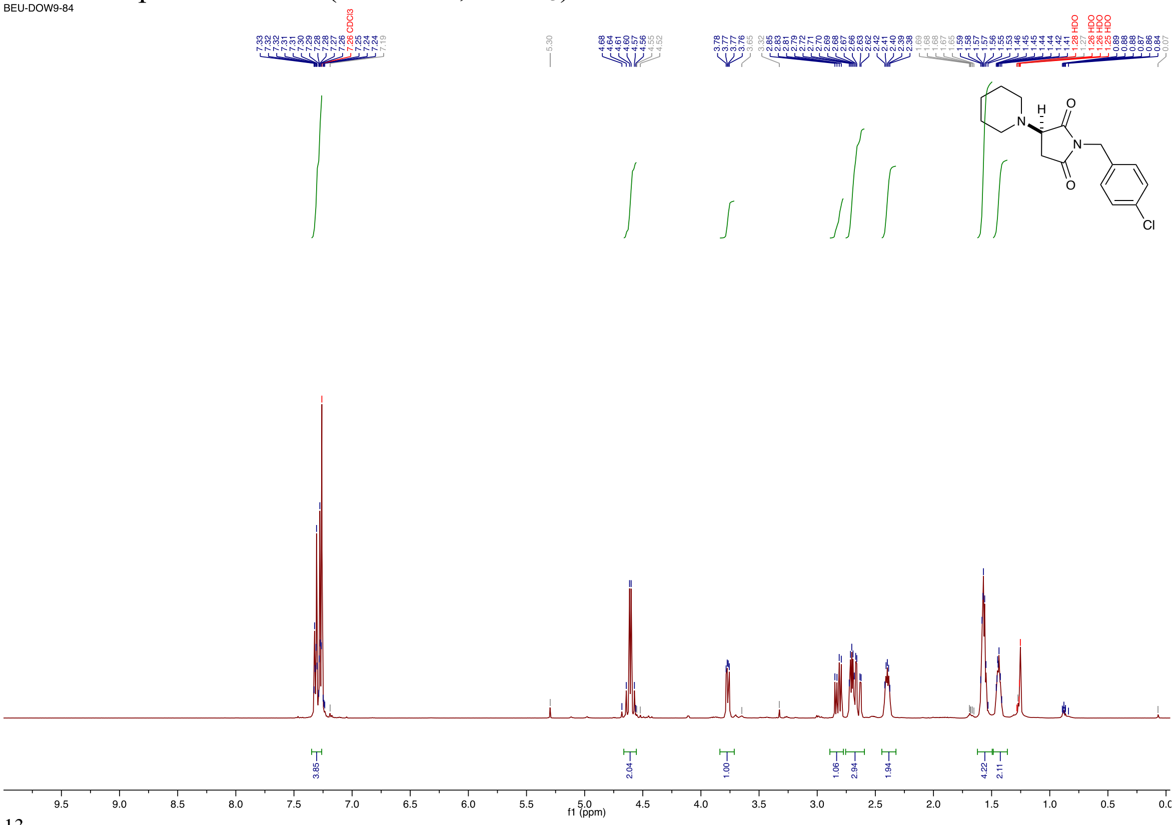

^{13}C NMR spectrum of **29** (126 MHz, $CDCl_3$)

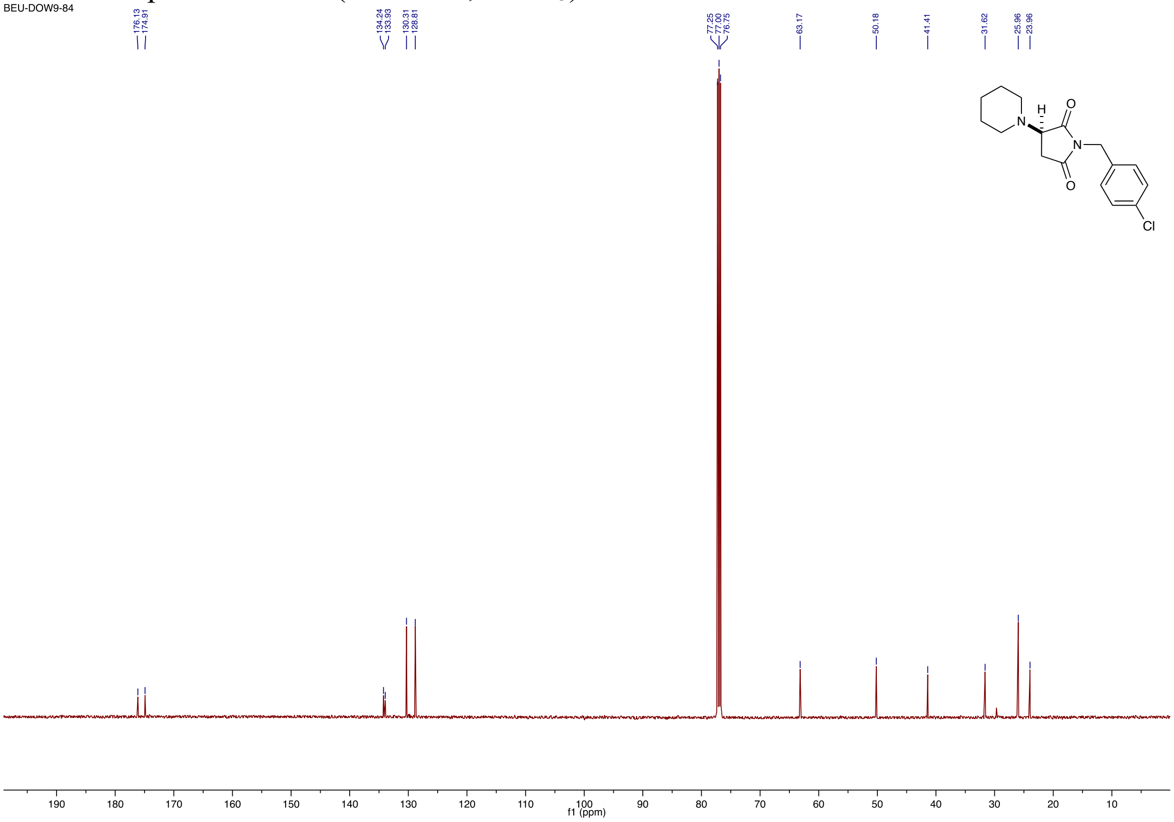

^{1}H NMR spectrum of **30** (500 MHz, $CDCl_3$)

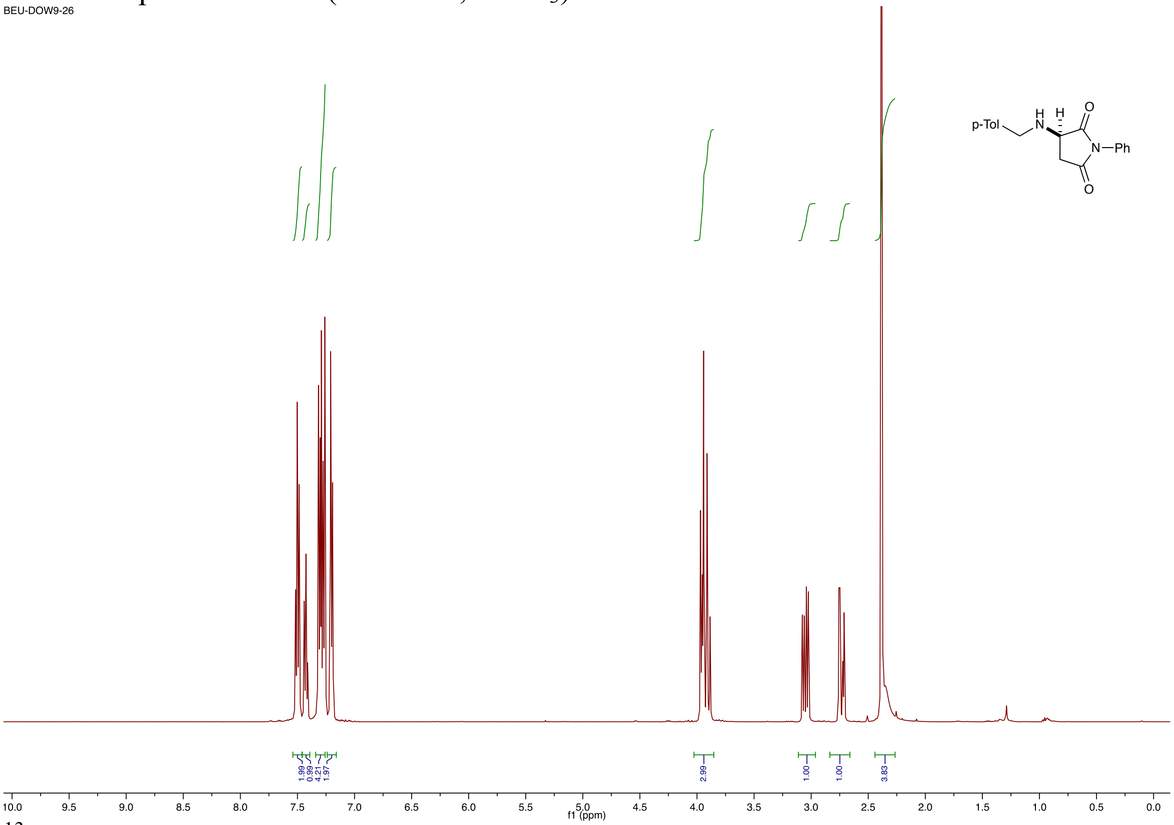

^{13}C NMR spectrum of **30** (126 MHz, $CDCl_3$)

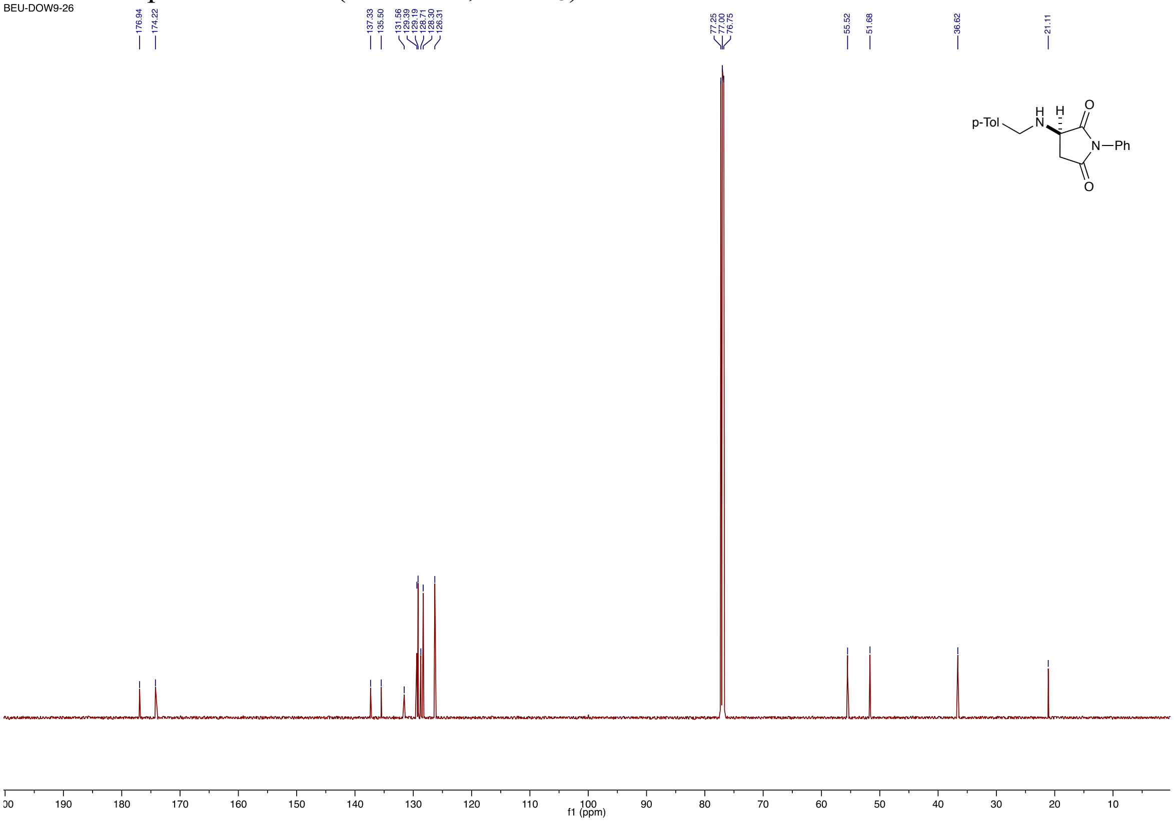

^{1}H NMR spectrum of **31** (500 MHz, $CDCl_3$)

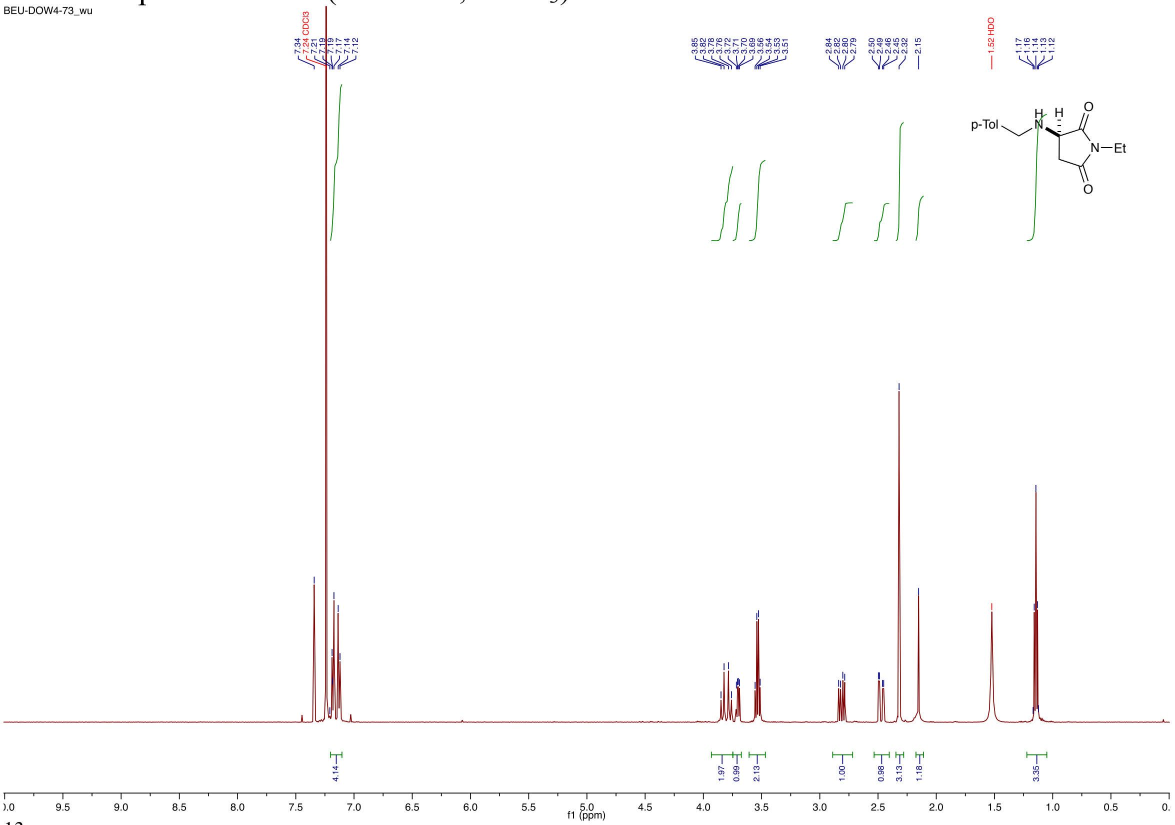

^{13}C NMR spectrum of **31** (126 MHz, $CDCl_3$)

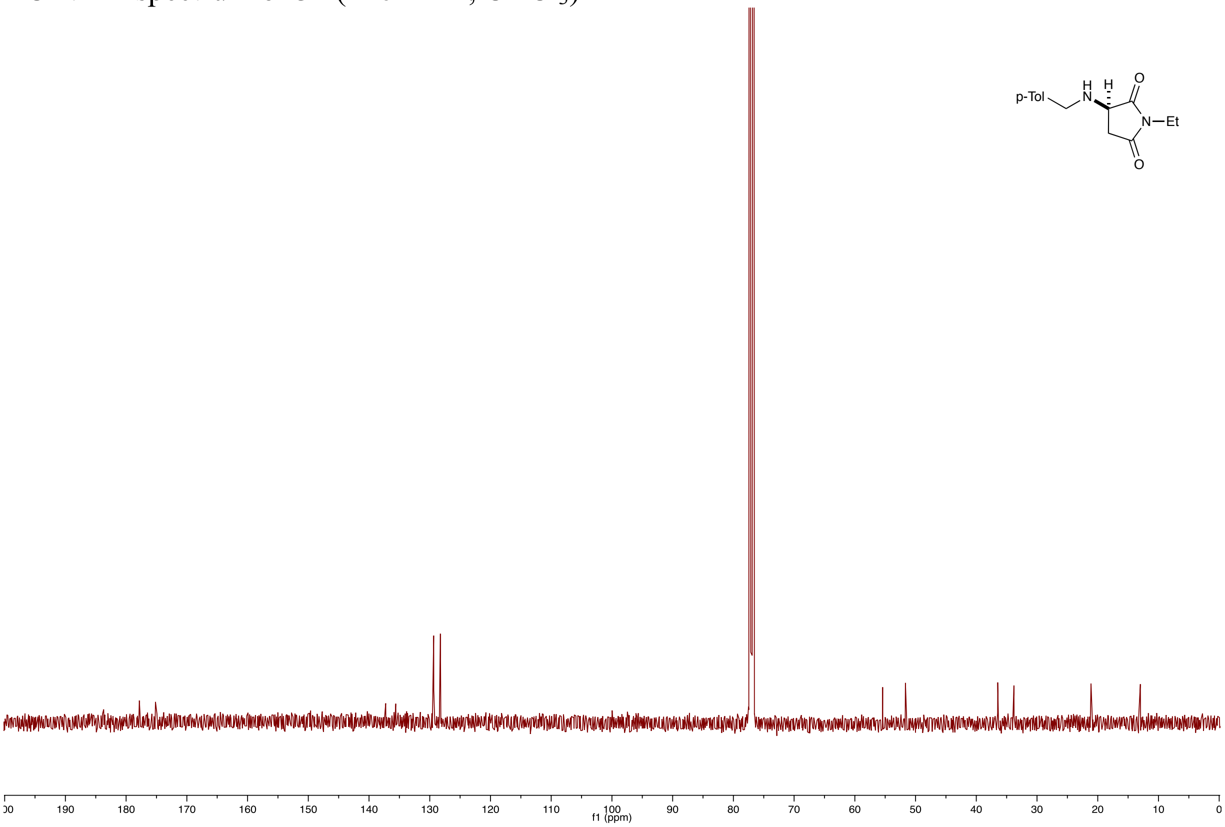

^{1}H NMR spectrum of **34** (500 MHz, $CDCl_3$)

BEU-DOW10-11

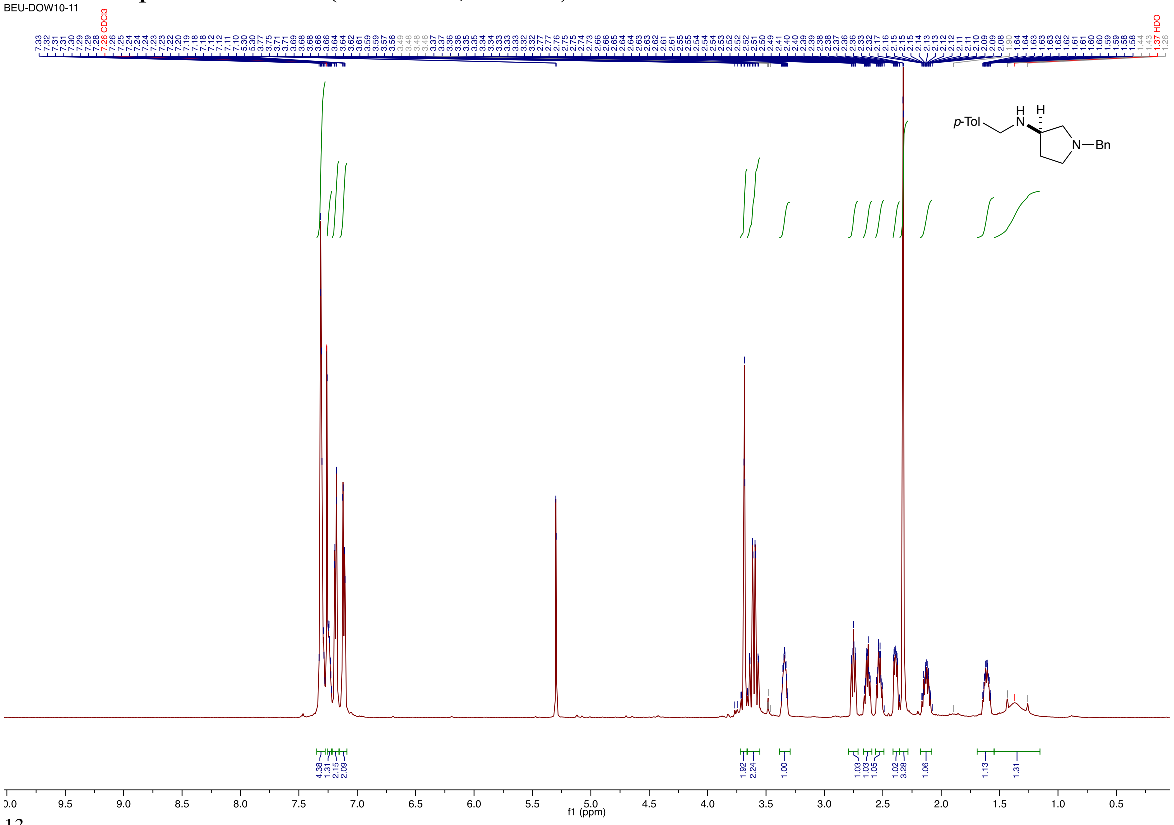

^{13}C NMR spectrum of **34** (126 MHz, $CDCl_3$)

BEU-DOW10-11

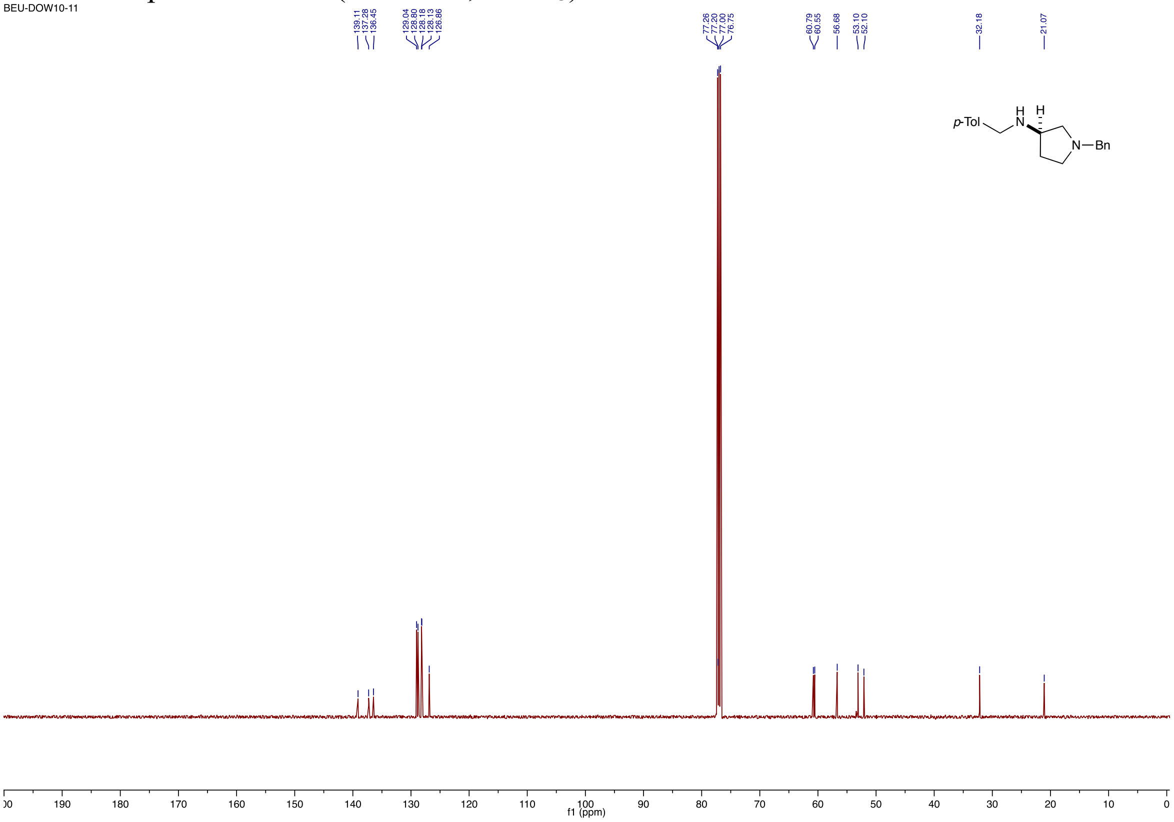

^{1}H NMR spectrum of **35** (500 MHz, $CDCl_3$)

BEU-DOW10-27_A

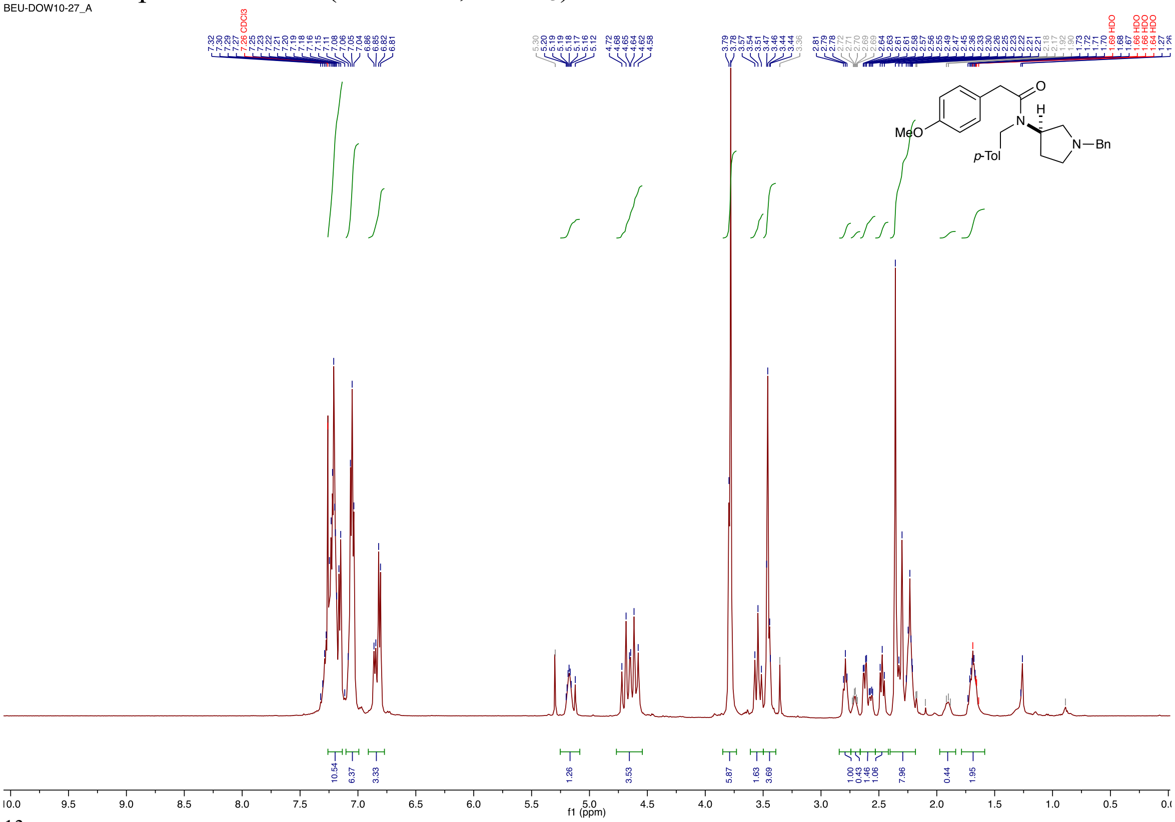

^{13}C NMR spectrum of **35** (126 MHz, $CDCl_3$)

BEU-DOW10-27_A

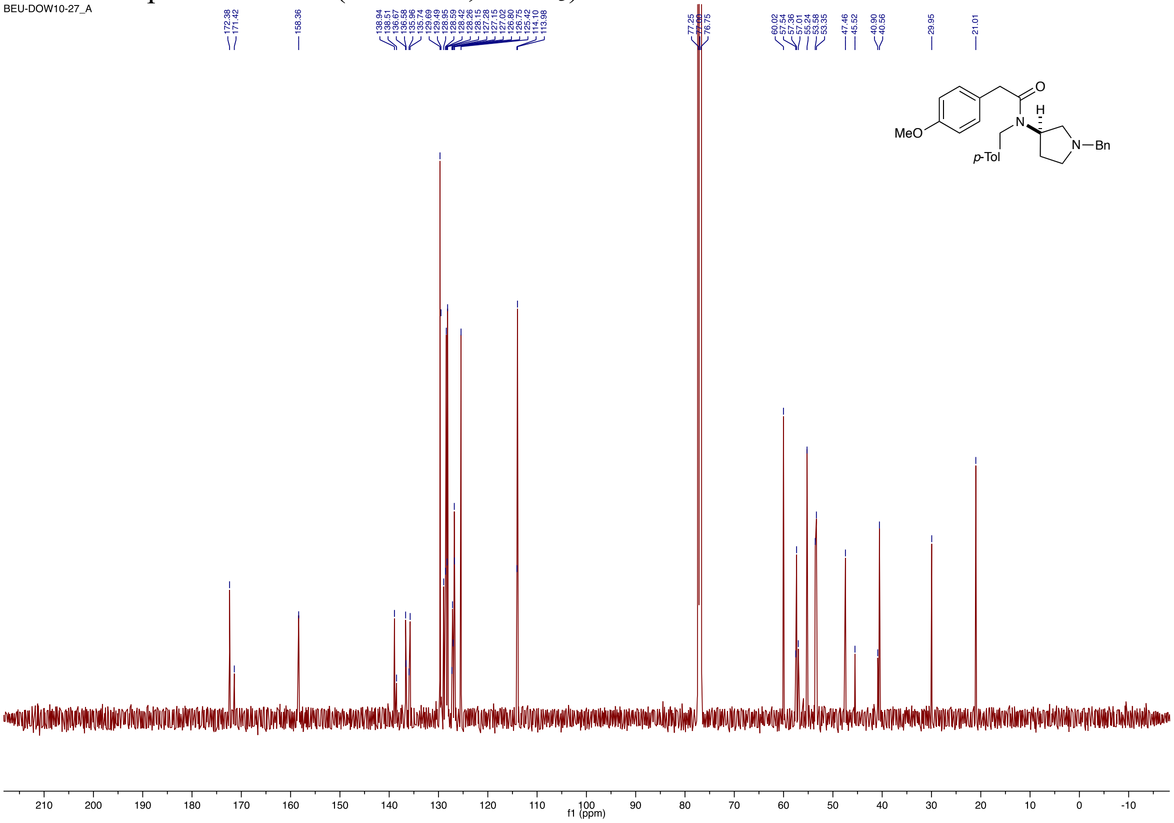

^{1}H NMR spectrum of **36** (500 MHz, $CDCl_3$)

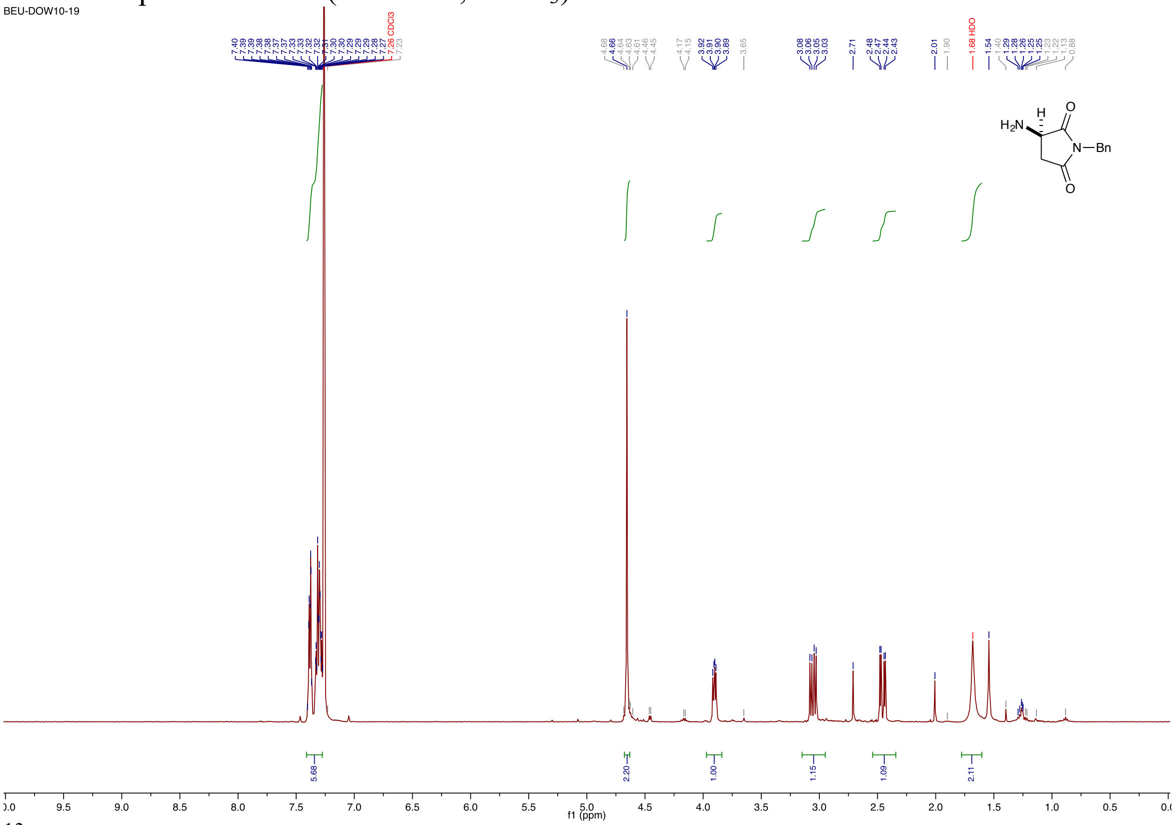

^{13}C NMR spectrum of **36** (126 MHz, $CDCl_3$)

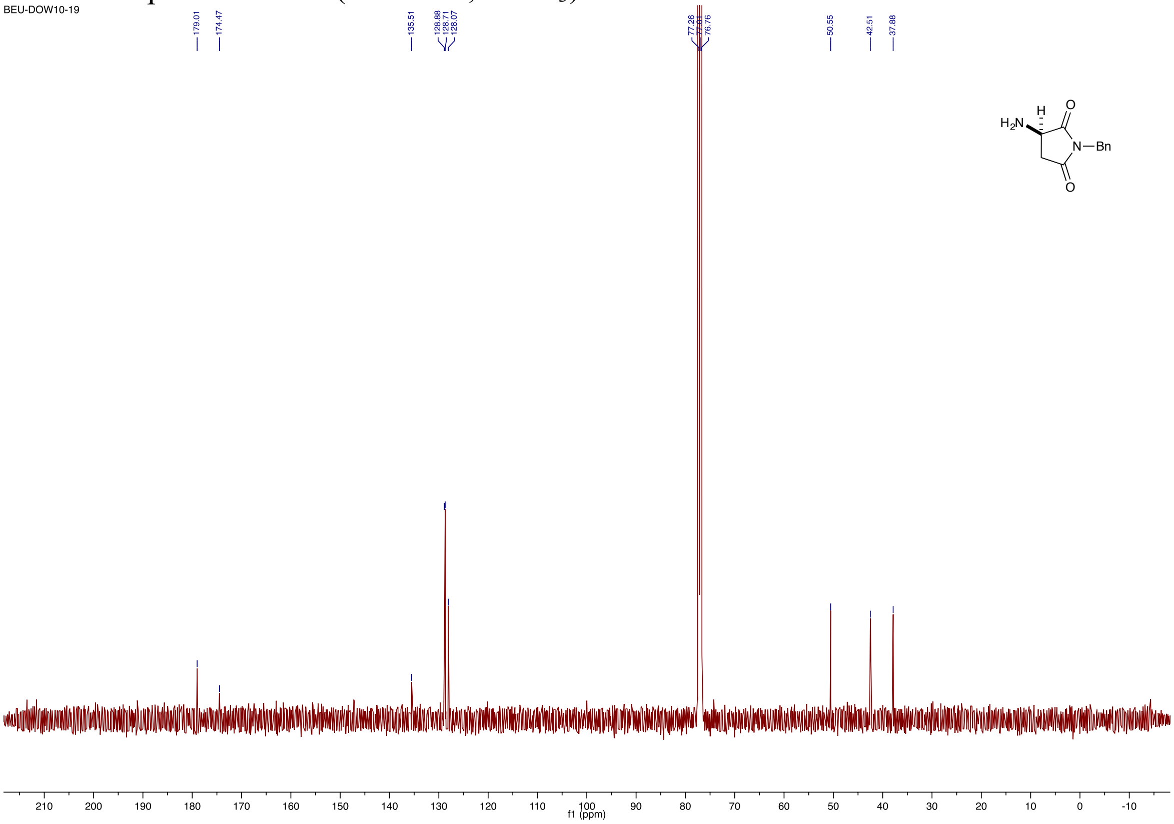

^{1}H NMR spectrum of ***trans*** **SI-1** (500 MHz, $CDCl_3$)

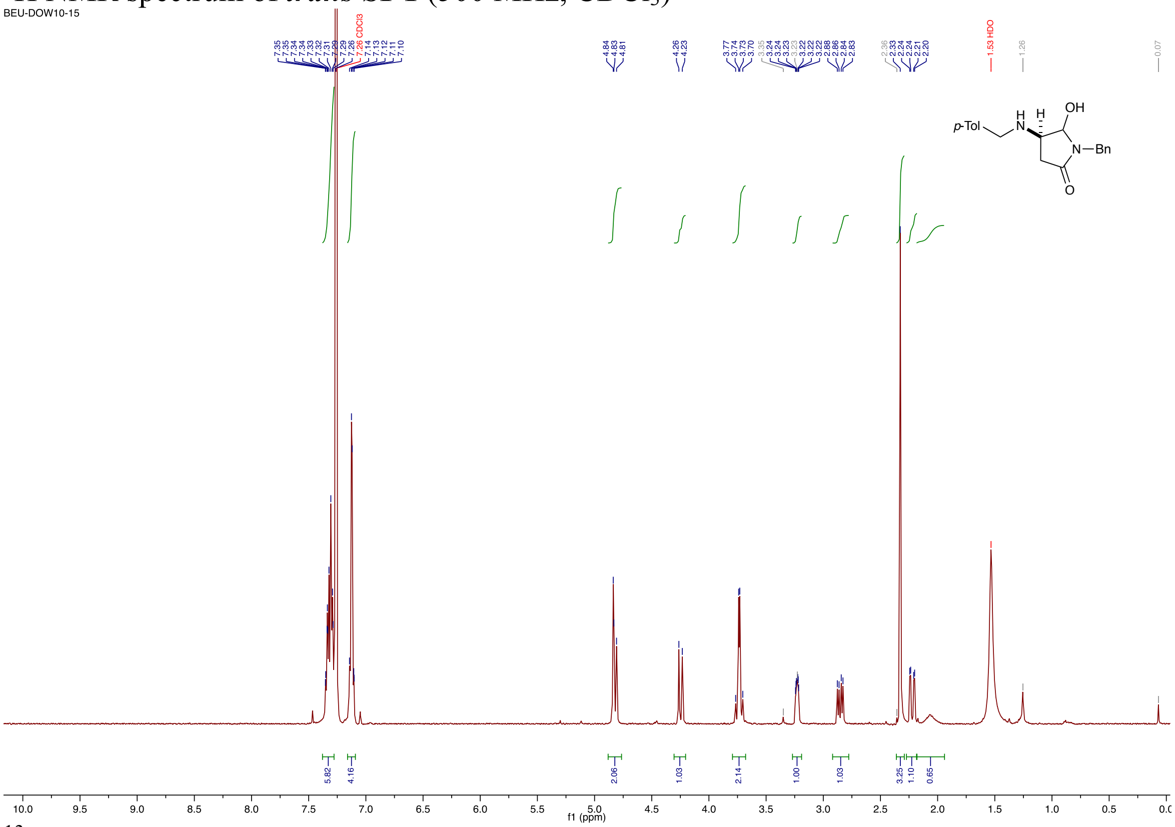

^{13}C NMR spectrum of ***trans*** **SI-1** (126 MHz, $CDCl_3$)

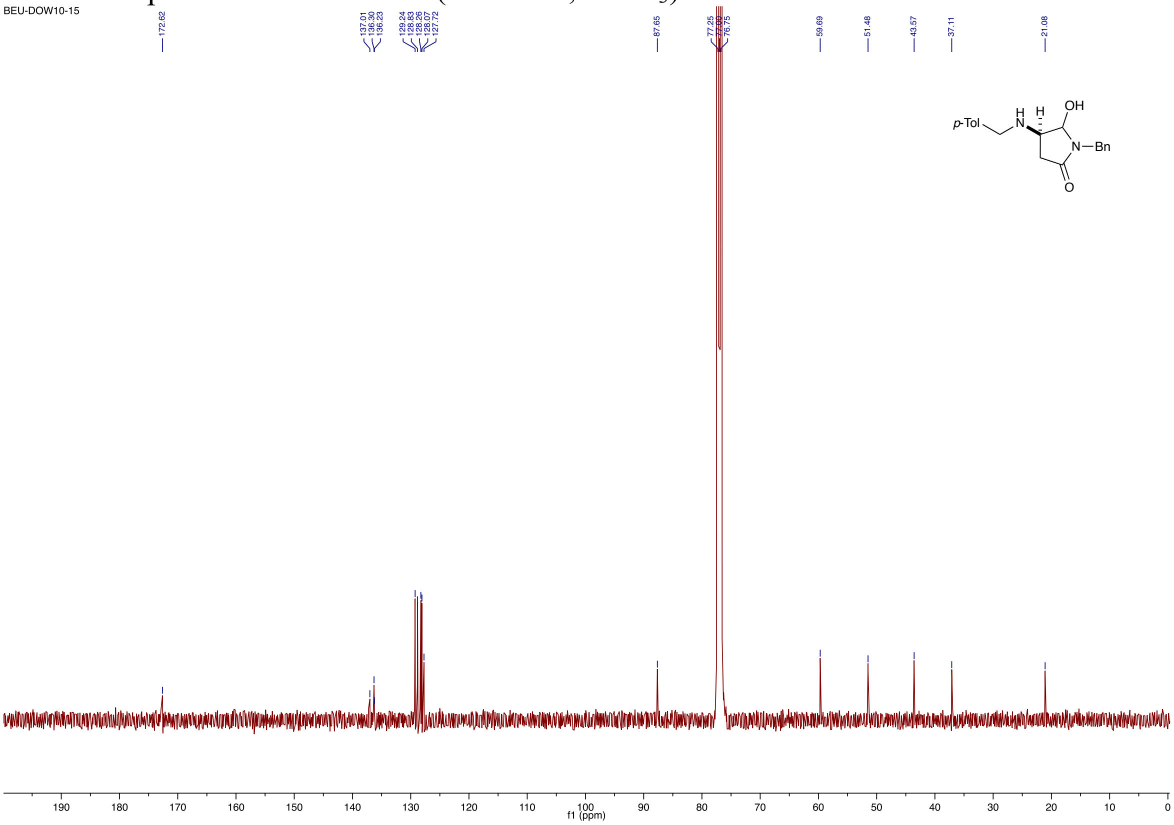

^{1}H NMR Spectrum of **37** (500 MHz, $CDCl_3$)

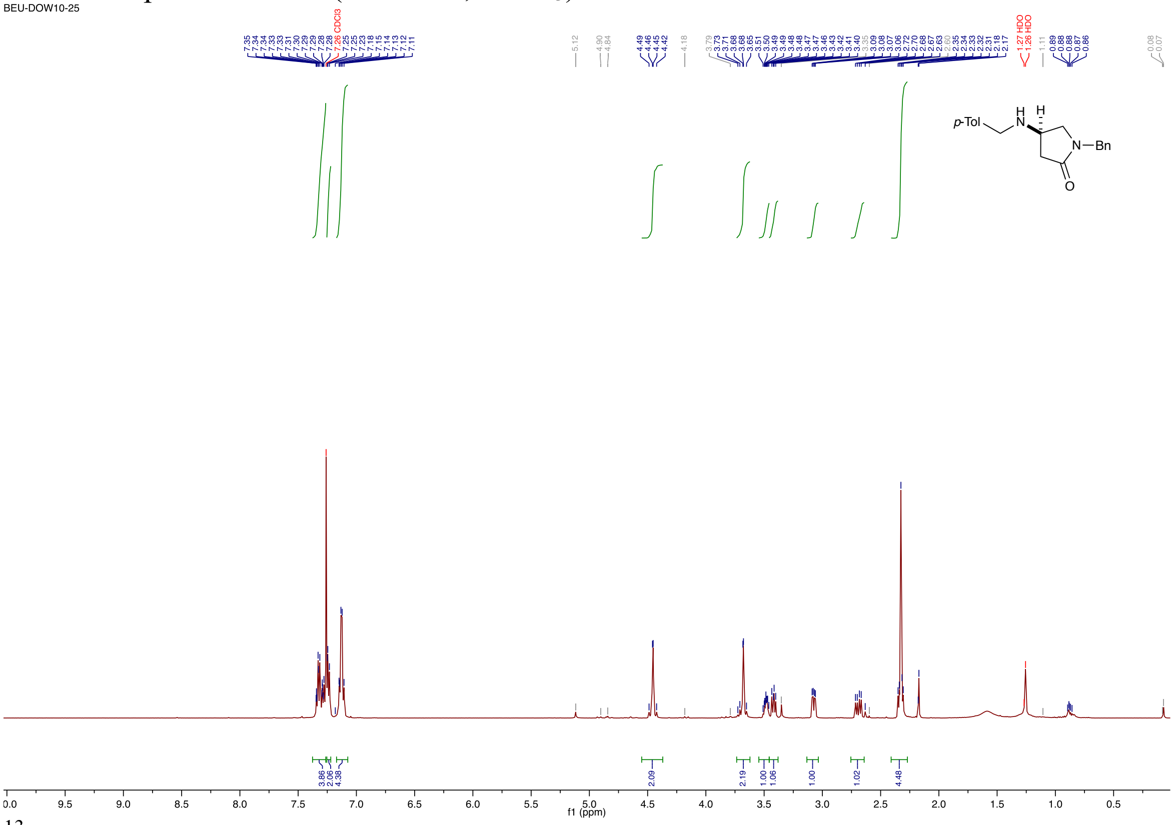

^{13}C NMR spectrum of **37** (126 MHz, $CDCl_3$)

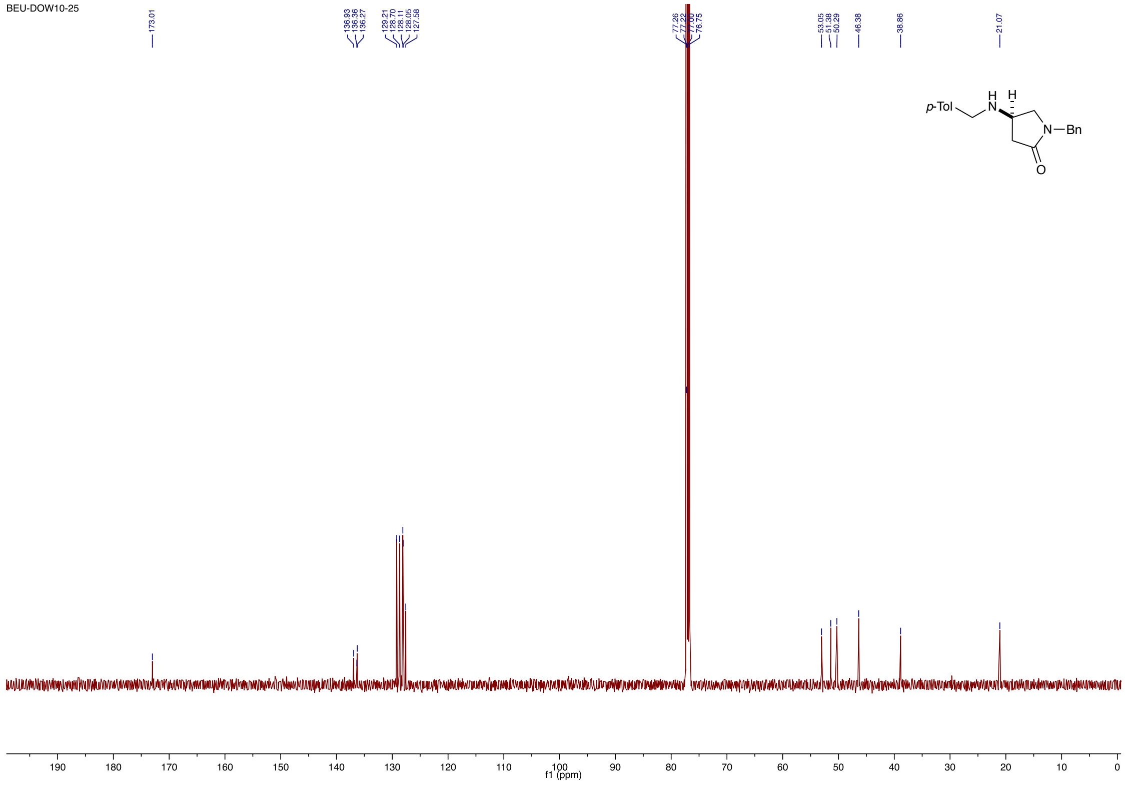

^{31}P NMR spectrum of Reaction Components (162 MHz, C_7D_8)

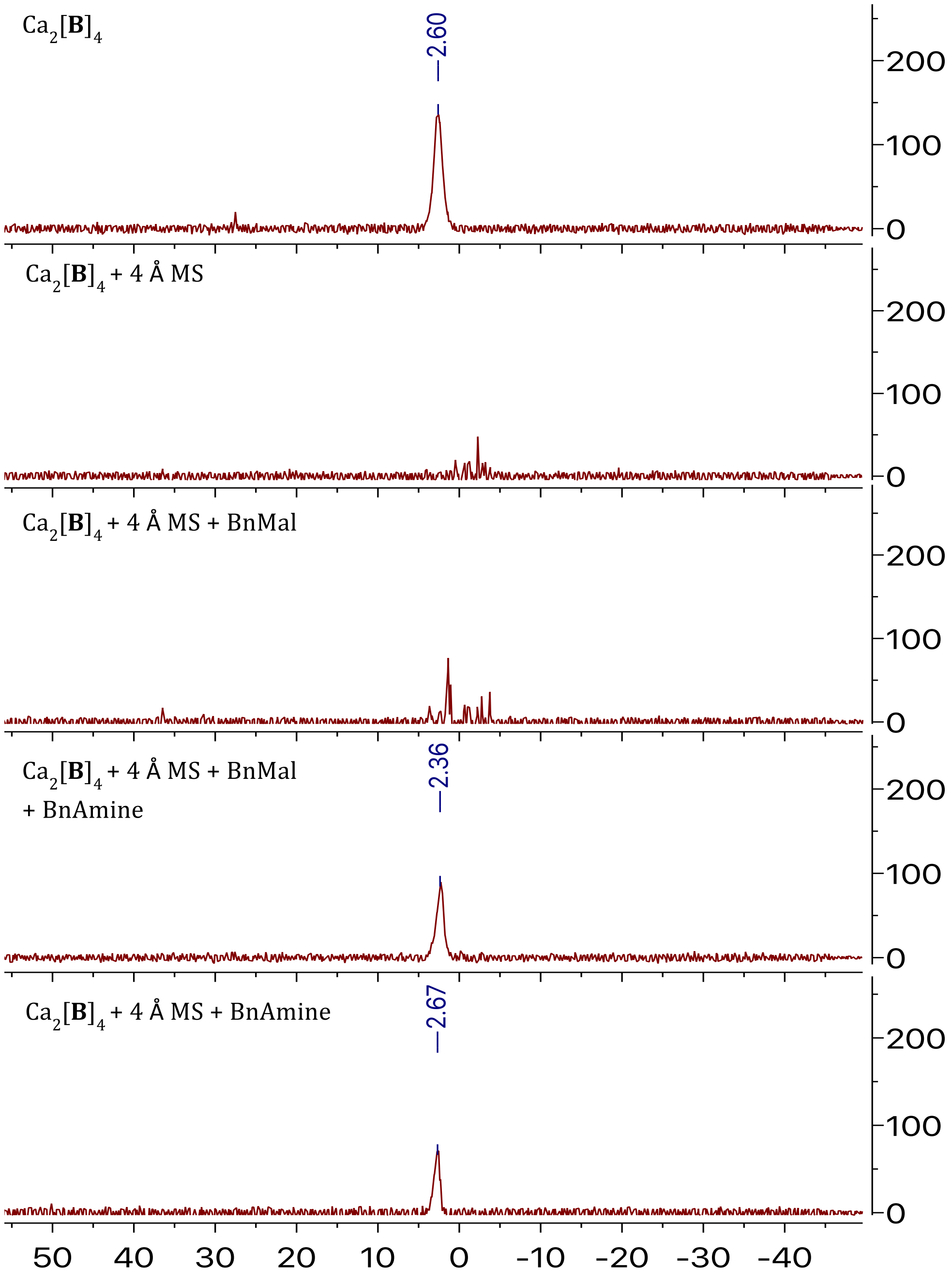

HPLC Traces of Racemic and Enantioenriched Compounds

Racemic and Enantioenriched **6**

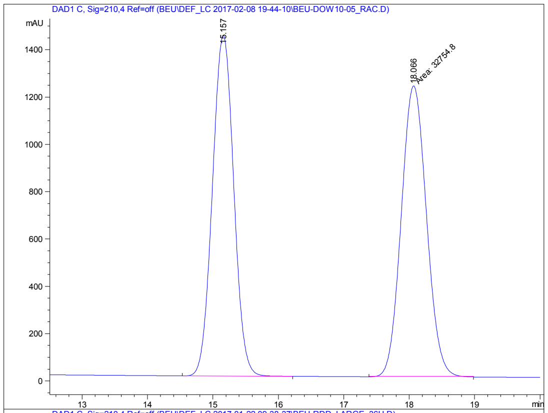

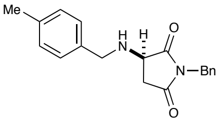

Signal 2: DAD1 C, Sig=210,4 Ref=off

Peak #	RetTime [min]	Type	Width [min]	Area [mAU*s]	Height [mAU]	Area %
1	15.157	BB	0.3562	3.25920e4	1438.98767	49.8755
2	18.066	MM	0.4447	3.27548e4	1227.72742	50.1245

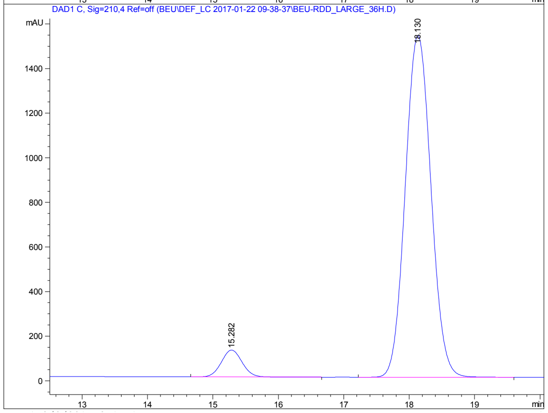

Signal 2: DAD1 C, Sig=210,4 Ref=off

Peak #	RetTime [min]	Type	Width [min]	Area [mAU*s]	Height [mAU]	Area %
1	15.282	BB	0.3438	2668.22388	120.72897	6.0008
2	18.130	BB	0.4282	4.17964e4	1532.23572	93.9992

Racemic and Enantioenriched **6** after recrystalization.

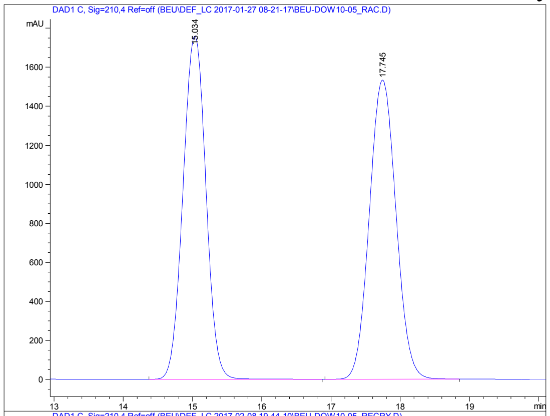

recrystallized

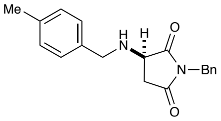

Signal 2: DAD1 C, Sig=210,4 Ref=off

Peak #	RetTime [min]	Type	Width [min]	Area [mAU*s]	Height [mAU]	Area %
1	15.034	BB	0.3636	4.03271e4	1758.21216	49.6235
2	17.745	BB	0.4216	4.09390e4	1532.84058	50.3765

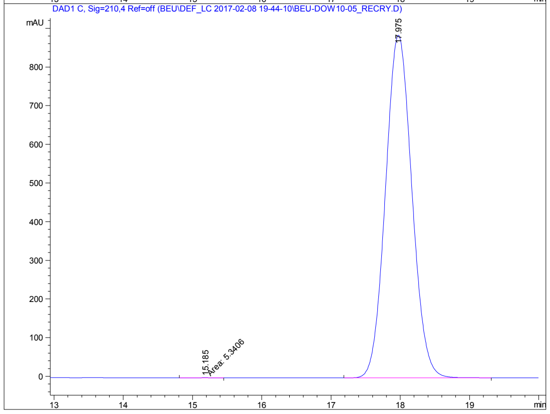

Signal 2: DAD1 C, Sig=210,4 Ref=off

Peak #	RetTime [min]	Type	Width [min]	Area [mAU*s]	Height [mAU]	Area %
1	15.185	MM	0.3149	5.34060	2.82687e-1	0.0228
2	17.975	BB	0.4127	2.34513e4	886.38623	99.9772

Racemic and Enantioenriched **6** after Ca[**B**]$_2$ recovery

Recovered catalyst

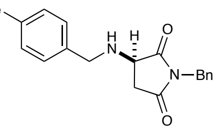

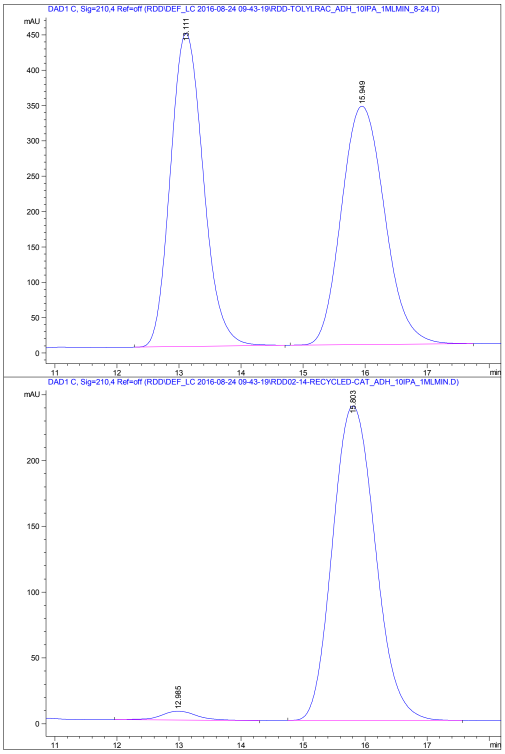

Recovered catalyst was validated on the initial screening scale 0.02 mmol (data matches table 1 entry 7).

Signal 2: DAD1 C, Sig=210,4 Ref=off

Peak #	RetTime [min]	Type	Width [min]	Area [mAU*s]	Height [mAU]	Area %
1	13.111	BB	0.5815	1.63209e4	443.31677	50.0216
2	15.949	BB	0.7681	1.63068e4	337.26083	49.9784

Signal 2: DAD1 C, Sig=210,4 Ref=off

Peak #	RetTime [min]	Type	Width [min]	Area [mAU*s]	Height [mAU]	Area %
1	12.985	BB	0.6044	269.39487	6.73893	2.3068
2	15.803	BB	0.7572	1.14089e4	238.89505	97.6932

*retention times are slightly earlier due to a different AD-H column

Racemic and Enantioenriched **7**

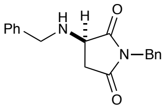

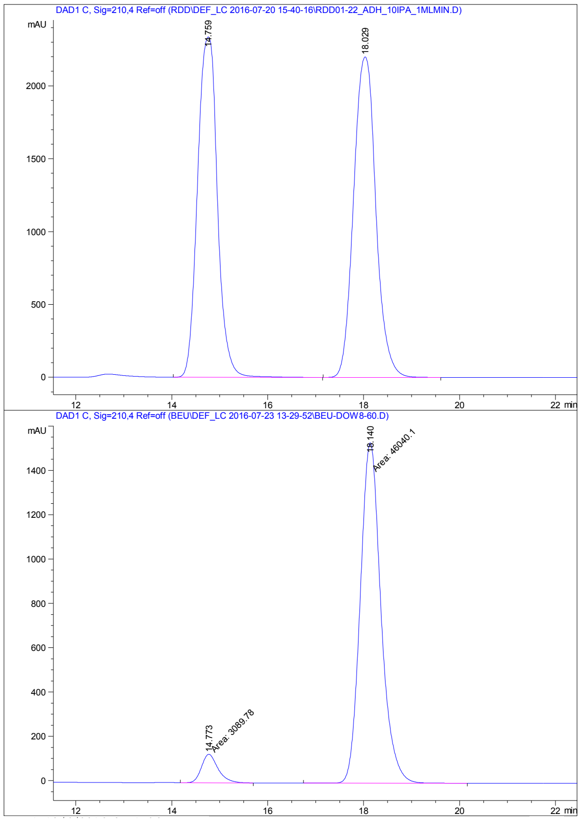

Signal 2: DAD1 C, Sig=210,4 Ref=off

Peak #	RetTime [min]	Type	Width [min]	Area [mAU*s]	Height [mAU]	Area %
1	14.759	VB	0.4494	6.71655e4	2336.09424	48.6931
2	18.029	BB	0.5034	7.07709e4	2199.48340	51.3069

Signal 2: DAD1 C, Sig=210,4 Ref=off

Peak #	RetTime [min]	Type	Width [min]	Area [mAU*s]	Height [mAU]	Area %
1	14.773	MM	0.4003	3089.77832	128.65573	6.2890
2	18.140	MM	0.4997	4.60401e4	1535.71973	93.7110

Racemic and Enantioenriched **8**

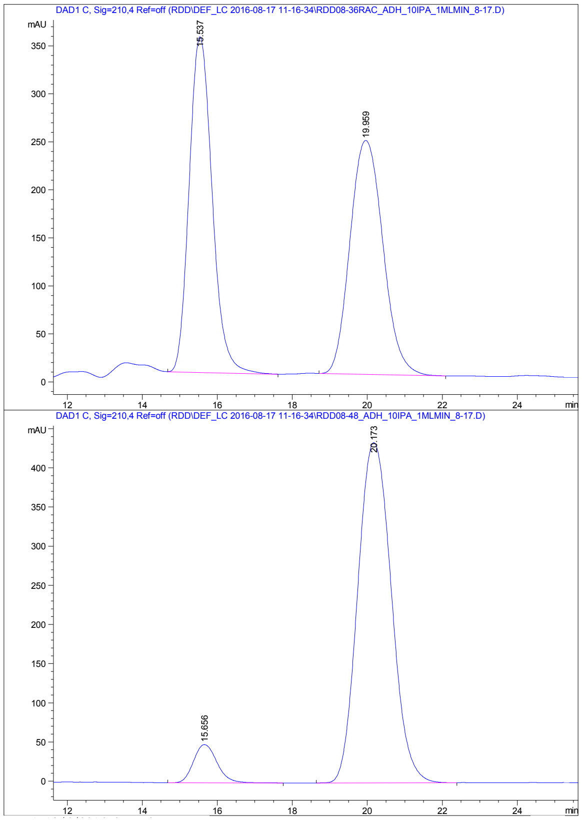

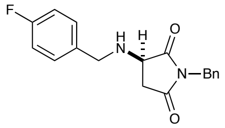

Signal 2: DAD1 C, Sig=210,4 Ref=off

Peak #	RetTime [min]	Type	Width [min]	Area [mAU*s]	Height [mAU]	Area %
1	15.537	BB	0.6708	1.48897e4	349.64786	49.9823
2	19.959	BB	0.9712	1.49003e4	243.72934	50.0177

Peak #	RetTime [min]	Type	Width [min]	Area [mAU*s]	Height [mAU]	Area %
1	15.656	BB	0.6914	2142.71191	48.88052	7.3633
2	20.173	BB	0.9980	2.69571e4	434.41962	92.6367

Racemic and Enantioenriched **9**

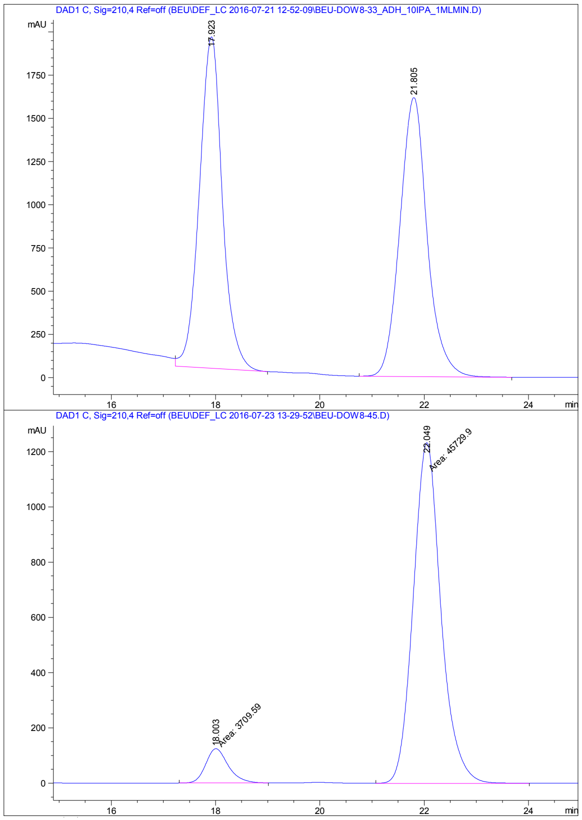

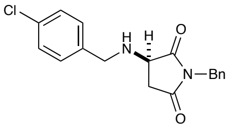

Signal 2: DAD1 C, Sig=210,4 Ref=off

Peak #	RetTime [min]	Type	Width [min]	Area [mAU*s]	Height [mAU]	Area %
1	17.923	VB	0.4680	5.88496e4	1917.96606	50.0455
2	21.805	BB	0.5564	5.87425e4	1614.99658	49.9545

Signal 2: DAD1 C, Sig=210,4 Ref=off

Peak #	RetTime [min]	Type	Width [min]	Area [mAU*s]	Height [mAU]	Area %
1	18.003	MM	0.4985	3709.58740	124.02554	7.5033
2	22.049	MM	0.6177	4.57299e4	1233.80652	92.4967

Racemic and Enantioenriched **10**

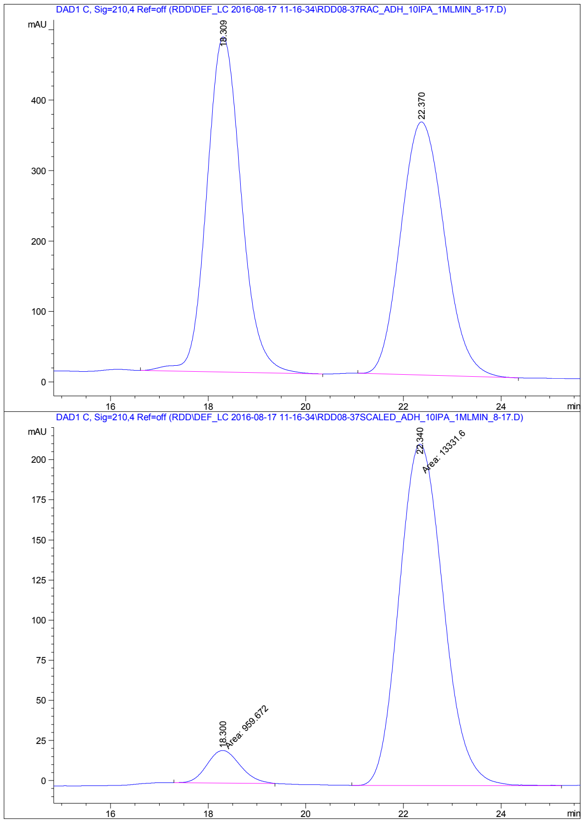

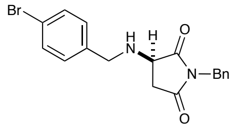

Peak #	RetTime [min]	Type	Width [min]	Area [mAU*s]	Height [mAU]	Area %
1	18.309	BB	0.7636	2.32335e4	475.85065	51.0318
2	22.370	BB	0.9897	2.22940e4	359.37985	48.9682

Peak #	RetTime [min]	Type	Width [min]	Area [mAU*s]	Height [mAU]	Area %
1	18.297	BB	0.7516	1010.53448	20.62597	7.0672
2	22.340	BB	0.9971	1.32884e4	212.62537	92.9328

Racemic and Enantioenriched **11**

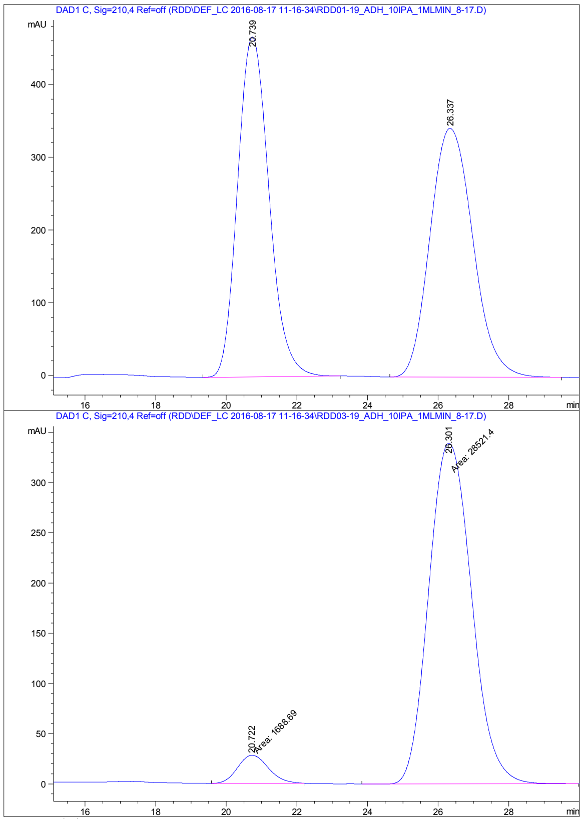

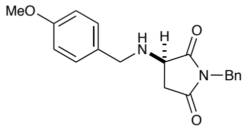

Peak #	RetTime [min]	Type	Width [min]	Area [mAU*s]	Height [mAU]	Area %
1	20.739	BB	0.9739	2.89144e4	467.28027	50.0415
2	26.337	BB	1.3426	2.88665e4	342.02682	49.9585

Peak #	RetTime [min]	Type	Width [min]	Area [mAU*s]	Height [mAU]	Area %
1	20.722	BB	0.9347	1730.00598	28.23901	5.7214
2	26.301	BB	1.3306	2.85073e4	339.16788	94.2786

Racemic and Enantioenriched **12**

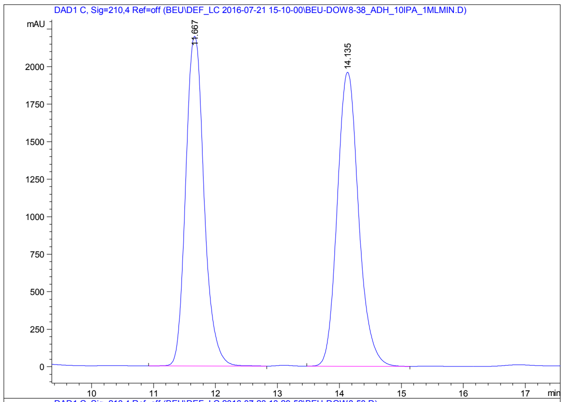

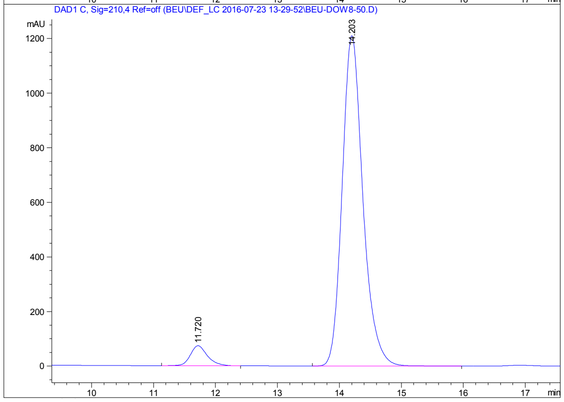

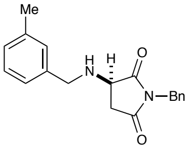

Signal 2: DAD1 C, Sig=210,4 Ref=off

Peak #	RetTime [min]	Type	Width [min]	Area [mAU*s]	Height [mAU]	Area %
1	11.667	BV	0.3187	4.53486e4	2198.07178	49.0752
2	14.135	BB	0.3701	4.70579e4	1960.18848	50.9248

Signal 2: DAD1 C, Sig=210,4 Ref=off

Peak #	RetTime [min]	Type	Width [min]	Area [mAU*s]	Height [mAU]	Area %
1	11.720	BB	0.2910	1419.42139	73.61469	4.8069
2	14.203	BB	0.3550	2.81096e4	1210.07690	95.1931

Racemic and Enantioenriched **13**

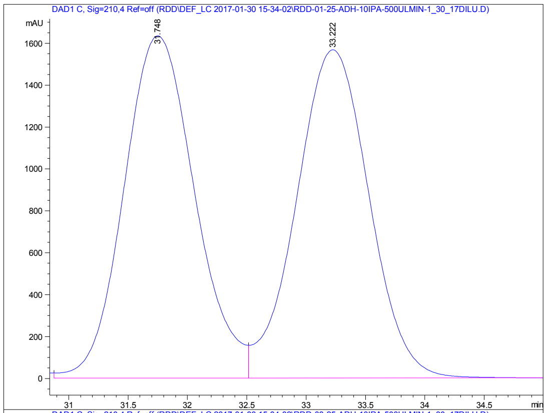

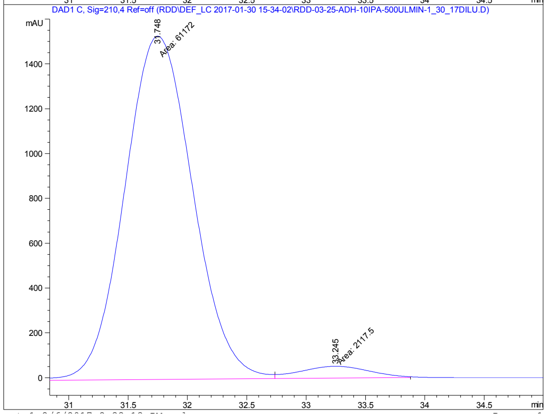

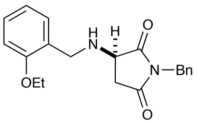

Signal 2: DAD1 C, Sig=210,4 Ref=off

Peak #	RetTime [min]	Type	Width [min]	Area [mAU*s]	Height [mAU]	Area %
1	31.748	VV	0.6436	6.77075e4	1633.26892	50.4118
2	33.222	VBA	0.6602	6.66012e4	1565.90356	49.5882

Signal 2: DAD1 C, Sig=210,4 Ref=off

Peak #	RetTime [min]	Type	Width [min]	Area [mAU*s]	Height [mAU]	Area %
1	31.748	MF	0.6652	6.11720e4	1532.58704	96.6543
2	33.245	FM	0.6720	2117.50317	52.51970	3.3457

Racemic and Enantioenriched **14**

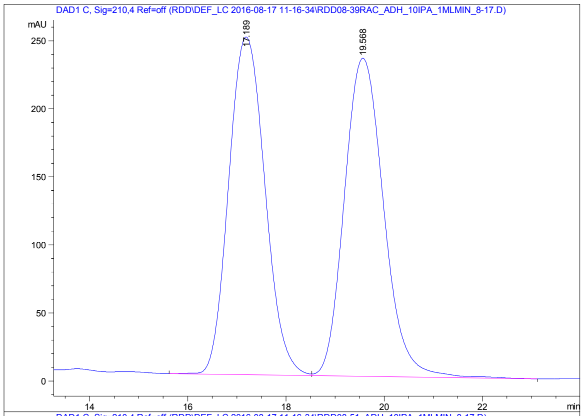

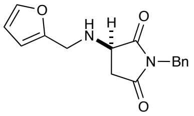

Signal 2: DAD1 C, Sig=210,4 Ref=off

Peak #	RetTime [min]	Type	Width [min]	Area [mAU*s]	Height [mAU]	Area %
1	17.189	BV	0.8192	1.27507e4	248.27483	49.4372
2	19.568	VB	0.8726	1.30410e4	233.86095	50.5628

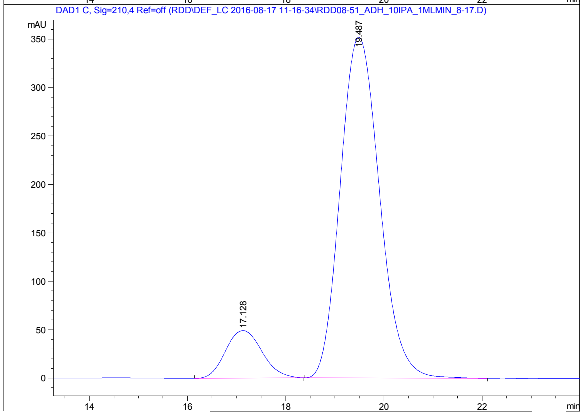

Peak #	RetTime [min]	Type	Width [min]	Area [mAU*s]	Height [mAU]	Area %
1	17.128	BB	0.8174	2530.27808	49.08475	11.4596
2	19.487	BB	0.8759	1.95498e4	352.04941	88.5404

Racemic and Enantioenriched **15**

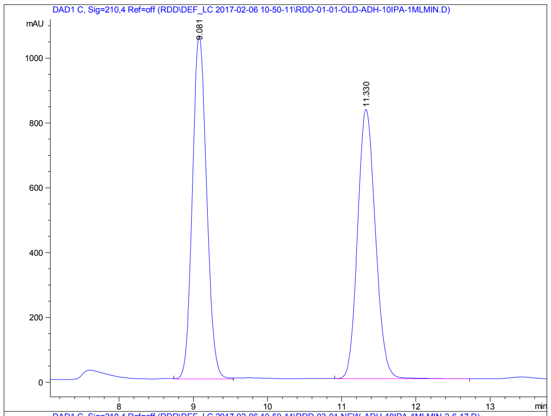

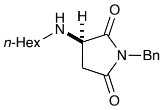

Signal 2: DAD1 C, Sig=210,4 Ref=off

Peak #	RetTime [min]	Type	Width [min]	Area [mAU*s]	Height [mAU]	Area %
1	9.081	VV	0.2081	1.41470e4	1057.62500	50.4317
2	11.330	VB	0.2612	1.39049e4	831.24164	49.5683

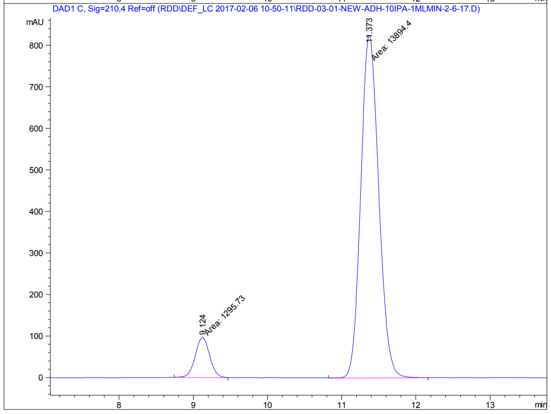

Signal 2: DAD1 C, Sig=210,4 Ref=off

Peak #	RetTime [min]	Type	Width [min]	Area [mAU*s]	Height [mAU]	Area %
1	9.124	MM	0.2241	1295.73181	96.35020	8.5301
2	11.373	MM	0.2804	1.38944e4	825.96472	91.4699

Racemic and Enantioenriched **16**

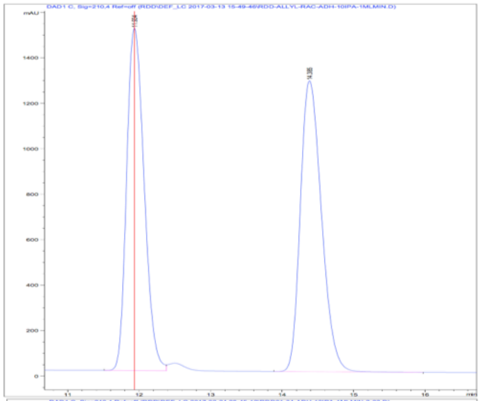

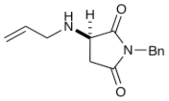

Signal 2: DAD1 C, Sig=210,4 Ref=off

Peak #	RetTime [min]	Type	Width [min]	Area [mAU*s]	Height [mAU]	Area %
1	11.934	BV	0.2689	2.56631e4	1505.54968	49.3905
2	14.385	BB	0.3217	2.62964e4	1279.97986	50.6095

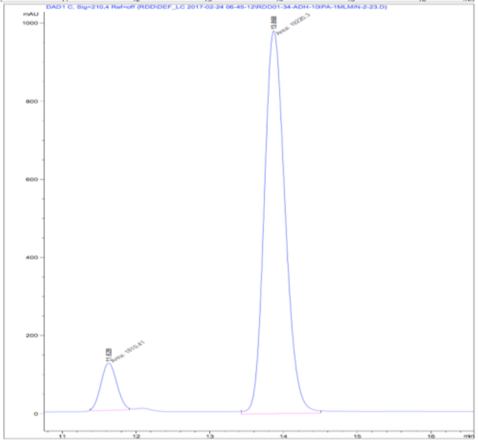

Signal 2: DAD1 C, Sig=210,4 Ref=off

Peak #	RetTime [min]	Type	Width [min]	Area [mAU*s]	Height [mAU]	Area %
1	11.628	MM	0.2494	1810.41260	120.98617	8.6084
2	13.868	MM	0.3267	1.92203e4	980.60352	91.3916

Racemic and Enantioenriched **17**

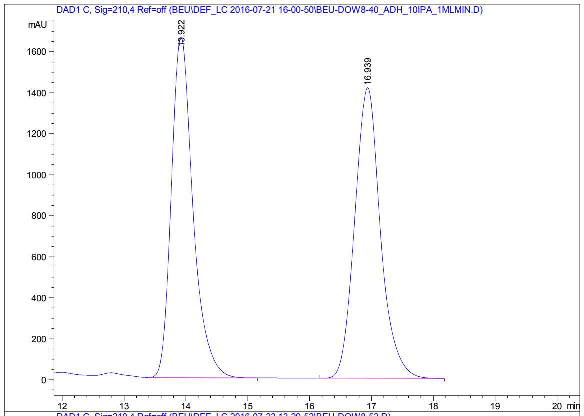

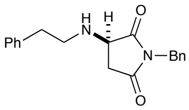

Signal 2: DAD1 C, Sig=210,4 Ref=off

Peak #	RetTime [min]	Type	Width [min]	Area [mAU*s]	Height [mAU]	Area %
1	13.922	BB	0.3703	4.05253e4	1662.80652	49.7130
2	16.939	BBA	0.4413	4.09932e4	1417.85779	50.2870

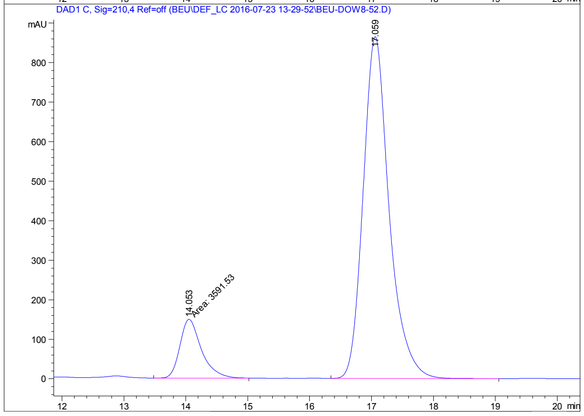

Signal 2: DAD1 C, Sig=210,4 Ref=off

Peak #	RetTime [min]	Type	Width [min]	Area [mAU*s]	Height [mAU]	Area %
1	14.053	MM	0.4021	3591.53125	148.85593	12.4573
2	17.059	BB	0.4405	2.52393e4	864.77283	87.5427

Racemic and Enantioenriched **18**

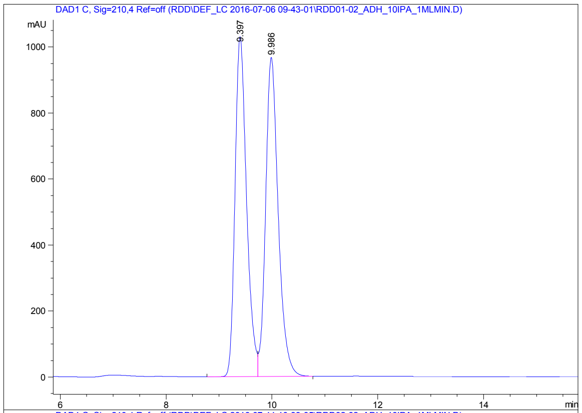

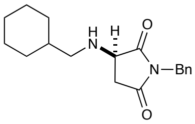

Signal 2: DAD1 C, Sig=210,4 Ref=off

Peak #	RetTime [min]	Type	Width [min]	Area [mAU*s]	Height [mAU]	Area %
1	9.397	BV	0.2308	1.56068e4	1030.02271	49.3709
2	9.986	VB	0.2511	1.60045e4	966.76324	50.6291

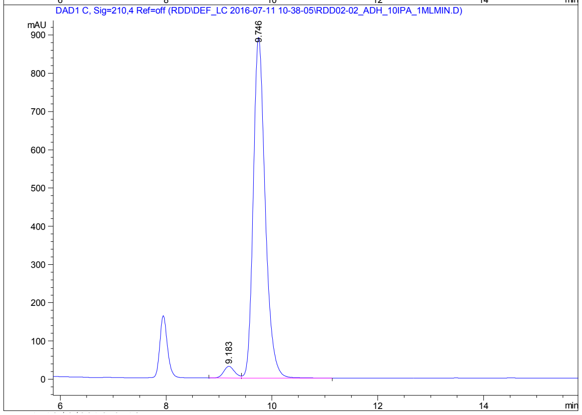

Signal 2: DAD1 C, Sig=210,4 Ref=off

Peak #	RetTime [min]	Type	Width [min]	Area [mAU*s]	Height [mAU]	Area %
1	9.183	BV	0.2190	440.46912	30.77402	3.0544
2	9.746	VB	0.2390	1.39802e4	891.28406	96.9456

Racemic and Enantioenriched **19**

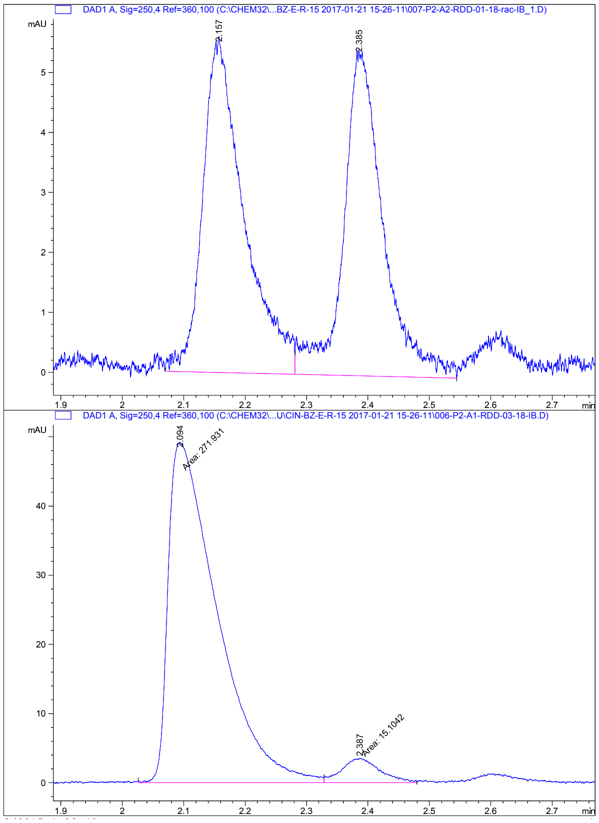

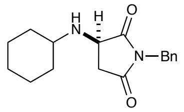

Peak #	RetTime [min]	Type	Width [min]	Area [mAU*s]	Height [mAU]	Area %
1	2.157	BV	0.0559	25.96450	5.56657	51.6035
2	2.385	VV R	0.0550	24.35085	5.36130	48.3965

Peak #	RetTime [min]	Type	Width [min]	Area [mAU*s]	Height [mAU]	Area %
1	2.094	MF	0.0921	271.93130	49.18518	94.7379
2	2.387	FM	0.0720	15.10422	3.49755	5.2621

Racemic and Enantioenriched **20**

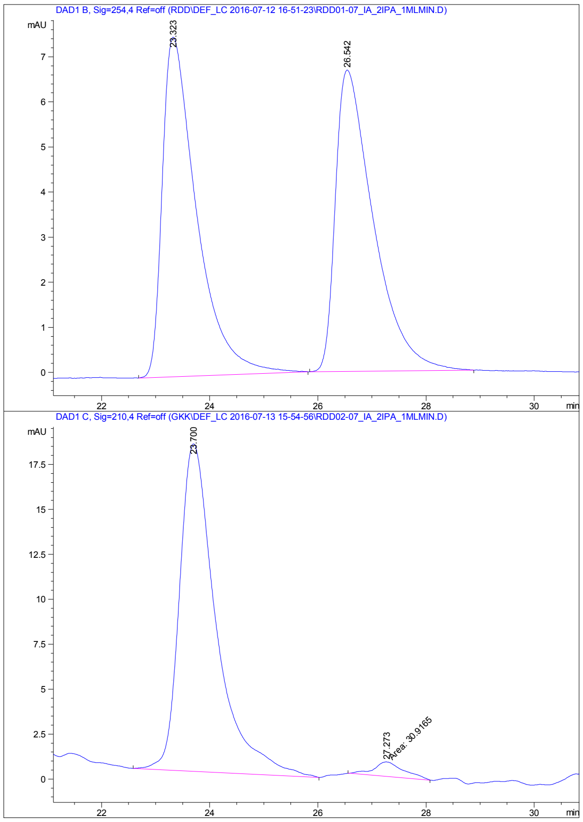

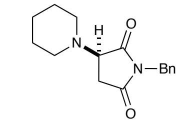

Signal 3: DAD1 C, Sig=210,4 Ref=off

Peak #	RetTime [min]	Type	Width [min]	Area [mAU*s]	Height [mAU]	Area %
1	23.323	BB	0.6516	1.26442e4	288.33511	50.1073
2	26.540	BB	0.7273	1.25901e4	256.22342	49.8927

Signal 3: DAD1 C, Sig=210,4 Ref=off

Peak #	RetTime [min]	Type	Width [min]	Area [mAU*s]	Height [mAU]	Area %
1	23.700	BB	0.6847	856.63623	18.21482	96.5167
2	27.273	MM	0.6432	30.91652	8.01082e-1	3.4833

Racemic and Enantioenriched **21**

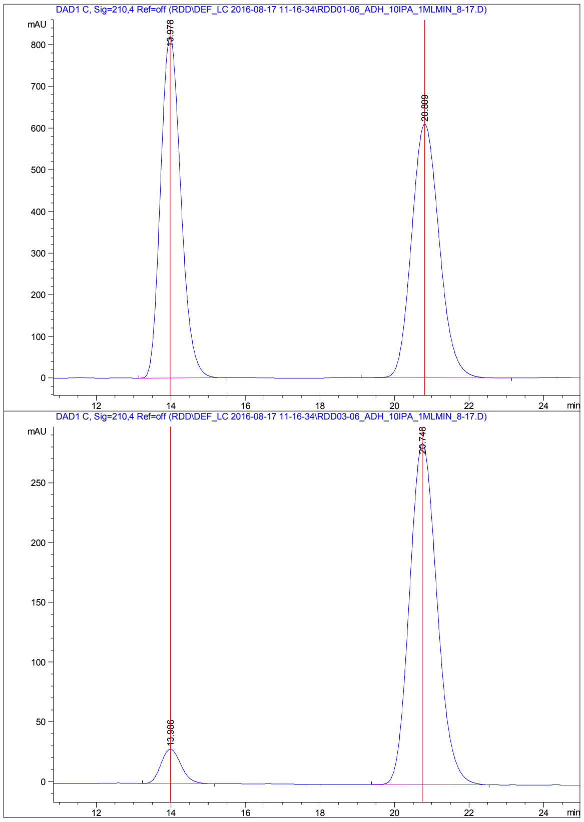

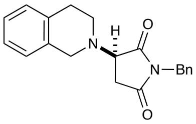

Signal 2: DAD1 C, Sig=210,4 Ref=off

Peak #	RetTime [min]	Type	Width [min]	Area [mAU*s]	Height [mAU]	Area %
1	13.978	BB	0.5953	3.09843e4	819.11908	49.6770
2	20.809	BB	0.8072	3.13872e4	609.10699	50.3230

Signal 2: DAD1 C, Sig=210,4 Ref=off

Peak #	RetTime [min]	Type	Width [min]	Area [mAU*s]	Height [mAU]	Area %
1	13.986	BB	0.5891	1076.27478	28.72956	6.8434
2	20.748	BB	0.8071	1.46510e4	285.32999	93.1566

Racemic and Enantioenriched **22**

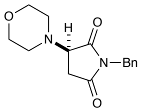

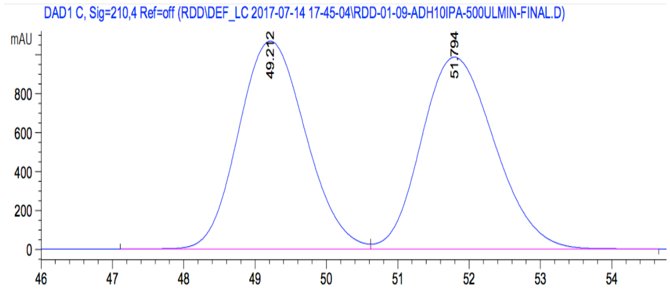

Signal 2: DAD1 C, Sig=210,4 Ref=off

Peak #	RetTime [min]	Type	Width [min]	Area [mAU*s]	Height [mAU]	Area %
1	49.212	BV	1.0179	6.98475e4	1066.78760	49.8499
2	51.794	VB	1.1091	7.02682e4	984.04779	50.1501
Totals :				1.40116e5	2050.83539	

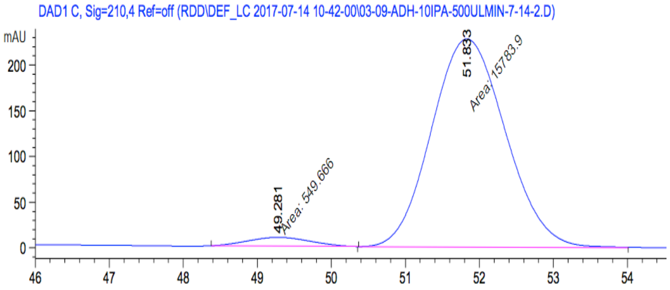

Signal 2: DAD1 C, Sig=210,4 Ref=off

Peak #	RetTime [min]	Type	Width [min]	Area [mAU*s]	Height [mAU]	Area %
1	49.281	MM	0.9598	549.66602	9.54489	3.3652
2	51.833	MM	1.1567	1.57839e4	227.41969	96.6348
Totals :				1.63336e4	236.96458	

Racemic and Enantioenriched **23**

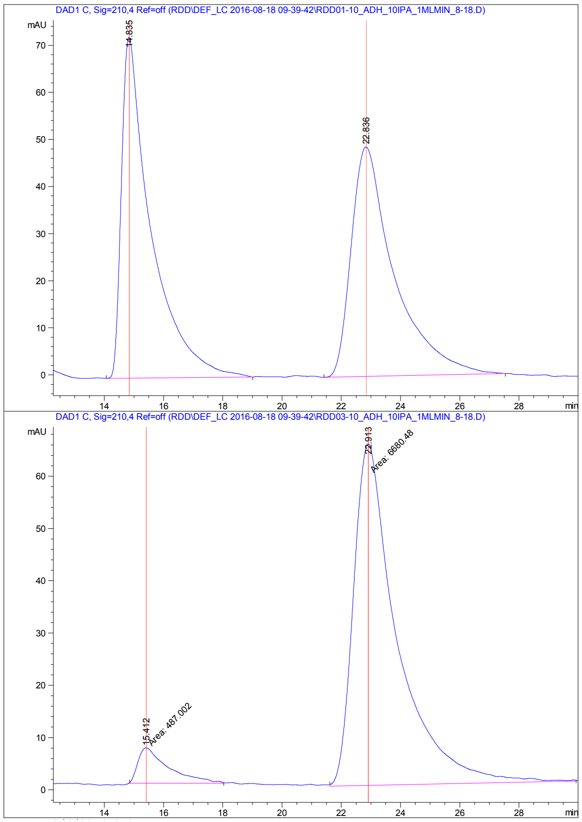

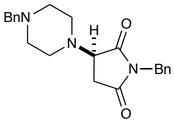

Signal 2: DAD1 C, Sig=210,4 Ref=off

Peak #	RetTime [min]	Type	Width [min]	Area [mAU*s]	Height [mAU]	Area %
1	14.835	BB	0.9519	4990.94092	72.42825	50.5357
2	22.836	BB	1.4051	4885.12939	48.74817	49.4643

Signal 2: DAD1 C, Sig=210,4 Ref=off

Peak #	RetTime [min]	Type	Width [min]	Area [mAU*s]	Height [mAU]	Area %
1	15.412	MM	1.1937	487.00238	6.79948	6.7946
2	22.913	MM	1.7035	6680.48145	65.35913	93.2054

Racemic and Enantioenriched **24**

-20 °C

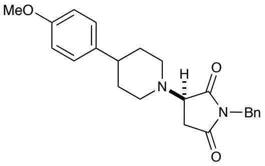

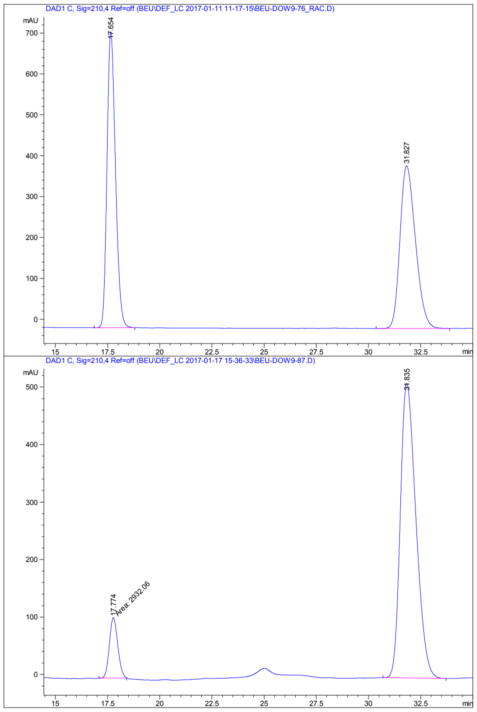

Signal 2: DAD1 C, Sig=210,4 Ref=off

Peak #	RetTime [min]	Type	Width [min]	Area [mAU*s]	Height [mAU]	Area %
1	17.654	BB	0.4382	2.03667e4	723.91608	49.8625
2	31.827	BB	0.7983	2.04790e4	397.99921	50.1375

Signal 2: DAD1 C, Sig=210,4 Ref=off

Peak #	RetTime [min]	Type	Width [min]	Area [mAU*s]	Height [mAU]	Area %
1	17.774	MM	0.4657	2932.06128	104.93056	9.9425
2	31.835	BB	0.8028	2.65582e4	512.30676	90.0575

-40 °C

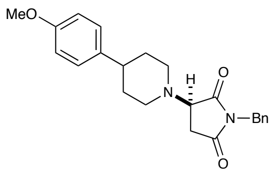

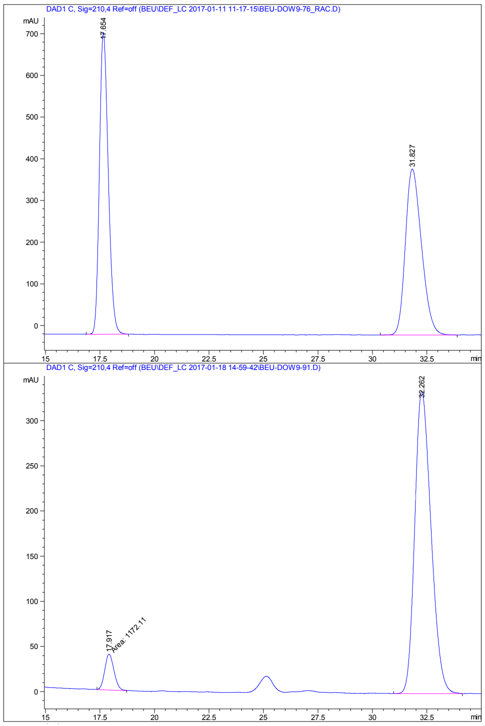

Signal 2: DAD1 C, Sig=210,4 Ref=off

Peak #	RetTime [min]	Type	Width [min]	Area [mAU*s]	Height [mAU]	Area %
1	17.654	BB	0.4382	2.03667e4	723.91608	49.8625
2	31.827	BB	0.7983	2.04790e4	397.99921	50.1375

Signal 2: DAD1 C, Sig=210,4 Ref=off

Peak #	RetTime [min]	Type	Width [min]	Area [mAU*s]	Height [mAU]	Area %
1	17.915	MM	0.4825	1140.01331	39.37619	6.1503
2	32.262	MM	0.8653	1.73959e4	335.07153	93.8497

Racemic and Enantioenriched **25**

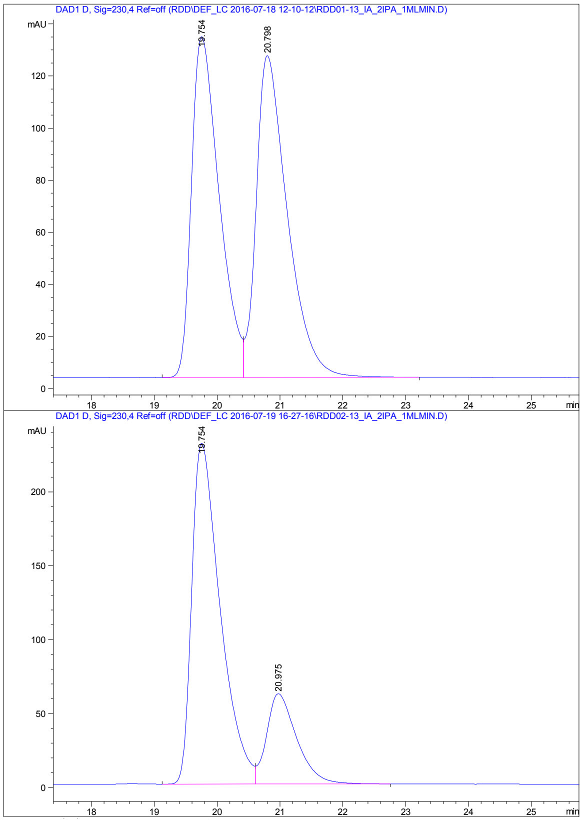

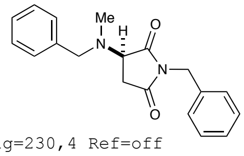

Signal 4: DAD1 D, Sig=230,4 Ref=off

Peak #	RetTime [min]	Type	Width [min]	Area [mAU*s]	Height [mAU]	Area %
1	19.754	BV	0.4637	4011.88379	130.86577	48.4948
2	20.798	VB	0.5143	4260.92627	123.55144	51.5052

Signal 4: DAD1 D, Sig=230,4 Ref=off

Peak #	RetTime [min]	Type	Width [min]	Area [mAU*s]	Height [mAU]	Area %
1	19.754	BV	0.4710	7256.53174	230.71039	78.1488
2	20.975	VB	0.4975	2029.00195	61.08556	21.8512

Racemic and Enantioenriched **26**

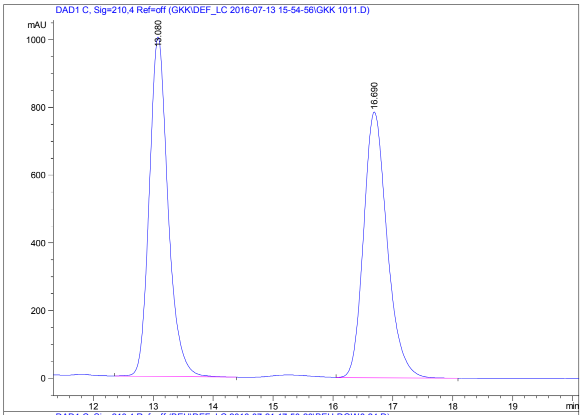

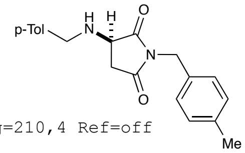

Signal 2: DAD1 C, Sig=210,4 Ref=off

Peak #	RetTime [min]	Type	Width [min]	Area [mAU*s]	Height [mAU]	Area %
1	13.080	BB	0.3302	2.18455e4	1002.63434	50.4142
2	16.690	VB	0.4191	2.14866e4	785.52014	49.5858

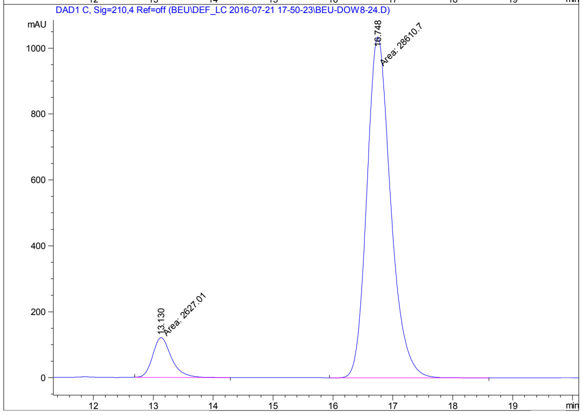

Signal 2: DAD1 C, Sig=210,4 Ref=off

Peak #	RetTime [min]	Type	Width [min]	Area [mAU*s]	Height [mAU]	Area %
1	13.130	MM	0.3626	2627.00659	120.75199	8.4097
2	16.748	MM	0.4608	2.86107e4	1034.79272	91.5903

Racemic and Enantioenriched **27**

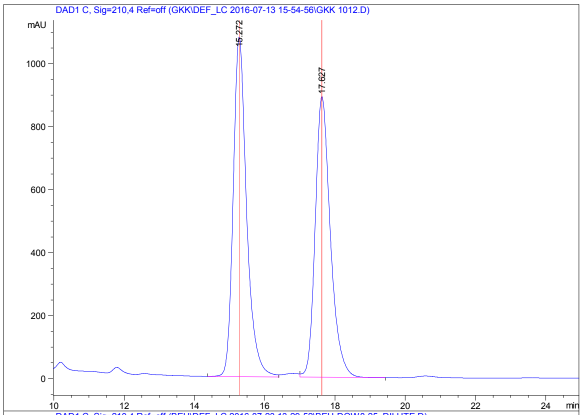

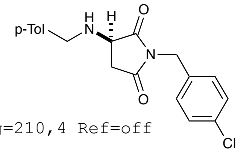

Signal 2: DAD1 C, Sig=210,4 Ref=off

Peak #	RetTime [min]	Type	Width [min]	Area [mAU*s]	Height [mAU]	Area %
1	15.272	BV	0.3997	2.86335e4	1078.62268	51.7079
2	17.627	VB	0.4561	2.67419e4	891.21863	48.2921

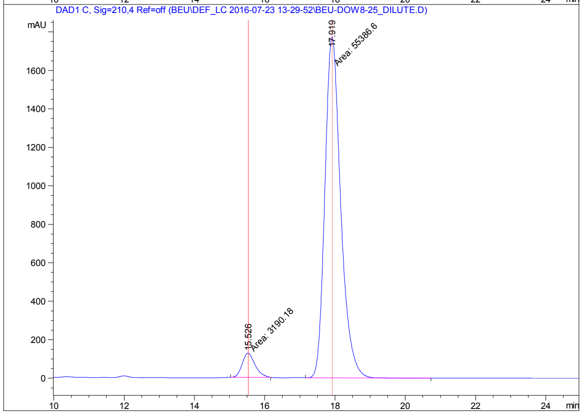

Signal 2: DAD1 C, Sig=210,4 Ref=off

Peak #	RetTime [min]	Type	Width [min]	Area [mAU*s]	Height [mAU]	Area %
1	15.526	MM	0.4228	3190.17920	125.76527	5.4461
2	17.919	MM	0.5209	5.53866e4	1772.05261	94.5539

Racemic and Enantioenriched **28**

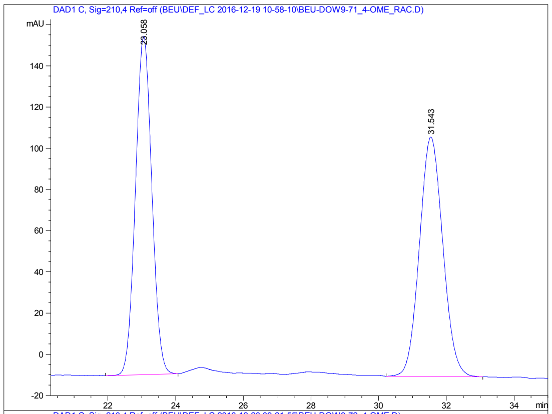

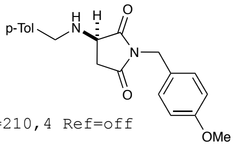

Signal 2: DAD1 C, Sig=210,4 Ref=off

Peak #	RetTime [min]	Type	Width [min]	Area [mAU*s]	Height [mAU]	Area %
1	23.058	BB	0.5263	5523.57617	164.27777	49.7196
2	31.543	BB	0.7464	5585.88477	116.29561	50.2804

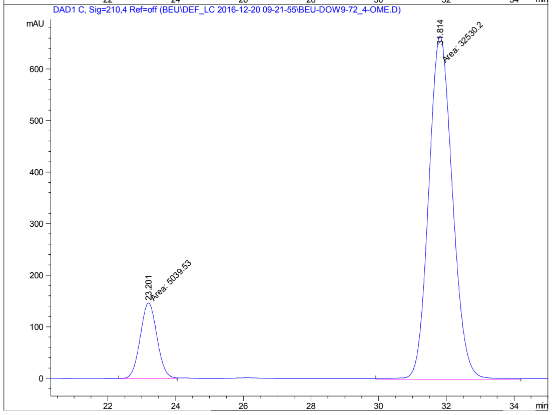

Signal 2: DAD1 C, Sig=210,4 Ref=off

Peak #	RetTime [min]	Type	Width [min]	Area [mAU*s]	Height [mAU]	Area %
1	23.201	MM	0.5728	5039.52979	146.64676	13.4138
2	31.814	MM	0.8147	3.25302e4	665.46429	86.5862

Racemic and Enantioenriched **29**

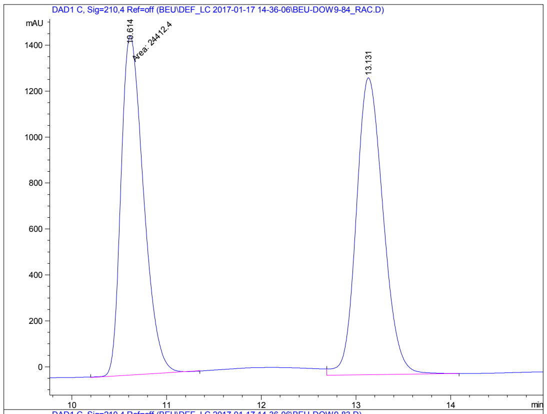

-20 °C

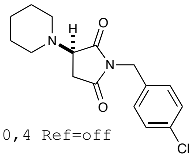

Signal 2: DAD1 C, Sig=210,4 Ref=off

Peak #	RetTime [min]	Type	Width [min]	Area [mAU*s]	Height [mAU]	Area %
1	10.614	MM	0.2755	2.44124e4	1476.93445	48.7928
2	13.131	VB	0.3093	2.56204e4	1292.45898	51.2072

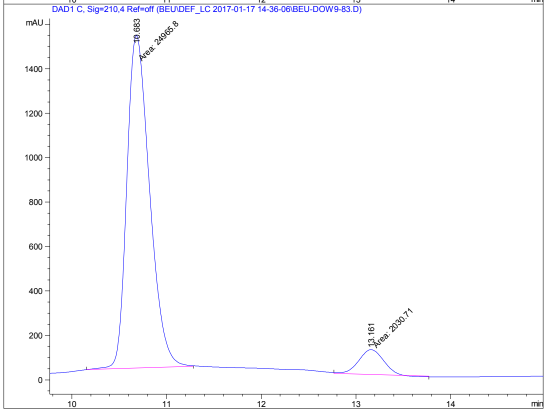

Signal 2: DAD1 C, Sig=210,4 Ref=off

Peak #	RetTime [min]	Type	Width [min]	Area [mAU*s]	Height [mAU]	Area %
1	10.683	MM	0.2780	2.49658e4	1496.76892	92.4779
2	13.161	MM	0.3043	2030.71130	111.20616	7.5221

-40 °C

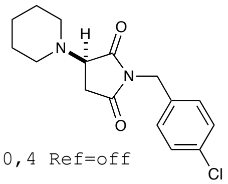

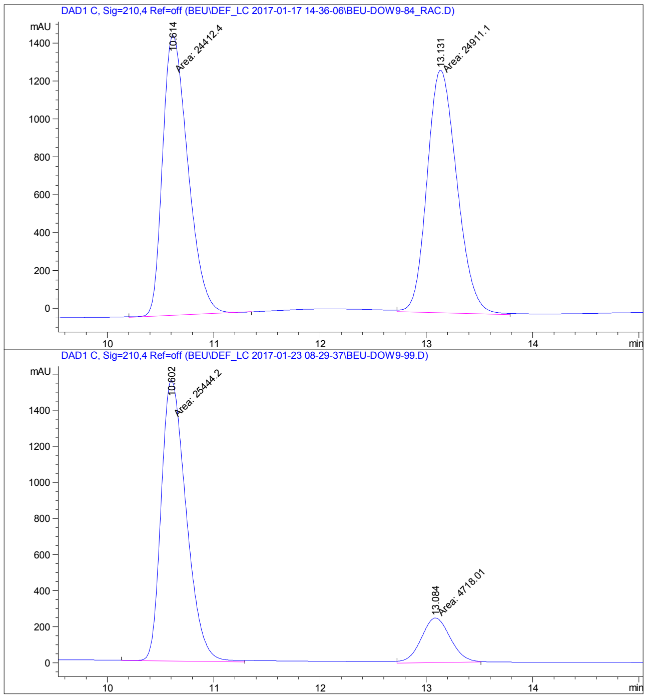

Signal 2: DAD1 C, Sig=210,4 Ref=off

Peak #	RetTime [min]	Type	Width [min]	Area [mAU*s]	Height [mAU]	Area %
1	10.614	MM	0.2755	2.44124e4	1476.93445	48.7928
2	13.131	VB	0.3093	2.56204e4	1292.45898	51.2072

Signal 2: DAD1 C, Sig=210,4 Ref=off

Peak #	RetTime [min]	Type	Width [min]	Area [mAU*s]	Height [mAU]	Area %
1	10.602	MM	0.2725	2.54442e4	1556.49866	84.3579
2	13.084	MM	0.3178	4718.01318	247.41553	15.6421

Racemic and Enantioenriched **30**

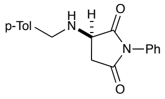

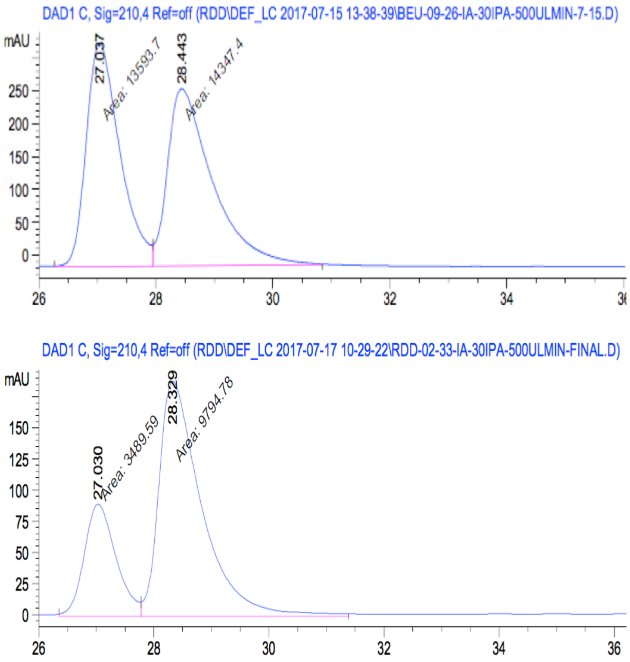

Signal 3: DAD1 C, Sig=210,4 Ref=off

Peak #	RetTime [min]	Type	Width [min]	Area [mAU*s]	Height [mAU]	Area %
1	27.037	MF	0.6661	1.35937e4	340.14880	48.6513
2	28.443	FM	0.8875	1.43474e4	269.42029	51.3487
Totals :				2.79411e4	609.56909	

Signal 3: DAD1 C, Sig=210,4 Ref=off

Peak #	RetTime [min]	Type	Width [min]	Area [mAU*s]	Height [mAU]	Area %
1	27.030	MF	0.6436	3489.58740	90.36543	26.2684
2	28.329	FM	0.8633	9794.77637	189.09468	73.7316
Totals :				1.32844e4	279.46011	

Racemic and Enantioenriched **31**

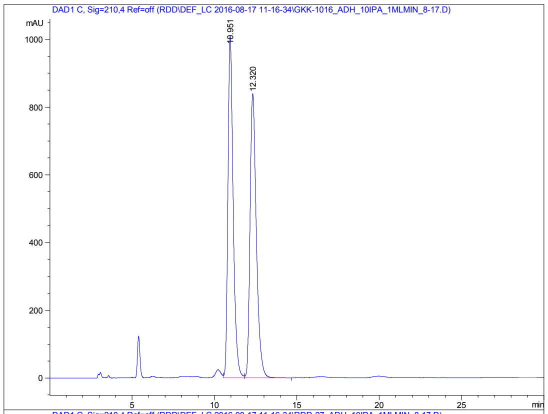

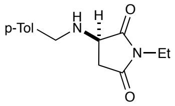

Signal 2: DAD1 C, Sig=210,4 Ref=off

Peak #	RetTime [min]	Type	Width [min]	Area [mAU*s]	Height [mAU]	Area %
1	10.951	VV	0.3361	2.23300e4	1009.49506	50.0033
2	12.320	VB	0.4045	2.23271e4	838.82086	49.9967

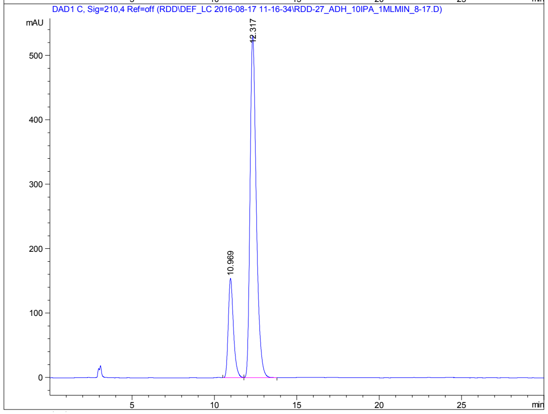

Signal 2: DAD1 C, Sig=210,4 Ref=off

Peak #	RetTime [min]	Type	Width [min]	Area [mAU*s]	Height [mAU]	Area %
1	10.969	BV	0.3317	3358.74561	154.44734	19.4415
2	12.317	VB	0.3995	1.39174e4	531.48181	80.5585

Racemic and Enantioenriched **34**

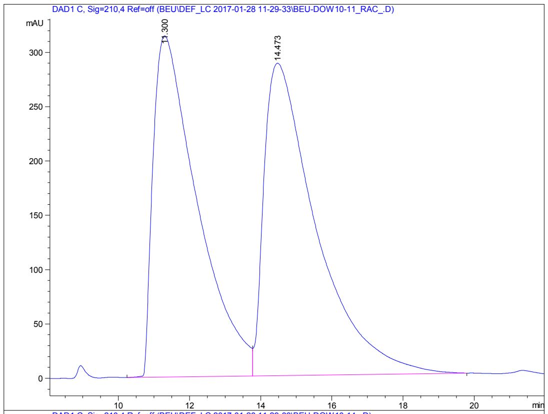

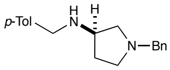

Signal 1: DAD1 C, Sig=210,4 Ref=off

Peak #	RetTime [min]	Type	Width [min]	Area [mAU*s]	Height [mAU]	Area %
1	11.300	BV	1.2625	2.71527e4	314.12094	48.1202
2	14.473	VB	1.4896	2.92742e4	287.57574	51.8798

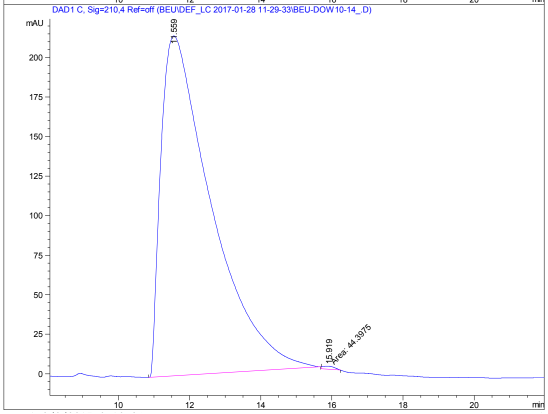

Signal 1: DAD1 C, Sig=210,4 Ref=off

Peak #	RetTime [min]	Type	Width [min]	Area [mAU*s]	Height [mAU]	Area %
1	11.559	BB	1.3959	2.11996e4	215.09189	99.7910
2	15.919	MM	0.3771	44.39747	1.96218	0.2090

Racemic and Enantioenriched **35**

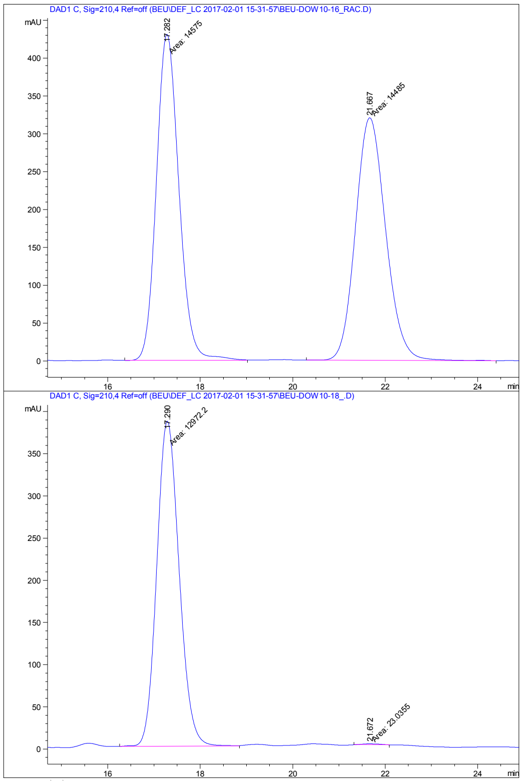

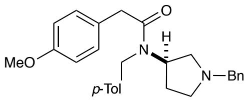

Signal 2: DAD1 C, Sig=210,4 Ref=off

Peak #	RetTime [min]	Type	Width [min]	Area [mAU*s]	Height [mAU]	Area %
1	17.282	MM	0.5642	1.45750e4	430.52969	50.1547
2	21.667	MM	0.7541	1.44850e4	320.14590	49.8453

Signal 2: DAD1 C, Sig=210,4 Ref=off

Peak #	RetTime [min]	Type	Width [min]	Area [mAU*s]	Height [mAU]	Area %
1	17.290	MM	0.5608	1.29722e4	385.51501	99.8227
2	21.672	MM	0.4016	23.03553	9.55973e-1	0.1773

Racemic and Enantioenriched **36**

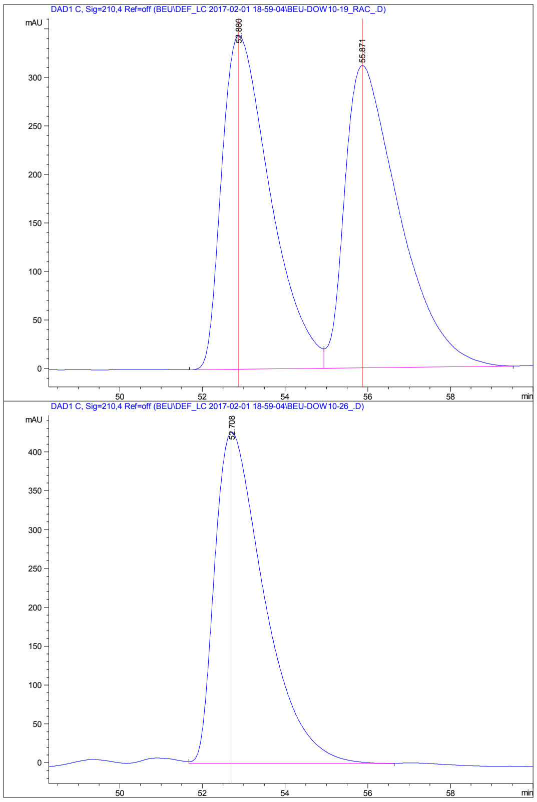

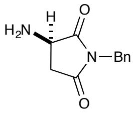

Signal 2: DAD1 C, Sig=210,4 Ref=off

Peak #	RetTime [min]	Type	Width [min]	Area [mAU*s]	Height [mAU]	Area %
1	52.880	BV	1.2273	2.80466e4	343.96411	49.5273
2	55.871	VB	1.2949	2.85820e4	311.37842	50.4727

Signal 2: DAD1 C, Sig=210,4 Ref=off

Peak #	RetTime [min]	Type	Width [min]	Area [mAU*s]	Height [mAU]	Area %
1	52.708	VB	1.2689	3.54968e4	426.52151	100.0000

Racemic and Enantioenriched **37**

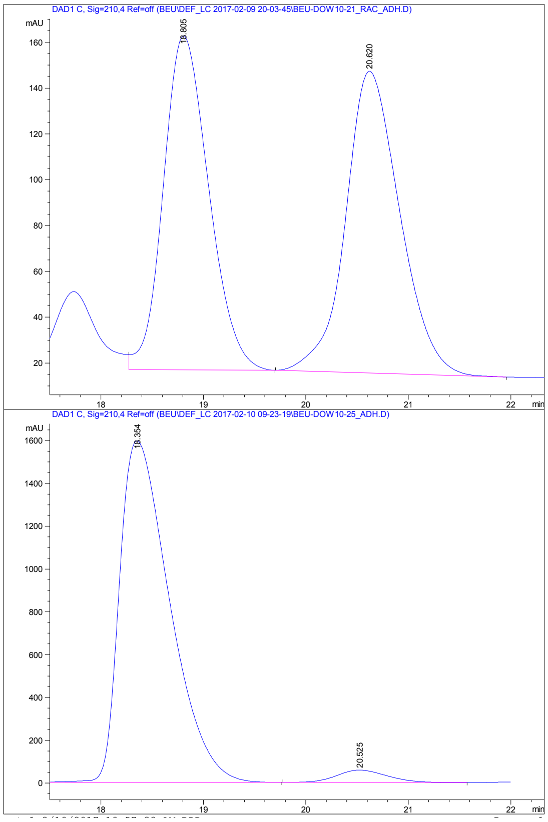

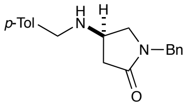

Signal 2: DAD1 C, Sig=210,4 Ref=off

Peak #	RetTime [min]	Type	Width [min]	Area [mAU*s]	Height [mAU]	Area %
1	18.805	VB	0.4777	4496.20557	145.84627	48.1582
2	20.620	BB	0.5626	4840.10938	131.76305	51.8418

Signal 2: DAD1 C, Sig=210,4 Ref=off

Peak #	RetTime [min]	Type	Width [min]	Area [mAU*s]	Height [mAU]	Area %
1	18.354	BB	0.5225	5.42645e4	1596.57056	96.4660
2	20.525	BB	0.5319	1987.94116	58.00003	3.5340
